# Region-specific forest structure pathways reveal climate effects on old-growth tropical forest biomass

**DOI:** 10.1038/s41598-026-61888-7

**Published:** 2026-09-01

**Authors:** Pauline Depoortere, David Bauman, Adeline Fayolle, Oliver Phillips, Lalasia Bialic-Murphy, Constantin M. Zohner, Arthur Vander Linden, Antoine Plumacker, Hugo de Lame, Jingjing Liang, Peter B. Reich, Javier G. P. Gamarra, Giorgio Alberti, Bruno Hérault, Nicolas Picard, Sergio de-Miguel, Gert-Jan Nabuurs, Thomas W. Crowther, Alain Paquette, Hans Pretzsch, Susan K. Wiser, Mo Zhou, Han Y. H. Chen, Hans Verbeeck, Charles De Cannière, Martin J. P. Sullivan, Lourens Poorter, Quentin Ponette, Marijn Bauters, Vincent Deblauwe, Gunnar Keppel, Francis Q. Brearley, Irié Casimir Zo-Bi, Tiodionwa Abdoulaye Ouattara, Michael J. Lawes, Frans Bongers, Jorge A. Meave, Mark Schulze, Michael Lyonga Ngoh, Hans ter Steege, Jürgen Homeier, Akomian Fortuné Azihou, Liam Trethowan, J. W. Ferry Slik, Géraldine Derroire, Damien Bonal, William F. Laurance, Andreas Hemp, Agustinus Murdjoko, Jesús Aguirre-Gutiérrez, Endang Kintamani, Lee White, Simon L. Lewis, Andrew Hector, Christian Amani, Ana Andrade, Albert Angbonga, Luiz Aragão, Ayushi Kurian, Félix Allah Barem, Angelica M. Almeyda Zambrano, Esteban Alvarez-Davila, Patricia Alvarez-Loayza, Elisée Amstrong, Alejandro Araujo-Murakami, Luzmila Arroyo, Timothy R. Baker, Ousmane Bako, Olaf Bánki, Christopher Baraloto, Nicolas Barbier, Jorcely Gonçalves Barroso, Patrick Bassama, Hans Beeckman, Nithanel Mikael Hendrik Benu, Hervé Bihiya, Philippe Birnbaum, Robert Bitariho, Pascal Boeckx, Kelly Boekee, Jan Bogaert, Katrin Böhning-Gease, Faustin Boyemba, Hilândia Brandão da Cunha, Roel Brienen, Jaime Briseño-Reyes, Eben N. Broadbent, Jose Luis Camargo, Benoît Cassart, Roberto Cazzolla Gatti, Jérôme Chave, George Chuyong, David B. Clark, Connie Clark, Alessio Collalti, Murray Collins, Fernando Cornejo Valverde, Jose Javier Corral-Rivas, Pierre Couteron, Aida Cuni-Sanchez, Lôla da Costa, Jose Daniel Soto, Ashaq Ahmad Dar, Javid Ahmad Dar, Gilles Dauby, Thales de Haulleville, Anthony Di Fiore, John Diisi, Marie-Noel Djuikouo K., Jiri Dolezal, Narcisse Dongmo, Jean-Louis Doucet, Vincent Droissart, Julien Engel, Nestor Laurier Engone Obiang, Santiago Espinosa, Teresa Eyre, Sophie Fauset, Ted R. Feldpausch, Markus Fischer, Krisna Gajapersad, Deden Girmansyah, Emanuel Gloor, Ieda Gomes do Amaral, Christelle Flore Gonmadje, Elisa Grieco, James Grogan, Richard Habonayo, David J. Harris, Eurídice Honorio Coronado, Isau Huamantupa-Chuquimaco, Wannes Hubau, Patrick A. Jansen, Kathryn J. Jeffery, Blaise Jumbam, Naveen Babu Kanda, Narcisse Kamdem, Emmanuel Kasongo Yakusu, John Katembo, Charles Kayijamahe, Elizabeth Kearsley, David Kenfack, Mohammed Latif Khan, Timothy Killeen, Subashree Kothandaraman, Ronald Kougue, Amit Kumar, Relawan Kuswandi, Nicolas Labrière, David Lasso, Susan Laurance, Miguel Leal, Hugo Leblanc, Moses Libalah, Janvier Lisingo, Yadvinder Malhi, Beatriz S. Marimon, Ben Hur Marimon Junior, Andrew R. Marshall, Emanuel H. Martin, Olivier Martin-Ducup, Michel M. Mbasi, Faustin Mbayu, Casimiro Mendoza, Irina Mendoza Polo, Gislain Mofack, Abel Monteagudo-Mendoza, Arafat Mtui, S. Muthuramkumar, Ayyappan Narayanan, María Guadalupe Nava-Miranda, David Neill, Victor John Neldner, Eric Ngansop Tchatchouang, Fernandez Ngoula, Michael R. Ngugi, Moses Nsanyi Sainge, Percy Núñez Vargas, Edgar Ortiz-Malavassi, Nadir Pallqui Camacho, Vivek Pandi, Germaine Alexander Parada Gutierrez, Guido Pardo, Narayanaswamy Parthasarathy, Raphael Pelissier, Sebastian Pfautsch, Bryan C. Pijanowski, Daniel Piotto, John Pipoly, Nigel Pitman, Pierre Ploton, John R. Poulsen, B. R. Ramesh, Freddy Ramirez Arevalo, Hirma Ramírez-Angulo, L. Rasingam, Maxime Réjou-Méchain, Zorayda Restrepo Correa, Wilson Roberto Spironello, Cintia Rodrigues de Souza, Lily O. Rodriguez, Samir Rolim, Anand Roopsind, Francesco Rovero, Le Bienfaiteur Sagang, Purabi Saikia, Antonello Salis, Benoit Patrick Salla, Javier Eduardo Silva Espejo, Marcos Silveira, Murielle Simo-Droissart, Nelly Sirri, Bonaventure Sonké, Somaiah Sundarapandian, Michael Swaine, Ben Swanepoel, Hermann Taedoumg, Imma Tcheferi, John Terborgh, Duncan Thomas, Raquel Thomas, John Tshibamba Mukendi, Peter M. Umunay, Renato Valencia, Luis Valenzuela Gamarra, Leandro Valle Ferreira, Geertje van der Heijden, Rodolfo Vasquez Martinez, José Daniel Vega-Nieva, Emilio Vilanova, Braulio Vilchez Alvarado, Barbara Vinceti, Jason Vleminckx, Klaus von Gadow, Vincent Antoine Vos, Eric Wasingya, James Watson, Verginia Wortel, Roderick Zagt, Donatien Zébazé, GFBI, Oliver Phillips, Lalasia Bialic-Murphy, Constantin M. Zohner, Jingjing Liang, Peter B. Reich, Giorgio Alberti, Bruno Hérault, Nicolas Picard, Sergio de-Miguel, Gert-Jan Nabuurs, Thomas W. Crowther, Alain Paquette, Hans Pretzsch, Mo Zhou, Hans Verbeeck, Charles De Cannière, Lourens Poorter, Marijn Bauters, Gunnar Keppel, Francis Q. Brearley, Irié Casimir Zo-Bi, Michael J. Lawes, Frans Bongers, Jorge A. Meave, Hans ter Steege, Akomian Fortuné Azihou, Liam Trethowan, Géraldine Derroire, Andreas Hemp, Agustinus Murdjoko, Jesús Aguirre-Gutiérrez, Endang Kintamani, Simon L. Lewis, Andrew Hector, Christian Amani, Luiz Aragão, Esteban Alvarez-Davila, Patricia Alvarez-Loayza, Alejandro Araujo-Murakami, Luzmila Arroyo, Timothy R. Baker, Olaf Bánki, Jorcely Gonçalves Barroso, Hans Beeckman, Nithanel Mikael Hendrik Benu, Philippe Birnbaum, Robert Bitariho, Pascal Boeckx, Kelly Boekee, Jan Bogaert, Hilândia Brandão da Cunha, Roel Brienen, Jaime Briseño-Reyes, Eben N. Broadbent, Roberto Cazzolla Gatti, George Chuyong, David B. Clark, Connie Clark, Fernando Cornejo Valverde, Jose Javier Corral-Rivas, Aida Cuni-Sanchez, Lôla da Costa, Jose Daniel Soto, Ashaq Ahmad Dar, Javid Ahmad Dar, Thales de Haulleville, John Diisi, Jiri Dolezal, Nestor Laurier Engone Obiang, Santiago Espinosa, Teresa Eyre, Ted R. Feldpausch, Markus Fischer, Krisna Gajapersad, Deden Girmansyah, Ieda Gomes do Amaral, Richard Habonayo, David J. Harris, Eurídice Honorio Coronado, Wannes Hubau, Patrick A. Jansen, Blaise Jumbam, Emmanuel Kasongo Yakusu, Charles Kayijamahe, Elizabeth Kearsley, David Kenfack, Mohammed Latif Khan, Timothy Killeen, Subashree Kothandaraman, Amit Kumar, Relawan Kuswandi, David Lasso, Moses Libalah, Janvier Lisingo, Yadvinder Malhi, Beatriz S. Marimon, Ben Hur Marimon Junior, Andrew R. Marshall, Emanuel H. Martin, Faustin Mbayu, Casimiro Mendoza, Irina Mendoza Polo, Abel Monteagudo-Mendoza, Arafat Mtui, María Guadalupe Nava-Miranda, David Neill, Victor John Neldner, Michael R. Ngugi, Moses Nsanyi Sainge, Percy Núñez Vargas, Edgar Ortiz-Malavassi, Germaine Alexander Parada Gutierrez, Guido Pardo, Narayanaswamy Parthasarathy, Sebastian Pfautsch, Bryan C. Pijanowski, Daniel Piotto, John Pipoly, Nigel Pitman, John R. Poulsen, Freddy Ramirez Arevalo, Zorayda Restrepo Correa, Wilson Roberto Spironello, Samir Rolim, Anand Roopsind, Francesco Rovero, Purabi Saikia, Antonello Salis, Javier Eduardo Silva Espejo, Marcos Silveira, Bonaventure Sonké, Somaiah Sundarapandian, Michael Swaine, Ben Swanepoel, Raquel Thomas, John Tshibamba Mukendi, Peter M. Umunay, Renato Valencia, Leandro Valle Ferreira, Rodolfo Vasquez Martinez, José Daniel Vega-Nieva, Braulio Vilchez Alvarado, Jason Vleminckx, Klaus von Gadow, Vincent Antoine Vos, Eric Wasingya, James Watson, Verginia Wortel, Roderick Zagt, Javier G.P. Gamarra, Han Y.H. Chen, J.W.Ferry Slik, Angelica M.Almeyda Zambrano, Jean-François Bastin, Jean-François Bastin

**Affiliations:** 1 TERRA Teaching and Research Centre, Gembloux Agro-Bio Tech, Université de Liège, Gembloux, Belgium; 2 AMAP, CIRAD, CNRS, INRAE, IRD, Université de Montpellier, Montpellier, France; 3 Laboratoire d’Ecologie Végétale Et Biogéochimie, Université Libre de Bruxelles, Brussels, Belgium; 4 Forêts Et Sociétés, CIRAD, Montpellier, France; 5 Institut de Recherche en Écologie Tropicale (IRET), CENAREST, Libreville, Gabon; 6 School of Geography, University of Leeds, Leeds, UK; 7 Swiss Federal Institute for Forest, Snow and Landscape Research, Land Change Science Unit, 8903 Birmensdorf, Switzerland; 8 Institute for Future Initiatives, University of Tokyo (UTokyo), Tokyo, Japan; 9 BRANCH Institute, 6300 Zug, Switzerland; 10 Department of Forestry and Natural Resources, Purdue University, West Lafayette, IN USA; 11 Institute for Global Change Biology, University of Michigan, Ann Arbor, MI 48109 USA; 12 Department of Forest Resources, University of Minnesota, St. Paul, MN 55108 USA; 13 Forestry Division, Food and Agriculture Organization of the United Nations, Rome, Italy; 14 Department of Agricultural, Food, Environmental and Animal Sciences (Di4A), University of Udine, Udine, Italy; 15 National Biodiversity Future Centre, Palermo, Italy; 16 Forêts Et Sociétés, Univ Montpellier, CIRAD, Montpellier, France; 17 GIP Ecofor, 42 Rue Scheffer, 75116 Paris, France; 18 Department of Agricultural and Forest Sciences and Engineering, University of Lleida, Lleida, Spain; 19 Forest Science and Technology Centre of Catalonia (CTFC), Solsona, Spain; 20 Wageningen University and Research, Wageningen, The Netherlands; 21 Biological and Environmental Science and Engineering Division, KAUST, Thuwal, Kingdom of Saudi Arabia; 22 Centre for Forest Research, Université du Québec À Montréal, Montreal, Canada; 23 Tree Growth and Wood Physiology, School of Life Sciences Weihenstephan, Technical University of Munich, Hans-Carl-Von-Carlowitz-Platz 2, 85354 Freising, Germany; 24 SMART Ecosystems Group, Sustainable Forest Management Research Institute iuFOR, University of Valladolid, Avda de Madrid S/N, Palencia, Spain; 25 Manaaki Whenua–Landcare Research, Lincoln, New Zealand; 26 Inner Mongolia Agr Univ, Coll Grassland Sci, Hohhot, 010011 China; 27 Faculty of Natural Resources Management, Lakehead University, Thunder Bay, ON Canada; 28 Q-ForestLab, Department of Environment, Ghent University, Ghent, Belgium; 29 Brussels Bioengineering School, Université Libre de Bruxelles, Bruxelles, Belgium; 30 Department of Natural Sciences, Manchester Metropolitan University, Manchester, UK; 31 Forest Ecology and Forest Management Group, Wageningen University & Research, Droevendaalsesteeg 3, 6708 PB Wageningen, The Netherlands; 32 Earth and Life Institute, Environmental Sciences, Université Catholique de Louvain (UCLouvain), Croix du Sud, 2 Box L7.05.24, 1348 Louvain-La-Neuve, Belgium; 33 International Institute of Tropical Agriculture, Yaoundé, Cameroon; 34 University of California, Los Angeles, Los Angeles, USA; 35 School of Biological Sciences and Environment Institute, Adelaide University, Adelaide, Australia; 36 UMRI Sciences Agronomiques Et Procédés de Transformation, Institut National Polytechnique Félix Houphouët-Boigny, BP 1093, Yamoussoukro, Côte d’Ivoire; 37 School of Life Sciences, University of KwaZulu-Natal, Scottsville, 3209 South Africa; 38 Institute of Biodiversity and Environmental Conservation (IBEC), Universiti Malaysia Sarawak, 94300 Kota Samarahan, Sarawak Malaysia; 39 Departamento de Ecología y Recursos Naturales, Facultad de Ciencias, Universidad Nacional Autónoma de México, 04510 Coyoacán, Ciudad de México Mexico; 40 Oregon State University, Corvallis, USA; 41 Department of Ecology and Evolutionary Biology, University of Tennessee Knoxville, Knoxville, USA; 42 Naturalis Biodiversity Center, Leiden, The Netherlands; 43 Quantitative Biodiversity Dynamics, Dept. of Biology, Utrecht University, Utrecht, The Netherlands; 44 Conservation Ecology, Philipps-Universität Marburg, Karl-Von-Frisch-Straße 8, 35043 Marburg, Germany; 45 Faculty of Resource Management, HAWK University of Applied Sciences and Arts, 37075 Göttingen, Germany; 46 Plant Ecology, Georg-August University of Göttingen, Untere Karspuele 2, 37073 Göttingen, Germany; 47 Laboratory of Applied Ecology, Faculty of Agricultural Sciences, University of Abomey-Calavi, 01 BP 526 Cotonou, Benin; 48 Herbarium Kew, Richmond, London TW9 3AE UK; 49 Environmental and Life Sciences Programme, Faculty of Science, Universiti Brunei Darussalam, Jalan Tungku Link, Gadong, Bandar Seri Begawan, BE1410 Brunei Darussalam; 50 Cirad, UMR EcoFoG (AgroParistech, CNRS, INRAE, Université Des Antilles, Université de La Guyane), Campus Agronomique, Kourou, French Guiana; 51 Department of Forestry, University of Brasilia, Campus Darcy Ribeiro, Brasília, 70.900-910 Brazil; 52 INRAE, UMR Silva, 54000 Nancy, France; 53 Centre for Tropical Environmental and Sustainability Science, College of Science and Engineering, James Cook University, Cairns, Australia; 54 Dept. Ecology of Fungi, University of Bayreuth, 95440 Bayreuth, Germany; 55 Fakultas Kehutanan, Universitas Papua, Jalan Gunung Salju Amban, Manokwari Papua Barat, Indonesia; 56 Georgina Mace Centre for the Living Planet, Imperial College London, Ascot, UK; 57 Environmental Change Institute, School of Geography and the Environment, University of Oxford, Oxford, UK; 58 Leverhulme Centre for Nature Recovery, University of Oxford, Oxford, UK; 59 Research Center for Biota Systems, National Research and Innovation Agency (BRIN), Cibinong, Indonesia; 60 Institut de Recherche en Écologie Tropicale, Libreville, Gabon; 61 School of Natural Sciences, University of Stirling, Stirling, UK; 62 Ministry of Forests, Seas, Environment and Climate, Libreville, Gabon; 63 Department of Geography, University College London, London, UK; 64 Department of Biology and Leverhulme Centre for Nature Recovery, University of Oxford, Oxford, UK; 65 Faculté Des Sciences, Université Officielle de Bukavu, Bukavu, Democratic Republic of the Congo; 66 National Institute of Amazonian Research (INPA), Manaus, Brazil; 67 Institut Facultaire Des Sciences Agronomiques de Yangambi, Kisangani, Democratic Republic of the Congo; 68 Earth Observation and Geoinformatics Division, National Institute for Space Research, São José Dos Campos, São Paulo Brazil; 69 Geography, Faculty of Environment, Science and Economy, University of Exeter, Exeter, EX4 4RJ UK; 70 Department of Agriculture, Ecotrophology, and Landscape Development, Anhalt University of Applied Sciences, Strenzfelder Allee 28, 06406 Bernburg, Germany; 71 Department of Ecology, French Institute of Pondicherry, Puducherry, India; 72 Institut Centrafricain de La Recherche Agronomique (ICRA), Bangi, Central African Republic; 73 Spatial Ecology and Conservation Lab, University of Florida, Gainesville, FL 32611 USA; 74 Fundación ConVida, Cl. 9 # 43A – 33, El Poblado, Medellín, Colombia; 75 Field Museum of Natural History, Chicago, IL USA; 76 Plant Systematics and Ecology Laboratory, Higher Teachers’ Training College, University of Yaoundé I, P.O. Box 047, Yaoundé, Cameroon; 77 Museo de Historia Natural Noel Kempff Mercado, Universidad Autónoma Gabriel René Moreno, Santa Cruz, Bolivia; 78 Ecole Nationale des Eaux Et Forêts de Mbalmayo, Ministère Des Forêts Et De La Faune, Mbalmayo, Cameroun; 79 Catalogue of Life Foundation, Amsterdam, The Netherlands; 80 Department of Biological Sciences, Institute of Environment, Florida International University, Miami, FL USA; 81 Universidade Federal Do Acre, Centro Multicisciplinar, Cruzeiro Do Sul, Acre Brazil; 82 Service of Wood Biology, Royal Museum for Central Africa, Tervuren, Belgium; 83 Balai Perhutanan Sosial (BPS) Manokwari, Jalan Inamberi-Susweni, Papua Barat, Indonesia; 84 Institute of Tropical Forest Conservation, Mbarara University of Science and Technology, Kabale, Uganda; 85 Isotope Bioscience Laboratory, Department of Green Chemistry and Technology, Ghent University, Gent, Belgium; 86 Department of Environmental Sciences, Wageningen University & Research, Wageningen, The Netherlands; 87 Helmholtz Center for Environmental Research UFZ, Permosterstraße 15, 04318 Leipzig, Germany; 88 Faculty of Life Sciences, Leipzig University, Talstraße 33, 04103 Leipzig, Germany; 89 Secrétariat Exécutif, Fonds National REDD+ (FONAREDD), Kinshasa, Democratic Republic of the Congo; 90 Facultad de Ciencias Forestales y Ambientales, Universidad Juárez del Estado de Durango, 34120 Durango, Mexico; 91 BIOME Lab, One Health Artificial Intelligence (OHAI) Research Hub, Department of Biological, Geological, and Environmental Sciences, Alma Mater Studiorum University of Bologna, Bologna, Italy; 92 UMR5300, Centre Recherche Biodiversité Environnement, CNRS, Université Paul Sabatier, IRD, INPT, Toulouse, France; 93 Department of Plant Science, University of Buea, PO Box 63, Buea, Cameroon; 94 Department of Biology, University of Missouri-St Louis, St Louis, MO USA; 95 Nicholas School of the Environment, Duke University, Durham, NC USA; 96 Forest Modelling Laboratory, National Research Council of Italy, Institute for Agriculture and Forestry Systems in the Mediterranean (CNR-ISAFOM), Via Madonna Alta 128, 06128 Perugia, Italy; 97 Space Intelligence Ltd, Henry Dunant House, 120 George Street, Edinburgh, EH2 4LH UK; 98 Andes to Amazon Biodiversity Program, Puerto Maldonado, Peru; 99 Department of International Environment and Development Studies (Noragric), Faculty of Landscape and Society, Norwegian University of Life Sciences (NMBU), Ås, Norway; 100 Department of Environment and Geography, University of York, York, UK; 101 Universidade Federal Do Pará, Instituto de Geociências, Faculdade de Meteorologia, Belém, Pará, Brasil; 102 Department of Ecology and Environmental Sciences, Pondicherry University, Puducherry, India; 103 Division of Natural Resource Management, Faculty of Forestry, Sher-E-Kashmir University of Agricultural Sciences and Technology of Kashmir, Benhama Ganderbal, Ganderbal, Jammu & Kashmir 191201 India; 104 Terrestrial Ecology and Modelling (TEAM) Lab, Department of Environmental Science and Engineering, SRM University-AP, Amaravati, 522240 Andhra Pradesh India; 105 Centre for Geospatial Technology, SRM University-AP, Amaravati, 522240 Andhra Pradesh India; 106 Primate Molecular Ecology and Evolution Laboratory, Department of Anthropology, The University of Texas, Austin, TX 78712 USA; 107 Tiputini Biodiversity Station, Universidad San Francisco de Quito, Cumbayá, Ecuador; 108 National Forest Authority, Kampala, Uganda; 109 University of Buea, Buea, Cameroon; 110 Institute of Botany of the Czech Academy of Sciences, Průhonice, Czech Republic; 111 University of South Bohemia, České Budějovice, Czech Republic; 112 AMAP, IRD, Herbier de Guyane, Cayenne, French Guiana; 113 Institut de Pharmacopée Et de Médecine Traditionnelle (IPHAMETRA), Centre National de La Recherche Scientifique Et Technologique (CENAREST), Libreville, Gabon; 114 Universidad Autónoma de San Luis Potosí, San Luis Potosí, Mexico; 115 Pontificia Universidad Católica del Ecuador, Quito, Ecuador; 116 Queensland Department of the Environment, Tourism, Science and Innovation, Brisbane, Australia; 117 School of Geography, Earth and Environmental Sciences, University of Plymouth, Plymouth, UK; 118 Institute of Plant Sciences, University of Bern, Bern, Switzerland; 119 Conservation International, Paramaribo, Suriname; 120 Research Center for Biosystematics and Evolution, National Research and Innovation Agency (BRIN), Cibinong, Indonesia; 121 University of Yaounde I, Yaoundé, Cameroon; 122 Botanic Garden of Smith College, Northampton, MA USA; 123 Département Des Sciences Agro-Environnementales, Faculté d’Agronomie Et de Bio-Ingénierie, Université du Burundi, Bujumbura, Burundi; 124 Royal Botanic Garden Edinburgh, Edinburgh, UK; 125 Royal Botanic Gardens, Kew, Richmond, London, UK; 126 Departamento de Biología. Universidad Nacional Amazónica de Madre de Dios (UNAMAD), Herbario Alwyn Gentry (HAG), Av. Jorge Chávez 1160. Puerto Maldonado, Madre de Dios, Perú; 127 Laboratory of Wood Technology, Department of Environment, Faculty of Bioscience Engineering, UGent-Woodlab, Ghent University, Ghent, Belgium; 128 CTFS-ForestGEO, Smithsonian Tropical Research Institute, Ancon, Panama; 129 Department of Biology, Utrecht University, Utrecht, The Netherlands; 130 Faculty of Natural Sciences, University of Stirling, Stirling, UK; 131 Department of Plant Sciences and Plant Pathology, Montana State University, Bozeman, USA; 132 National Biobank of Thailand, National Science and Technology Development Agency, Pathum Thani, 12120 Thailand; 133 Department of Ecology, French Institute of Pondicherry, 11, Saint Louis Street, White Town, Puducherry, 605001 India; 134 Faculté de Gestion Des Ressources Naturelles Renouvelables, Université de Kisangani, Kisangani, Democratic Republic of the Congo; 135 Institut Supérieur d’Etudes Agronomiques de Bengamisa, Bengamisa, Democratic Republic of the Congo; 136 International Crane Foundation & Endangered Wildlife Trust, Kigali, Rwanda; 137 BlueGreen Labs, Melsele, Belgium; 138 Department of Botany, Dr. Harisingh Gour Vishwavidyalaya (A Central University), Sagar, 470003 Madhya Pradesh India; 139 Department of Geography, Institute of Science, Banaras Hindu University, Varanasi, 221005 India; 140 Research Center for Ecology, National Research and Innovation AGENCY (BRIN), Cibinong, Indonesia; 141 Centre de Recherche Sur La Biodiversité Et L’Environnement (CRBE), Université de Toulouse, Toulouse INP, CNRS, IRD, Toulouse, France; 142 University of Calgary, Calgary, AB Canada; 143 Climate Smart, Hyderabad, India; 144 Laboratoire d’Écologie Et Aménagement Forestier, Département d’Ecologie Et de Gestion Des Ressources Végétales, Université de Kisangani, Kisangani, Democratic Republic of Congo; 145 Universidade Do Estado de Mato Grosso, Programa de Pós Graduação Em Ecologia E Conservação, Campus de Nova Xavantina, Mato Grosso, Brazil; 146 Forest Research Institute, University of the Sunshine Coast, Maroochydore, QL Australia; 147 Flamingo Land Ltd, Kirby Misperton, UK; 148 College of African Wildlife Management, Mweka, P.O. Box 3031, Moshi, Tanzania; 149 CIRAD, UMR AMAP, F‐34398 Montpellier, France; 150 Laboratory of Ecology and Sustainable Forest Management (LECAFOR), University of Kisangani, Kisangani, Democratic Republic of the Congo; 151 Forest Management in Bolivia, Sacta, Bolivia; 152 ColTree, Medellín, Colombia; 153 Jardín Botánico de Medellín, Medellín, Colombia; 154 Jardín Botánico de Missouri, Oxapampa, Peru; 155 Universidad Nacional de San Antonio Abad del Cusco, Cusco, Peru; 156 Mazingira Alliance for Community and Conservation (MACCO), Box 10, Mang’ula, Tanzania; 157 Department of Botany, VHNSN College, Virudhunagar, Tamilnadu India; 158 Escuela Politécnica Superior de Ingeniería, Campus Terra, Universidad de Santiago de Compostela, 27002 Lugo, Spain; 159 Colegio de Ciencias y Humanidades, Universidad Juárez del Estado de Durango, 34120 Durango, Mexico; 160 Facultad de Ingeniería Ambiental, Universidad Estatal Amazónica, Puyo, Ecuador; 161 Department of Environment and Science, Queensland Herbarium, Toowong, QL Australia; 162 National Herbarium of Cameroon, Po. Box 1601, Yaoundé, Cameroon; 163 Forestry School, Tecnológico de Costa Rica TEC, Cartago, Costa Rica; 164 Manipal Centre for Natural Sciences, Centre of Excellence, Manipal Academy of Higher Education, Manipal, Karnataka India; 165 Carrera de Ingeniería Forestal, Universidad Autónoma del Beni José Ballvián, Riberalta, Beni Bolivia; 166 Urban Transformations Research Centre, Western Sydney University, Locked Bag 1797, Penrith, NSW 2751 Australia; 167 Centro de Formação Em Ciências Agroflorestais, Universidade Federal Do Sul da Bahia, Ilhéus, Brazil; 168 Broward County Parks and Recreation, Oakland Park, FL USA; 169 Florida Atlantic University, Boca Raton, FL USA; 170 Field Museum of Natural History, Collections, Conservation, and Research, Chicago, USA; 171 The Nature Conservancy, 2424 Spruce St., Boulder, CO USA; 172 Ecology and Evolutionary Biology, University of Colorado, Boulder, CO USA; 173 Universidad Nacional de La Amazonía Peruana, Iquitos, Peru; 174 Facultad de Ciencias Forestales y Ambientales, Universidad de Los Andes, Mérida, Venezuela; 175 Deccan Regional Centre, Botanical Survey of India, Kendriya Sadan, Koti, 500001 Hyderabad, Telangana India; 176 Servicios Ecoysistemicos y Cambio Climatico (SECC) Fundación Con Vida & Corporación COL-TREE, Medellín, Colombia; 177 Embrapa Amazônia Ocidental, Manaus, AM Brazil; 178 CIMA Cordillera Azul, Av. Ricardo Palma 341 Of.302, Miraflores, Lima, Peru; 179 Iwokrama International Centre for Rainforest Conservation and Development, Georgetown, Guyana; 180 Department of Biology, University of Florence, Florence, Italy; 181 MUSE-Science Museum, Trento, Italy; 182 Department of Botany, Institute of Science, Banaras Hindu University, Varanasi, 221005 India; 183 Departamento de Biología, Universidad de La Serena, La Serena, Chile; 184 Instituto de Ecología y Biodiversidad (IEB), Santiago, Chile; 185 Centro de Ciências Biológicas E da Natureza, Laboratório de Botânica E Ecologia Vegetal, Universidade Federal Do Acre, Rio Branco, Brazil; 186 School of Biological Sciences, University of Aberdeen, Aberdeen, UK; 187 Wildlife Conservation Society, New York, NY USA; 188 CIFOR‑ICRAF, Yaoundé, Cameroon; 189 National Institute of Cartography, P.O Box 157, Yaoundé, Cameroon; 190 Florida Museum of Natural History and Department of Biology, University of Florida, Gainesville, USA; 191 School of Science and Engineering, James Cook University, Cairns, Australia; 192 Department of Botany and Plant Pathology, Oregon State University, Corvallis, OR 97331 USA; 193 Faculté Des Sciences Appliquées, Université de Mbujimayi, Mbujimayi, Democratic Republic of Congo; 194 Global Environment Facility (GEF), Washington, DC 20433 USA; 195 The Yale School of the Environment, New Haven, CT 06511 USA; 196 Museu Paraense Emílio Goeldi. Coordenação de Ciências da Terra E Ecologia, Belém, Pará, Brasil; 197 School of Geography, University of Nottingham, University Park, Nottingham, NG7 2RD UK; 198 Wildlife Conservation Society, 2300 Southern Boulevard, Bronx, NY 10460 USA; 199 Bioversity International, Rome, Italy; 200 Plant Ecology and Biogeochemistry Lab, Faculty of Sciences, Université Libre de Bruxelles, Bruxelles, Belgium; 201 Georg-August University Göttingen, Untere Karspüle 2, 37073 Göttingen, Germany; 202 Universidad Autónoma del Beni José Ballivián, Trinidad, Bolivia; 203 Division of Forestry and Natural Resources, West Virginia University, Morgantown, WV USA; 204 Department of Forest Management, Centre for Agricultural Research in Suriname (CELOS), Prof. Dr. Ir. J. Ruinardlaan, Paramaribo, Suriname; 205 Tropenbos International, Ede, The Netherlands

**Keywords:** Aboveground biomass, Climatic drivers, Structural attributes, Old-growth tropical forests, Structure-explicit pathway modelling, Pantropical, Climate sciences, Ecology, Ecology, Environmental sciences

## Abstract

Old-growth tropical forests store vast amounts of carbon in their aboveground biomass (AGB), yet the relative roles of abiotic factors such as climate, soil, and topography in governing its spatial distribution remain poorly understood. In particular, the degree to which climate acts on AGB through forest structure is still poorly quantified at the pantropical scale. Using a pantropical dataset of more than 2,000 old-growth forest plots and a structure-explicit framework, we assess how climate influences AGB through its effects on four structural attributes: basal area, mean diameter, stem density, and basal area-weighted wood density. We find that climate shapes AGB primarily through its effects on forest structure. However, structural attributes respond to climate in opposite directions, so climate’s net effect on AGB largely cancels out, and no clear climate-AGB relationship emerges across tropical regions. Moreover, only wood density responds consistently, decreasing with annual precipitation and increasing with precipitation seasonality, whereas all other attributes respond to climate differently from one region to another. This geographical variation further obscures any global climatic signal on AGB and points to the role of biogeographic history in shaping forest structure. Our findings highlight the central role of the climate-structure nexus in explaining AGB variation, and call for structure-explicit models to improve carbon stock predictions and inform climate adaptation strategies.

**Supplementary Information:**

The online version contains supplementary material available at https://doi.org/10.1038/s41598-026-61888-7.

## Main text

Covering 45% of Earth’s forested area, tropical forests store a large amount of carbon equivalent to about one-third of the carbon present in the atmosphere, with old-growth forests holding a large amount of it in their aboveground biomass (AGB)^1–6^. Moreover, they host over 50% of terrestrial species, with unparalleled biodiversity and endemism^7–11^. Preserving these carbon-rich and ecologically vital ecosystems is imperative, as they are heavily threatened by deforestation, forest degradation, defaunation, and climate change^12,13^.

Despite their well-recognized role in the global carbon budget, tropical forests remain the most uncertain component^1^. A major challenge in mapping and predicting tropical forest carbon stocks globally lies in our limited understanding of how abiotic factors influence the spatial distribution of AGB. Old-growth tropical forests, approaching structural equilibrium with their local environment, are well suited for investigating this question^14^. Yet, across studies that have explored spatial relationships between plot-level AGB in old-growth forests and environmental factors such as temperature, precipitation, topography, and soil properties, results vary widely in both magnitude and direction. For instance, it is not clear whether increases in annual precipitation are systematically associated with increases in biomass: some studies report a positive relationship in African and Southeast Asian forests^15,16^, whereas others in South America find a positive effect only in low-biomass forests (< 150 Mg ha^-1^)^17^, no significant link^18^, or a unimodal (Gaussian) pattern^19^. Such inconsistencies across regions and environmental factors remain difficult to explain^15–27^.

We argue that these inconsistencies arise because most previous studies focus on the overall effect of the environment on AGB, without resolving how forest structure and species composition vary along environmental gradients. Yet, forest AGB is an emergent property of multiple plot-level structural attributes, including basal area, mean diameter, stem density, and mean wood density (itself dependent on community species composition)^23,28,29^, each of which responds individually, and often in opposite directions, to the same environmental factor^18,21,22,24^. In South America, for instance, AGB variation arose from opposite responses of basal area and wood density along drought and fertility gradients^18^, and in Gabon precipitation had no detectable effect on AGB even though structural attributes shifted along the precipitation gradient^24^. Because these divergent responses produce a diversity of forest architectures and space-occupying strategies^30^, environment-AGB relationships often go undetected: not because the environment has no influence, but because opposite structural responses along environmental gradients can cancel out at the AGB level.

Therefore, in this study, we argue that understanding how environmental factors influence individual structural attributes is key to explaining the spatial distribution of forest AGB. We test the hypothesis that environmental factors influence forest AGB through opposite responses among forest structural attributes, using extensive, pantropical forest inventory data from 2,023 plots of old-growth tropical moist broadleaf forest. To our knowledge, no study has yet investigated this question for the entire tropical forest biome, and doing so may improve our understanding of tropical forest ecology and predictions of forest responses to global change.

**Fig. 1:**
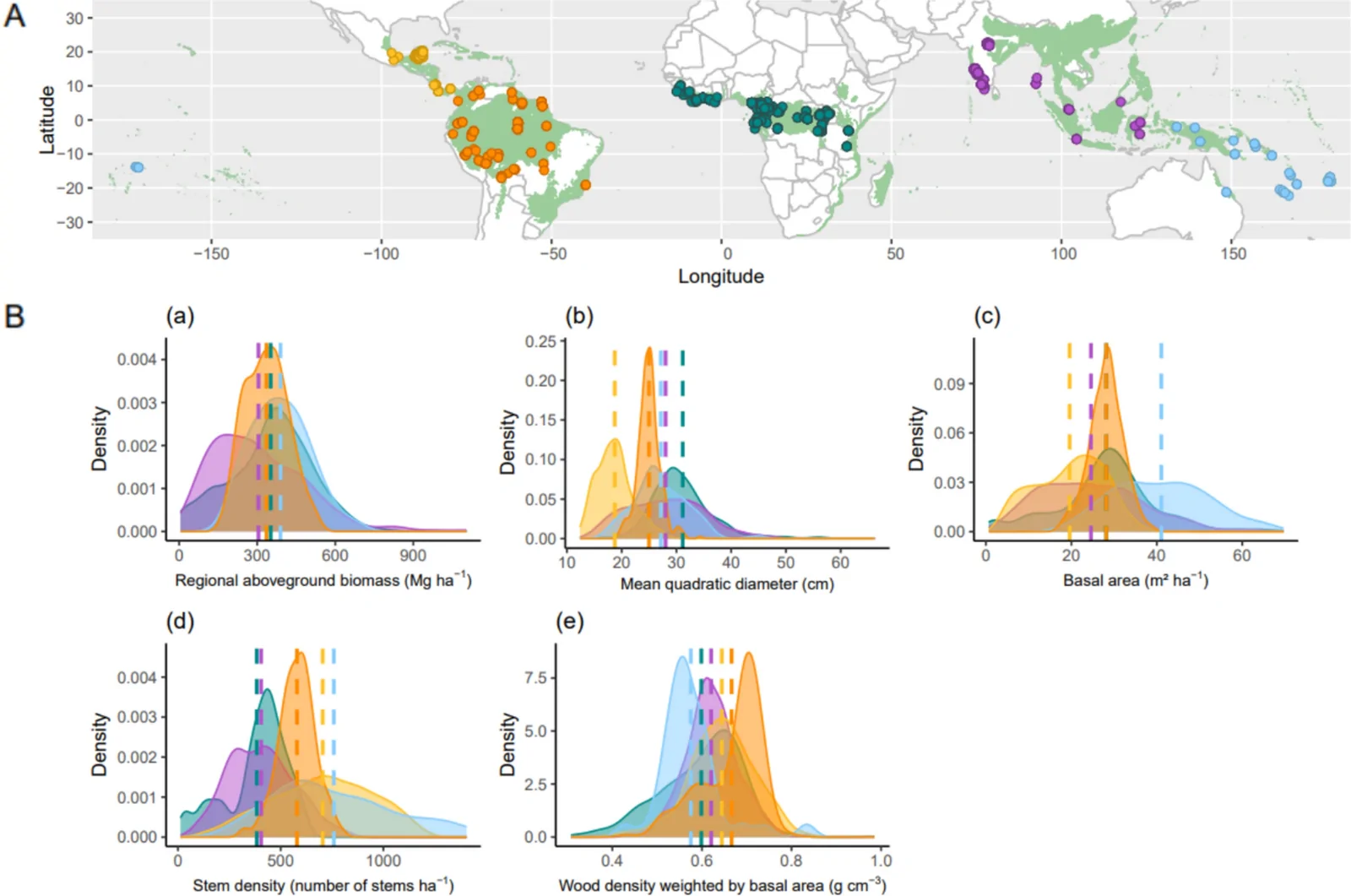
Variation in forest structure and aboveground biomass across tropical regions, including montane forests. (**A**) Locations of the 2,023 inventoried plots across five regions: 614 in Central America (yellow, including plots in southern Mexico), 357 in South America (orange), 714 in Africa (green), 267 in Asia (purple), and 71 in Oceania (light blue). Plot data were sourced from ForestPlots.net^35–37^, the Global Forest Biodiversity Initiative^38^ (GFBI, via Science-i web platform, https://www.gfbinitiative.org/), which includes long-term plot data from Panama^39–41^, AfriTRON (https://afritron.org/) and RAINFOR (https://rainfor.org/en/) networks, and from direct contributions by principal investigators (see Methods). Forest extent (shown in green) is based on the Tropical and Subtropical Moist Broadleaf Forests biome from the RESOLVE Ecoregions dataset^42^. (**B**) AGB and structural attribute distributions by region (same colors as shown on the map), shown as probability density functions. Dashed lines indicate the mean values. Results are shown for AGB estimated using tree height derived from the region-specific height-diameter (H–D) model of Feldpausch et al. (see Methods)^43^. Extended Data Fig. 1 shows the distribution of plots excluding montane forests, and Extended Data Figs. 2 and 3 show the AGB distribution estimated using the pantropical and continent-specific H–D models^43^.

**Fig. 2:**
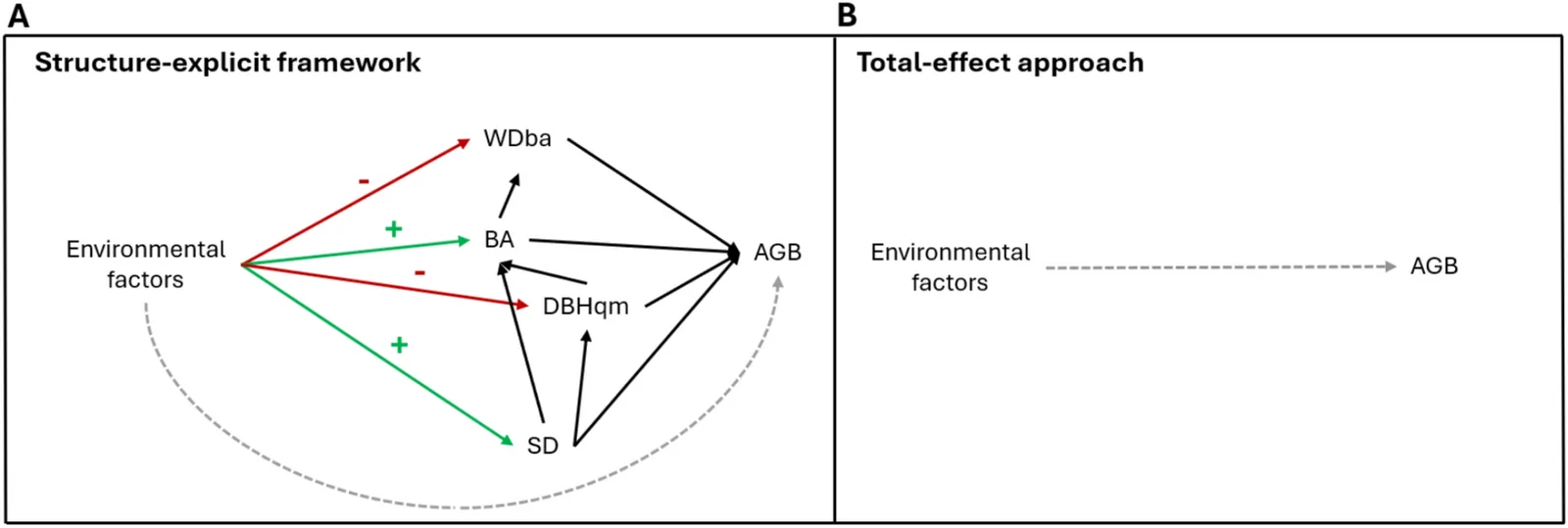
Structure-explicit framework vs. total-effect approach for investigating environmental influences on tropical forest aboveground biomass (AGB). Arrows indicate assumed pathway associations, with dashed arrows denoting effects expected to be weak. (**A**) Directed acyclic graphs depicting the indirect pathways through which selected environmental variables are hypothesized to influence AGB via opposite responses among forest structural attributes (see Methods). Colors illustrate opposite positive and negative effects. Structural attributes include basal area (BA), wood density weighted by basal area (WDba), quadratic mean diameter (DBHqm), and stem density (SD). In addition, residual direct pathways between environmental variables and AGB are assumed after accounting for variation in forest structural attributes along environmental gradients and are indicated by dashed arrows. (**B**) Common approaches in the literature used to assess the total environment–AGB relationship, without explicitly accounting for forest structural attributes and their opposite responses along environmental gradients. This corresponds to a total-effect analysis, in which both (residual) direct and indirect pathways remain open (see Methods).

We compiled our dataset across five regions (Fig. 1; see Methods): Central America (n = 614, including southern Mexico), South America (n = 357), Africa (n = 714), Asia (n = 267), and Oceania (n = 71), all selected from 19,473 candidate plots through stringent filtering to ensure consistent inventory protocols (Extended Data Table 1). We focused on old-growth forests, i.e., undisturbed forests or older secondary forests that have recovered old-growth structural values after past disturbances, so that the spatial variation in forest structure we observed reflected the environment rather than ongoing successional dynamics (see Methods)^14^. For each plot, we calculated plot-level AGB and four key structural attributes: basal area, quadratic mean diameter, stem density, and wood density weighted by basal area, standardized per hectare (Extended Data Table 2). We then used a structure-explicit pathway modelling framework (see Methods) to estimate the influence of environmental factors (i.e., mean annual temperature, total annual precipitation, precipitation seasonality, soil pH, sand content, soil organic carbon, and topographic slope) on AGB mediated through forest structural attributes (Fig. 2A). Climate-structure-AGB relationships assessed within this structure-explicit framework are hereafter referred to as indirect relationships. We also estimated the environment-AGB relationships without accounting for variation in forest structure along environmental gradients (hereafter, total relationships), as is common in the literature (Fig. 2B). We controlled for spatial autocorrelation in the residuals of each model^31–34^ and assessed the robustness of our conclusions through several sensitivity analyses, including the use of eight climate datasets and the inclusion of montane forests, which are most prevalent in Africa (see Methods and Extended Data Figs. 7–8). We expected to identify opposite responses of structural attributes to environmental drivers that, in turn, shape AGB (Fig. 2A), thereby providing deeper insight into environment-AGB relationships than total-effect analyses alone (Fig. 2B).

At the pantropical scale, modelling indirect effects of environmental factors on forest AGB while accounting for region-environment interactions showed that intercepts and slopes differed significantly between regions (Extended Data Table 4). Such strong regional dependence means that assuming a single pantropical relationship would overlook key mechanisms underlying tropical forest structure and AGB.

**Fig. 3:**
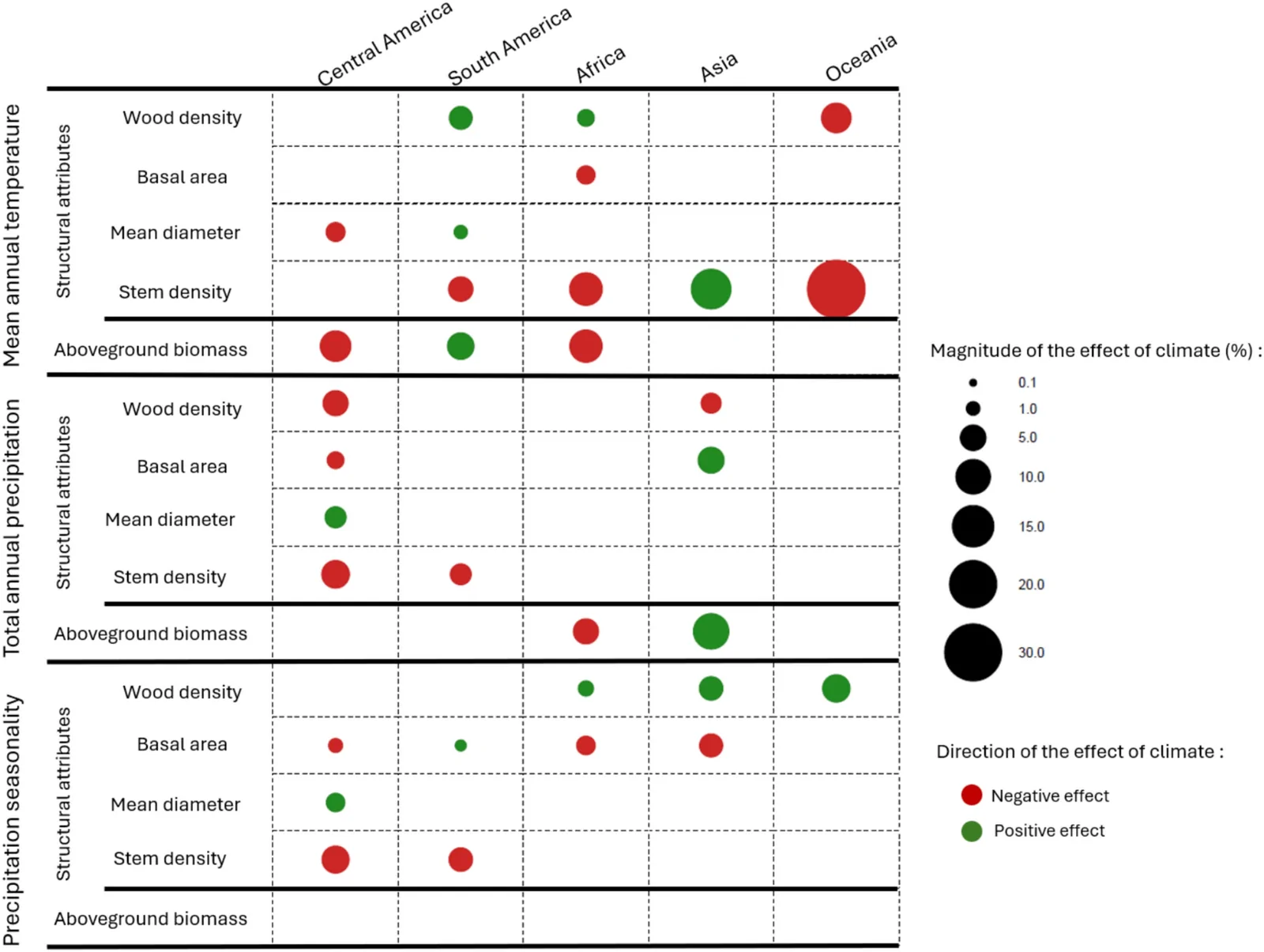
Indirect effects and total effects of climate on aboveground biomass (AGB) across tropical regions, excluding montane forests. Standardized coefficients of relationships between climatic variables and structural attributes, and of total relationships between climatic variables and AGB, as identified by the structure-explicit pathway modelling framework (see Methods). Columns correspond to the five regions studied. Coefficients are shown as colored circles, expressed as percentages relative to the mean of the response variable per standard deviation (%). Circle size reflects the magnitude of each effect, with values for the three climatic variables representing the average coefficients obtained across different climate data sources (see Methods). Green indicates positive effects, red indicates negative effects. Empty boxes indicate non-significant coefficients (p ≥ 0.05). Results are shown for AGB estimated using tree height derived from the pantropical height-diameter (H–D) model of Feldpausch et al. (see Methods)^43^. The complete set of results for indirect relationships between environment and AGB is presented in Extended Data Figs. 9–13. Coefficients in raw, standardized, and % units are reported in Extended Data Tables 5 and 7. RMSE and R^2^ values for both total-effect and indirect relationships are provided in Extended Data Table 9 and Extended Data Figs. 9–13. Bivariate relationships between each climatic variable (x-axis) and response variable (y-axis) are shown in Extended Data Figs. 18–24. The same patterns hold when using the continent- and region-specific H–D models of Feldpausch et al.^43^ to estimate AGB (Extended Data Tables 5 and 7), and when including montane forests (Extended Data Figs. 15–16 and Extended Data Tables 6, 8 and 10).

**Fig. 4:**
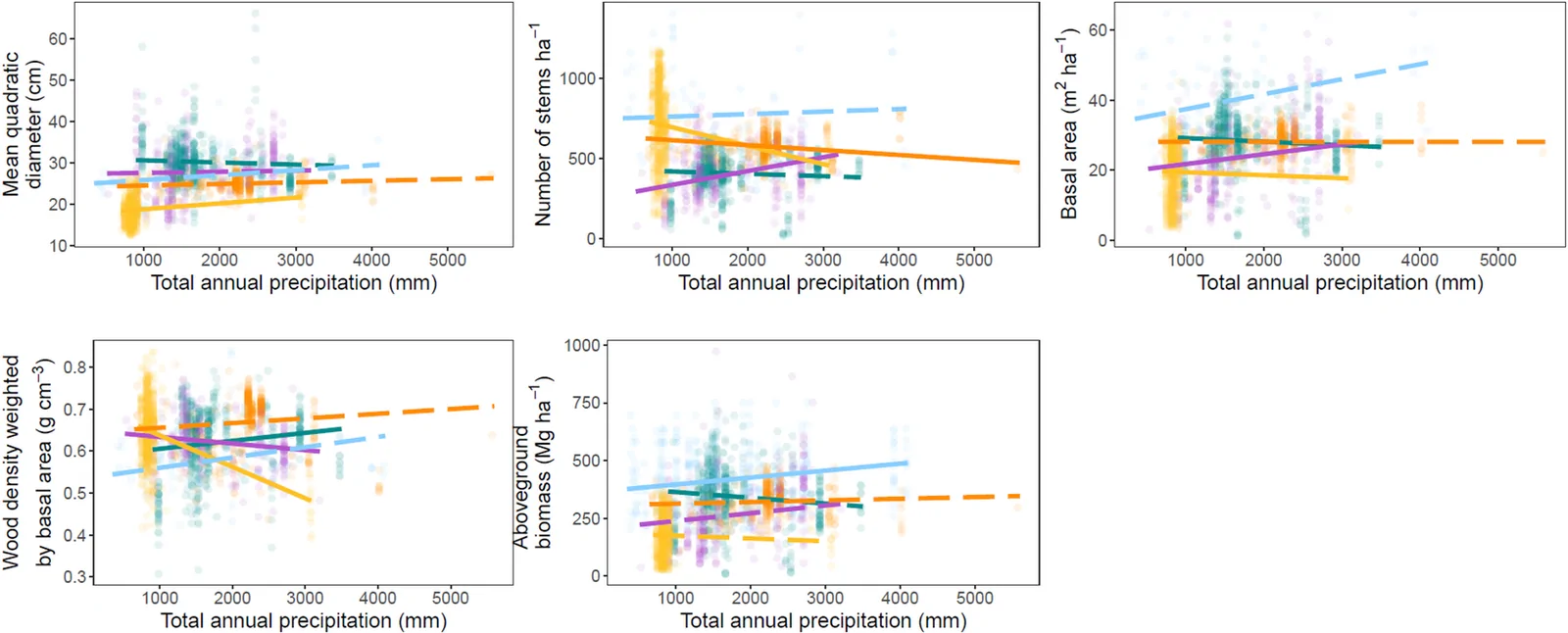
Indirect effects and total effects of total annual precipitation (TAP) on aboveground biomass (AGB) across tropical regions, excluding montane forests (MODIS + CHIRPS dataset). The figure shows the indirect bivariate relationships between TAP and forest structural attributes, and the total bivariate relationships between TAP and AGB, derived from the structure-explicit pathway modelling framework (see Methods). Solid lines indicate statistically significant relationships (p < 0.05), while dashed lines indicate non-significant relationships (p ≥ 0.05). Colors correspond to each region as shown on the map (Fig. 1). Results are shown for AGB estimated using tree height derived from the pantropical height-diameter (H–D) model of Feldpausch et al. (see Methods)^43^. See Extended Data Figs. 18–24 for all results across all climate data sources.

At the regional scale, the contrast between the two approaches was striking. The total-effect analysis detected little climatic influence on AGB, whereas the structure-explicit analysis revealed a strong one: across regions, structural attributes responded to climate in distinct and often opposite directions, giving rise to forest structural gradients that ultimately shaped forest AGB (Figs. 3, 4, Extended Data Figs. 9–13). Once these indirect effects were accounted for, residual direct climate-AGB relationships were weak and rarely significant (Extended Data Figs. 9–13). Relying on total effects alone therefore led to a substantial loss of insight into the climatic influence on AGB, in line with previous observations in South America, Africa, and Asia^15,16,18,24,44^. For example, mean annual temperature showed relationships with AGB through forest structural attributes across all five regions, but in only three regions when considering total climate-AGB effects (Figs. 3, 4 and Extended Data Table 7). Similarly, total annual precipitation and precipitation seasonality showed signals in three and five regions, respectively, as indirect relationships, versus two and zero as total relationships (Figs. 3, 4 and Extended Data Table 7). Together, these results support our central hypothesis: climate influences AGB primarily through its effects on forest structure, with structural attributes responding in opposite directions along climatic gradients (Fig. 2A). Disentangling climate effects on individual forest structural attributes therefore provides deeper insights into climate-AGB relationships than total-effect analyses alone (Fig. 2B). This pattern held whether or not montane forests were included in Asia and Africa, indicating that climate-related structural variation reflects broader ecological responses rather than being solely due to the contrast between montane and lowland forests (Extended Data Figs. 15–16 and Extended Data Tables 6 and 8). While results concerning topographic slope and soil variables were consistent with our central hypothesis that AGB is primarily shaped by environment-structure relationships (Extended Data Figs. 9–13 and Extended Data Table 7), their interpretation requires caution. Additional effects associated with topography and soil physical and chemical properties likely operate at finer spatial scales (< 10 m)^22^ and should be further explored at the local scale.

Furthermore, structural responses to climate varied markedly between regions (Fig. 3, Extended Data Figs. 18–24 and Extended Data Table 5). Among all structural attributes and potential pathways at the regional level, only wood density weighted by basal area showed, when statistically significant, consistent relationships with climatic factors (Fig. 3). Specifically, it tended to exhibit a negative relationship with annual precipitation and a positive relationship with precipitation seasonality. For all other structural attributes, the results were often inconsistent between regions: significant in some regions but not others, or divergent in direction (Fig. 3).

In Central America, precipitation strongly shaped forest structure. Both higher total annual precipitation (SD = 354 mm) and greater precipitation seasonality (SD = 4%) were associated with lower stem density (-42 and -40 stems ha^-1^ per SD; about -6% of the regional mean) but larger mean diameter (+ 2.3 and + 1.0 cm per SD; + 8% and + 3%). Higher total annual precipitation was additionally associated with lower wood density (-0.03 g cm^-3^ per SD, -5%) (Fig. 3 and Extended Data Table 5). These results corroborate and extend previous findings for the region^19^.

In South America, temperature appeared to be a key driver of forest structure. Warmer temperatures (SD = 1 °C) were associated with lower stem density (-26 stems ha^-1^ per SD, -5%) but larger mean diameter (+ 0.2 cm per SD, + 1%) and higher wood density (+ 0.03 g cm^-3^ per SD, + 4%) (Fig. 3 and Extended Data Table 5). These patterns are consistent with previous studies highlighting strong links between climate, elevation, and forest structure in the region^18,19,27^.

In Africa, both temperature and precipitation seasonality shaped forest structure. Warmer temperatures (SD = 1 °C) were associated with lower stem density (-35 stems ha^-1^ per SD, -9%) and lower basal area (-0.6 m^2^ ha^-1^ per SD, -2%) but higher wood density (+ 0.01 g cm^-3^ per SD, + 2%). Greater precipitation seasonality (SD = 16%) was also associated with lower basal area (-0.6 m^2^ ha^-1^ per SD, -2%) and higher wood density (+ 0.01 g cm^-3^ per SD, + 1%) (Fig. 3 and Extended Data Table 5). These responses remain underexplored, partly because Africa’s two wet and two dry seasons make it a unique case in the pantropics. Earlier work in Gabon reported a negative effect of annual precipitation on forest structure in old-growth lowland forests^24^, likely driven by excessive wet-season rainfall^16^.

In Asia, total annual precipitation (SD = 869 mm) and its seasonality (SD = 34%) were associated with opposite patterns in basal area and wood density: higher precipitation with higher basal area (+ 1 m^2^ ha^-1^ per SD, + 5%) and lower wood density (-0.02 g cm^-3^ per SD, -3%), and greater seasonality with lower basal area (-1 m^2^ ha^-1^ per SD, -4%) and higher wood density (+ 0.03 g cm^-3^ per SD, + 4%) (Fig. 3 and Extended Data Table 5). These trends align with intact lowland forests in Borneo^15^.

In Oceania, temperature appeared to strongly shape forest structure. While precipitation seasonality (SD = 18%) was related to wood density (+ 0.03 g cm^-3^ per SD, + 6%; Fig. 3 and Extended Data Table 5), in Australian forests they found an influence of precipitation seasonality on tree height-diameter allometry^45^. Additionally, cyclone activity, which covaries with temperature and increases with longitude, likely plays a major role in driving forest height and stem density^46^.

These results suggest that no single pantropical law can adequately describe the climate-structure relationships across tropical forests, and that the key bioclimatic drivers of forest structure differ between regions. Such regional differences may reflect divergent historical biogeographic processes, including glaciation, continental drift, and speciation, which have shaped distinct species pools and adaptive strategies in response to climate conditions^47,48^. Methodological factors may also contribute to these discrepancies, as linear models were preferred over more complex forms, and only the most influential climate variables were retained. However, tests using non-linear approaches and preliminary analyses of variable correlations indicate that this modelling choice does not substantially affect the results (Extended Data Fig. 25). Further work on the ecological and evolutionary mechanisms underlying regional variation in climate-structure relationships would improve our understanding of how climate influences forest structure.

Canopy height is a key structural attribute shaping AGB that responds to climate but could not be included here because most field inventories lack tree height measurements^43,45,48–50^. Yet, canopy height is known to covary with quadratic mean diameter and stem density. For instance, African forests are characterized by lower stem density and by larger-diameter, taller trees than Amazonian forests, which typically have higher stem density and smaller-diameter trees^16,45^. Therefore, the effect of the quadratic mean diameter on AGB estimated here should be interpreted as a total effect that partially operates through canopy height. Had canopy height been explicitly modelled, it would have added a further indirect climate-AGB pathway, likely reducing the residual direct effects currently observed (see Methods). The growing availability of airborne and spaceborne LiDAR data, providing canopy height estimates at large spatial scales, offers a promising avenue to explicitly incorporate height into such structure-explicit frameworks^51,52^, and would allow a finer decomposition of climate-structure-AGB pathways in future analyses.

In this study, we show that understanding the abiotic drivers of tropical forest AGB requires focusing on how individual structural attributes vary along climatic gradients within each region. This structure-explicit framework paves the way for a better understanding of how forest structural attributes vary spatially across the tropics and how they shape forest biomass variation. Under a space-for-time perspective, future climate change is likely to affect individual structural attributes in more consistent directions than AGB itself, potentially reducing uncertainty in carbon stock predictions. Yet, repeated long-term measurements on the same plots would be required to directly quantify the temporal dynamics of climate-structure relationships, a key next step for future research.

## Conclusion

This study demonstrates that understanding climate-biomass relationships in tropical forests requires explicitly resolving how climate drives individual forest structural attributes. We show that forest AGB arises from opposite structural responses along climatic gradients, making total climate-AGB relationships often undetectable. This pattern is consistent across tropical regions, despite differences in floristics, environment, and forest type, and remains robust across multiple climate datasets. We further reveal that the climate-structure relationships vary substantially between regions, challenging the notion of a single pantropical relationship. Explicitly accounting for region-specific climate-structure-AGB relationships may therefore improve our understanding of forest AGB variation and potentially contribute to reducing uncertainty in carbon stock predictions under future environmental change, strengthening the basis for forest conservation, management, and climate mitigation.

## Methods

### Plot network

We compiled inventories from 2,023 plots of old-growth tropical moist broadleaf forests (ranging from 0.25 to 9 ha), spanning five regions (Figs. 1 and Extended Data Figs. 1–3): Central America (n = 614, including plots in southern Mexico), South America (n = 357), Africa (n = 714), Asia (n = 267), and Oceania (n = 71). Total annual precipitation ranges from 780 mm to 5,992 mm and mean annual temperature ranges between 12 °C and 27 °C (Extended Data Figs. 4–5). The plots span a wide altitudinal gradient (4 to 3,021 m above sea level), with montane forests (⩾1,000 m) representing 22% of the plots in Africa, 6% in Asia, and less than 3% in all other regions. These plots were rigorously selected from over 19,473 initial plots located between 23°N and 23°S, with forest inventories provided by ForestPlots.net (https://forestplots.net/)^35–37^, the Global Forest Biodiversity Initiative (GFBI, in collaboration with the Science-i web platform, https://www.gfbinitiative.org/)^38^, which includes long-term plot data from Panama^39–41^ , AfriTRON (https://afritron.org/) and RAINFOR (https://rainfor.org/en/) networks, and by direct contributions from principal investigators of tropical field plots. The original forest inventories included tree-level measurements of diameter at breast height (dbh, measured at 130 cm from the ground or 50 cm above buttresses), taxonomic identification (to genus or species level), and plot-level information such as geographic coordinates, plot size, and census year.

Plots were rigorously selected through a stringent filtering process based on the following criteria (Extended Data Table 1). Within each plot, we retained trees with a dbh greater than or equal to 10 cm. Plots were removed when 5% or more of the trees had missing dbh measurements. We selected plots with a size greater than or equal to 0.25 hectares (plot size range: 0.25–9 ha, Extended Data Fig. 26). Previous studies have shown that 0.25 ha is the minimum plot size required to minimize intra-sample variance in basal area and aboveground biomass (AGB) estimates^53,54^. We retained only plots where at least 90% of the trees were identified at the genus level^27^. We ensured that plots were located within tropical moist broadleaf forests based on field data and the RESOLVE Ecoregions dataset (2017) ^42^. During the calculation of AGB and stem density (see next section), we observed extreme values. Plot size errors were identified in some African plots by checking for inconsistencies in plot properties, using data from the ForestPlots network (https://www.forestplots.net/)^35–37^. In addition, as the most extreme values in our dataset could not all be individually verified, and may represent either encoding or measurement errors or ecologically real and exceptional forest stands, to focus on the main ecological patterns driving the state of old-growth tropical forests, we removed the upper and lower 3% of values for stem density and the upper and lower 2% for AGB. Based on detailed field-based information, we ensured that our dataset focuses on old-growth forests, explicitly excluding plots with evidence of recent disturbance (e.g., logged, burned, disturbed, early successional, fallow, pioneer, or young to intermediate secondary forests (< 50 years)). The inventory period spans from 1974 to 2023. When multiple censuses were available for a given plot, we retained data from the most recent inventory. Additional cleaning steps and the resulting number of plots at each step are summarized in Extended Data Table 1, highlighting the continued need for the standardization and curation of global forest inventory data.

### Forest structural attributes and aboveground biomass

We calculated four forest structural attributes at the 1-ha plot level^17,23^: the basal area (BA), the mean quadratic diameter (DBHqm), the community wood density weighted by basal area (WDba), and the stem density (SD). Their calculations and definitions are summarized in Extended Data Table 2. To obtain the wood density of each tree, we first checked for typing errors in taxonomic identifications using the Taxonomic Name Resolution Service (TNRS)^55^ via the Taxosaurus interface (http://taxosaurus.org/). Then, we associated a genus- or species-level averaged wood density value with each taxon using the global wood density database (GWDD)^56,57^. For unmatched taxa or unidentified trees, we assigned the average wood density of the plot to which each tree belonged. This was implemented using the *getWoodDensity* function from the BIOMASS R package^58^. In the GFBI dataset, 72% of trees were identified at the species level and 22% at the genus level. In the ForestPlots dataset, 56% of trees were identified at the species level and 35% at the genus level. For plots contributed directly by principal investigators, 48% were identified at the species level and 37% at the genus level. We did not include plot-level canopy height as a forest structural attribute, because most field inventories lacked tree height measurements.

We calculated forest AGB as the sum of the AGB of all individual trees with dbh ⩾ 10 cm, standardized to a per-hectare value (Extended Data Table 2). We estimated the AGB of each tree using allometric Eq. 4 from Chave et al. (Extended Data Table 2)^49^, which combines tree dbh, wood density, and height. Tree height was estimated using the three tree-level H–D models from Feldpausch et al.^43^: the pantropical, continent- or region-specific H–D models (Extended Data Table 2). For Central America, AGB was calculated exclusively using the pantropical H–D model, as no specific region- or continent-specific H–D model exists for this region. This was implemented using the *retrieveH and computeAGB* functions from the BIOMASS R package^58^.

### Environmental variables

As it has been shown that our capacity to predict forest species distributions varies depending on the climate data source^59^, we tested several publicly available climate datasets in our models (see next section). With this aim, we extracted near- or global-scale temperature and precipitation raster data from two sources based on weather station data (i.e., WorldClim^60^, and CRU TS^61^) and from six sources based on remote sensing data (i.e., CHELSA^62^, ERA5-Land^63^, TerraClimate^64^, IMERG^65^, CHIRPS^66^, and MODIS^67^). All climatic data were retrieved and processed using Google Earth Engine^68^ and are publicly available, except for CHELSA data, which are available at https://chelsa-climate.org, WorldClim at https://www.worldclim.org, and CRU at https://crudata.uea.ac.uk/cru/data/hrg (Extended Data Table 3). In addition, we extracted topographic data (slope and elevation) at a 30 m spatial resolution from the Shuttle Radar Topography Mission (SRTM)^69^ and soil data (clay content, sand content, organic carbon content, water content, bulk density, and pH) averaged across three depths (0, 10, and 30 cm) at a 250 m resolution from the Global Soil Information System (SoilGrids250m)^70^, also accessed and processed through Google Earth Engine (Extended Data Table 3)^68^.

We reduced the initial set of available environmental variables (19 bioclimatic variables derived from the WorldClim dataset^60^, two topographic, and six soil variables), prior to running our models, to ensure limited collinearity while retaining complementary climatic, topographic, and edaphic elements (see below). To do so, we first examined the pairwise correlations between the variables and excluded one variable from each pair with correlation coefficients greater than or equal to 0.7. We then conducted a principal component analysis (PCA) based on a correlation matrix and applied a clustering analysis (i.e., Ward’s minimum variance clustering method) on a Euclidean distance matrix derived from the coordinates of the environmental variables on the first four PCA axes (describing 73% of the total PCA inertia)^71^ (Extended Data Fig. 6). This was carried out using the *vegan* and *stats* R packages^72^. Based on these clusters, we finally selected three bioclimatic variables to characterize the climate, prioritizing those with direct relevance to forest ecology and processes (Extended Data Fig. 6): annual mean temperature, total annual precipitation, and precipitation seasonality (Extended Data Figs. 4–5). For each climate data source, the three bioclimatic variables were calculated following the WorldClim methodology, averaged over the 30-year period 1981–2010, and upscaled to a 0.5-degree resolution, primarily using Google Earth Engine^68^. We selected topographic slope, sand content, organic carbon content, and pH to capture key physical and biochemical properties of soils (Extended Data Figs. 4–5). This three-step procedure allowed us to select a limited set of environmental predictors expected to be ecologically relevant, and that we assume to be complementary enough to allow approximation of model slope coefficients of these predictors as relative and comparable influences on the response variable (see next section). The ecological inferences from the fitted models are conditional on these assumptions.

### Statistical analyses

#### Structure-explicit pathway modelling framework

To test hypothesized pathways linking the selected environmental variables (see previous section), forest structural attributes, and AGB, we used a structural causal modelling framework^73,74^.

We first defined a causal diagram of the studied system based on ecological reasoning using directed acyclic graphs (DAGs; Fig. 2A), in which arrows flow in a single direction and cannot cycle back, thereby representing hypothesized directional relationships between variables. We assumed that each environmental variable could potentially influence each structural attribute. We also assumed that each structural attribute could contribute to AGB, given dependencies among them. More specifically, we hypothesized that both stem density and quadratic mean diameter contribute to basal area, while stem density also influences quadratic mean diameter due to resource competition (higher stem density reduces quadratic mean diameter, in turn influencing basal area). In addition, we hypothesized that the effect of the environment on wood density weighted by basal area is partly explained by the balance between stem density and mean quadratic diameter. These assumptions define the indirect pathways through which environmental variables are hypothesized to influence AGB via forest structural attributes. We further assumed direct pathways between environmental variables and AGB after accounting for forest structural attributes, hereafter referred to as “residual direct” pathways. We do not postulate a priori the existence of a clear ecological mechanism underlying these potential residual direct effects. Rather, these pathways are included to capture potential residual effects arising for two reasons: (i) non-linear environmental influences on AGB that are not captured by indirect pathways through structural attributes, and (ii) effects mediated by structural variables not explicitly included in the analysis, such as canopy height, which itself exhibits a non-linear relationship with quadratic mean diameter. Nevertheless, residual direct effects are expected to be weaker than indirect effects, which will be indicated by the relative importance of (residual) direct versus indirect causal paths linking the environmental factors to AGB.

For each pair of outcome (i.e., response variable; e.g., AGB) and predictor of interest, that is, a variable for which we sought to estimate a hypothesized causal effect (e.g., temperature), we then applied a graphical criterion (the backdoor criterion) to the DAG^74^. This approach identifies a minimal set of control variables, if any exists, to adjust for, in order to block hypothesized non-causal paths of association between the predictor of interest and the outcome, while keeping hypothesized causal paths of association open between the two variables. This, in turn, ensures causal inference is possible from the estimated coefficient of the predictor of interest, assuming the structure of the DAG is correct^73,74^. It is worth emphasising that different causal queries generally lead to the backdoor criterion identifying different, and often incompatible, sets of control variables necessary for causal inference, hence requiring multiple models to be run for different questions (see below)^73–75^.

The ‘backdoor criterion’ yields different results depending on whether one aims to investigate total, (residual) direct, or indirect causal effects (see Eq. (1)-(12)). For the indirect causal pathways, we applied the backdoor criterion sequentially: first to assess the direct effects of environmental variables on each structural attribute, and then to evaluate the direct effects of each structural attribute on AGB, defining appropriate sets of control variables for each causal question. For the residual direct causal pathways, we focused on the direct effects of environmental variables on AGB when applying the criterion and defining related sets of control variables.

We also tested the total causal effects (sum of (residual) direct and all indirect causal effects) of environmental variables and of structural attributes on AGB. The total effect of environmental variables on AGB here corresponds to their influence without conditioning on structural attributes and thus keeps both (residual) direct and indirect causal pathways open (see Eq. (12)), consistent with common approaches used to assess the environment–AGB link in the literature (Fig. 2B).

Indirect, (residual) direct and total effects of the predictors of interest on the different response variables were tested using multiple linear regressions (MLRs) (hereafter referred to as “models”), with two-tailed tests applied in all analyses. All predictors were standardized to a mean of zero and unit standard deviation before running the models, to allow straightforward comparison of coefficients.

#### Models for indirect and residual direct effects

1. WDba = β₀ + β₁·*Env* + β₂·BA + ε
2. BA = β₀ + β₁·*Env* + β₂· DBHqm + β₃·SD + ε
3. DBHqm = β₀ + β₁·*Env* + β₂·SD + ε
4. SD = β₀ + β₁·*Env* + ε
5. AGB = β₀ + β₁·BA **+** ** β₂· DBHqm + β₃·SD + β₄· WDba** + β₅·*Env* + ε
6. AGB = β₀ + β₁· WDba + ** β₂·BA + β₃·*Env***  + ε
7. AGB = β₀ + β₁· DBHqm + β₂·SD +  ** β₃·BA + β₄·*Env***  + ε

#### Models for total effects

(8) AGB = β₀ + β₁· WDba + ** β₂·BA + β₃·*Env***  + ε
(9) AGB = β₀ + β₁·BA +  ** β₂· DBHqm + β₃·SD + β₄·.*Env*** + ε
(10) AGB = β₀ + β₁·SD +  ** β₂.*Env*** + ε
(11) AGB = β₀ + β₁· DBHqm +** β₂· .SD + β₃·.*Env*** + ε
(12) AGB = β₁·*Env* + ε

*Env* denotes the set of environmental variables included in all models. These include total annual precipitation, mean annual temperature, precipitation seasonality, topographic slope, soil sand content, soil pH, and soil organic carbon content. β₀ is the model intercept, β₁, β₂, … are estimated standardized coefficients for each predictor, ε is the residual error term. WDba: wood density weighted by basal area; BA: basal area; DBHqm: quadratic mean diameter; SD: stem density; AGB: aboveground biomass. Variables shown in bold correspond to the covariates to be conditioned on, to close non-causal paths and allow a causal interpretation of the slope of the predictor of interest.

By explicitly mapping the system through a DAG, one is required to consider all potential confounding factors surrounding the relationship of interest. The backdoor criterion then identifies which variables must be conditioned on to block spurious associations while keeping the pathway of interest open, thereby allowing directional inference with substantially reduced risk of confounding bias. However, as noted above, such causal inference is only valid to the extent that the structure of the DAG is correct^73,74^, an assumption we acknowledge cannot be fully guaranteed in a complex ecological system where not all relevant drivers and mediators can be captured. The estimated coefficients of the relationships tested should therefore be interpreted as pathway-based associations rather than as fully demonstrated causal effects. Accordingly, throughout the main text, we avoid strong causal language, and terms such as ‘effect’, ‘relationship’ or ‘influence’ should be understood as describing observed associations within the hypothesized pathway framework rather than as fully demonstrated causal relationships. In addition, we accounted for spatial autocorrelation in the residuals of each model using an advanced spatially constrained ordination method (Moran’s eigenvector maps, MEMs^31–33^), that is, a key step to avoid type I error-rate inflation and biased association estimates between predictors and response variables^34^. To do so, we first created a list of candidate graph-based spatial weighting matrices (SWMs) from the spatial coordinates of the forest plots, to test for spatial autocorrelation in the models’ residuals. If any significant spatial autocorrelation was detected, we optimized the selection of the smallest subset of spatial eigenvectors (or MEMs) necessary to account for all residual spatial autocorrelation, following Bauman et al.^31,32^. This was achieved using the *listw.candidates* and *listw.select* functions from the *adespatial* R package^32^ . The selected subset of MEMs was then incorporated alongside the environmental predictors, and the models were rerun, reinforcing the robustness of the tested relationships with environmental factors. Models were first fitted at the pantropical scale, including an interaction term with region using deviation coding (contrasts centred on the global mean) and using the CHELSA datasets. Then, at the regional scale, all models were fitted separately for each of the five regions (Central America, South America, Africa, Asia, and Oceania). To assess the sensitivity of model outcomes to the choice of climate data source, we ran the regional models while successively replacing the climate datasets: WorldClim, CRU TS, CHELSA, ERA5-Land, TerraClimate, MODIS + IMERG, and MODIS + CHIRPS. Our results showed that the climate dataset significantly influenced the estimated parameters in our models. In some cases, the slope of the relationship, and therefore the direction of the climate effect, varied across datasets, leading to opposite trends. In others, the direction was consistent but only for a minority of the climate sources, while the remaining datasets showed no significant relationship. Only a subset of relationships exhibited stable, significant slopes across all climate datasets (Extended Data Figs. 7–8). These results stressed the importance of climate data source selection in our understanding of the influence of climate on structural attributes and AGB. To ensure robust conclusions, for each relationship tested in our models (1)-(5) (at the regional scale), between the three bioclimatic variables and either structural attributes or AGB, we retained and interpreted ecologically only those relationships for which the majority of climate datasets (i.e., more than 3 out of 7) agree on both the significance and the direction of the coefficient (45% of cases for structural attributes versus 30.8% for AGB, excluding montane forests; Extended Data Figs. 7–8). For these relationships, the coefficients reported in the results represent mean values across all climate datasets. Relationships not meeting this criterion are considered non-significant.

Montane forests likely exhibit distinct structural and functional characteristics, shaped by specific, and sometimes more extreme, environmental conditions, making them a unique ecological system. Studying montane and lowland forests together could artificially accentuate climatic gradients, particularly for temperature, and potentially inflate the apparent influence of climate on structural attributes or AGB, while in fact reflecting a contrast between two distinct forest types rather than a continuous climate effect. To test whether the relationships observed between climate and forest structure or AGB were driven by the inclusion of montane forests, we conducted sensitivity analyses including and excluding montane plots from our regional models. These analyses were performed for Africa and Asia, where montane forests account for 22% and 6% of the dataset, respectively. For the other regions, where montane forests account for less than 3% of the plots, we retained only the models based on lowland forests.

Substantial differences in forest structure are generally observed between early- and late-successional forests^24,76,77^. Most tropical forests recover their structural properties within approximately 60 years after disturbance, and their AGB within approximately 120 years, converging towards old-growth values^14^. Given the selection criteria applied to our dataset, most of our forests are expected to have reached this structural asymptote, suggesting that the forest structural patterns we observe primarily reflect environmental conditions rather than ongoing successional dynamics. Nevertheless, some variation in successional stage may remain, potentially leading to incorrect inferences about the relationships between environmental factors and structural attributes. To address this concern, we evaluated whether controlling for successional stage could alter our main conclusions.

We used the percentage of pioneer species as a proxy for successional stage, reflecting the disturbance history of the plot: high percentages may indicate early successional stages following disturbance (natural or anthropogenic), while low percentages suggest more advanced stages. In our view, this provides a more robust measure than large-scale global disturbance datasets. The percentage of pioneer species was derived from the CoForTraits database (https://doi.org/10.18167/DVN1/Y2BIZK), which compiles trait data for Central African tree species.

Including this variable in the African models, the only region for which these data were available, did not alter our main conclusion that environment influences AGB indirectly through its influence on forest structural attributes (Extended Data Figs. 11, 14, 15 and 17). While such data are not available for other regions, the results provide reassurance that our conclusions are not driven by variation in forest maturity across tropical regions.

H–D models used to estimate tree height and then forest AGB exist from regional to pantropical scales^43^, but they come with inherent limitations, with each model having its own specific constraints. For instance, regional H–D models are generally more accurate but are calibrated for specific regions and influenced by varying environmental conditions, making it difficult to separate the true influences of environmental variables from model-induced biases. Therefore, we tested our models (at pantropical and regional scales) with AGB estimated using three alternative H–D models of Feldpausch et al.^43^ and found that the results showed similar patterns (Extended Data Tables 4–10), confirming that our main conclusions are robust to the choice of H–D model used to estimate AGB. For this reason, and because of the potential biases induced by region-specific H–D models, the interpretation of the relationships between environmental variables and AGB in the main text is based on AGB estimated with the pantropical H–D model.

Finally, as previously mentioned, the upper and lower 3% of stem density values and the upper and lower 2% of AGB values were removed from the dataset to focus on the main ecological patterns driving the state of old-growth tropical forests. To assess whether this trimming procedure could bias our main conclusions, we performed a sensitivity analysis in which all plots were retained without any trimming. The proportion of plots added relative to the main analysis was as follows: Central America (11.7%), South America (26.8%), Africa (5.1%), Asia (11.5%) and Oceania (9.9%). Results of this sensitivity analysis are presented in Extended Data Figs. 27–28 and show that our main conclusions remain unchanged: environmental factors exert strong indirect effects on AGB through opposite responses among structural attributes, while total environment–AGB relationships remain largely undetectable.

#### Complementary non-linear analyses

Additionally, we tested for potential non-linear (specifically quadratic) relationships between environmental variables and structural attributes at the pantropical scale. For each structural attribute, we examined its relationship with each bioclimatic variable (mean annual temperature, total annual precipitation, and precipitation seasonality) using simple linear regressions that included a quadratic term.

### Relationships between structural attributes and AGB

The relationships between structural attributes and AGB are not discussed in detail as they are not the primary focus of our study, which aims to demonstrate that understanding the influence of environment on AGB requires first understanding its influence on each structural attribute. As expected from previous studies^23,28^, forest AGB increased with all structural attributes across regions (Extended Data Figs. 9–13). Wood density showed a consistently positive association with AGB, and both quadratic mean diameter and stem density also contributed positively, mainly by enhancing basal area (Extended Data Figs. 9–13). A small negative association between quadratic mean diameter and AGB likely reflects low-biomass stands dominated by a few large trees, a pattern observed only in South America and Africa. The interaction observed between stem density and mean diameter defines a structural gradient, from sparsely stocked forests with large-diameter trees and lower basal area to denser stands characterized by smaller trees and higher basal area (Extended Data Figs. 10–13). In addition, the negative association between stem density and AGB suggests that in very dense forests, AGB tends to decrease, likely due to competition for resources and space (Extended Data Figs. 9–12). Altogether, although stem density plays a meaningful role, its total contribution to AGB is lower compared to basal area or wood density (Extended Data Table 7).

## Supplementary Information


Supplementary Information 1.
Supplementary Information 2.


## Data Availability

Tree-by-tree data from ForestPlots.net and GFBI are not publicly available due to data-sharing agreements. Raw forest inventory data are commonly subject to a wide array of confidentiality clauses in regard to open access policies. Despite recent efforts to make some of these data fully open^78,79^, some governments and private data owners, especially those from developing countries, have decided to keep their data confidential. This decision is based on well-founded arguments to protect certain trees or forests (because of their large size or protected taxonomic status) from illegal logging or trespassing and to protect landowners’ privacy against the misuse of plot information such as the geographic coordinates. Researchers interested in accessing the tree-by-tree data may submit a formal data request to ForestPlots.net and to the Global Forest Biodiversity Initiative (GFBI) (via Science-i.org). Requests are reviewed on a case-by-case basis and are subject to approval by the original data contributors. However, a summary dataset containing plot-level data (without geographic coordinates and without plot IDs), together with the code used to reproduce the analyses, is publicly available at https://doi.org/10.5281/zenodo.21169203 (see LICENSE for full terms)^80^. If you encounter issues reproducing the analyses using this code, or have questions about collaboration, please contact the corresponding author: Pauline Depoortere (depoort.p@gmail.com). Data from Panama used in this study are publicly available via permanent archives: Barro Colorado 50-ha plot: https://doi.org/10.15146/5xcp-0d46, BCI plot taxonomy: https://doi.org/10.15146/R3FH61, Panama 65 plot network: https://doi.org/10.15146/mdpr-pm59. French NFI campaigns (Raw Data) 2005 and following annual surveys were downloaded from https://inventaire-forestier.ign.fr/spip.php?rubrique159, site accessed on December 13th, 2015. All climate data were retrieved (https://earthengine.google.com), except for CHELSA data which are publicly available at https://chelsa-climate.org, WorldClim at https://www.worldclim.org, and CRU at https://crudata.uea.ac.uk/cru/data/hrg. All data were processed using Google Earth Engine. In addition, topographic data (elevation and slope) at 30 m resolution from the Shuttle Radar Topography Mission (SRTM) and soil data (clay content, sand content, organic carbon content, water content, bulk density, and pH) at 250 m resolution from the SoilGrids250m system were also accessed and processed through Google Earth Engine. All references and download links for the environmental datasets used in this study are provided in the Methods section and Extended Data Table 3.
