# Supplementary material for: Region-specific forest structure pathways reveal climate effects on old-growth tropical forest biomass
Source: Sci Rep. 2026 Sep 1;16:27381. doi: 10.1038/s41598-026-61888-7 (PMC13534615; doi:10.1038/s41598-026-61888-7)
Supplement: Supplementary file 1 [file 41598_2026_61888_MOESM1_ESM.pdf]

Title. Region-specific forest structure pathways reveal climate effects on old-growth tropical forest biomass

Author list

Pauline Depoortere[1*], David Bauman[2,3], Adeline Fayolle[4,5], Oliver Phillips[6], Lalasia Bialic-Murphy[7], Constantin M. Zohner[8,9], Arthur Vander Linden[1], Antoine Plumacker[1], Hugo de Lame[1], Jingjing Liang[10], Peter B. Reich[11,12], Javier G. P. Gamarra[13], Giorgio Alberti[14,15], Bruno Hérault[4,16], Nicolas Picard[17], Sergio de-Miguel[18,19], Gert-Jan Nabuurs[20], Thomas W. Crowther[21,9], Alain Paquette[22], Hans Pretzsch[23,24], Susan K. Wiser[25], Mo Zhou[10], Han Y. H. Chen[26,27,11], Hans Verbeeck[28], Charles De Cannière[29], Martin J. P. Sullivan[30], Lourens Poorter[31], Quentin Ponette[32], Marijn Bauters[28], Vincent Deblauwe[33,34], Gunnar Keppel[35], Francis Q. Brearley[30], Irié Casimir Zo-Bi[36], Tiodionwa Abdoulaye Ouattara[36], Michael J. Lawes[37,38], Frans Bongers[31], Jorge A. Meave[39], Mark Schulze[40], Michael Lyonga Ngoh[41], Hans ter Steege[42,43], Jürgen Homeier[44,45,46], Akomian Fortuné Azihou[47], Liam Trethowan[48], J. W. Ferry Slik[49], Géraldine Derroire[4,50,51], Damien Bonal[52], William F. Laurance[53], Andreas Hemp[54], Agustinus Murdjoko[55], Jesús Aguirre-Gutiérrez[56,57,58], Endang Kintamani[59], Lee White[60,61,62], Simon L. Lewis[6,63], Andrew Hector[64], Christian Amani[65], Ana Andrade[66], Albert Angbonga[67], Luiz Aragão[68,69], Ayushi Kurian[70,71], Félix Allah Barem[72], Angelica M. Almeyda Zambrano[73], Esteban Alvarez-Davila[74], Patricia Alvarez-Loayza[75], Elisée Amstrong[76], Alejandro Araujo-Murakami[77], Luzmila Arroyo[77], Timothy R. Baker[6], Ousmane Bako[78], Olaf Bánki[79], Christopher Baraloto[80], Nicolas Barbier[2], Jorcely Gonçalves Barroso[81], Patrick Bassama[78], Hans Beeckman[82], Nithanel Mikael Hendrik Benu[83], Hervé Bihiya[78], Philippe Birnbaum[2], Robert Bitariho[84], Pascal Boeckx[85], Kelly Boekee[86], Jan Bogaert[1], Katrin Böhning-Gease[87,88], Faustin Boyemba[89], Hilândia Brandão da Cunha[66], Roel Brienen[6], Jaime Briseño-Reyes[90], Eben N. Broadbent[73], Jose Luis Camargo[66], Benoît Cassart[32], Roberto Cazzolla Gatti[91], Jérôme Chave[92], George Chuyong[93], David B. Clark[94], Connie Clark[95], Alessio Collalti[96], Murray Collins[97], Fernando Cornejo Valverde[98], Jose Javier Corral-Rivas[90], Pierre Couteron[2], Aida Cuni-Sanchez[99,100], Lôla da Costa[101], Jose Daniel Soto[77], Ashaq Ahmad Dar[102,103], Javid Ahmad Dar[104,105], Gilles Dauby[2], Thales de Haulleville[28,1], Anthony Di Fiore[106,107], John Diisi[108], Marie-Noel Djuikouo K[109], Jiri Dolezal[110,111], Narcisse Dongmo[78], Jean-Louis Doucet[1], Vincent Droissart[2], Julien Engel[2,112], Nestor Laurier Engone Obiang[113], Santiago Espinosa[114,115], Teresa Eyre[116], Sophie Fauset[117], Ted R. Feldpausch[69], Markus Fischer[118], Krisna Gajapersad[119], Deden Girmansyah[120], Emanuel Gloor[6], Ieda Gomes do Amaral[66], Christelle Flore Gonmadje[121], Elisa Grieco[96], James Grogan[122], Richard Habonayo[123], David J. Harris[124], Eurídice Honorio Coronado[125], Isau Huamantupa-Chuquimaco[126], Wannes Hubau[82,127], Patrick A. Jansen[128,86,129], Kathryn J. Jeffery[130,2], Blaise Jumbam[131], Naveen Babu Kanda[132,133,102], Narcisse Kamdem[76], Emmanuel Kasongo Yakusu[134,82,127], John Katembo[135], Charles Kayijamahe[136], Elizabeth Kearsley[28,137], David Kenfack[128], Mohammed Latif Khan[138], Timothy Killeen[77], Subashree Kothandaraman[104,105], Ronald Kougue[78], Amit Kumar[139], Relawan Kuswandi[140], Nicolas Labrière[141], David Lasso[142], Susan Laurance[53], Miguel Leal[143], Hugo Leblanc[2], Moses Libalah[76], Janvier Lisingo[144], Yadvinder Malhi[57,58], Beatriz S. Marimon[145], Ben Hur Marimon Junior[145], Andrew R. Marshall[146,147], Emanuel H. Martin[148], Olivier Martin-Ducup[149,2], Michel M. Mbasi[150], Faustin Mbayu[134], Casimiro Mendoza[151], Irina Mendoza Polo[152,153], Gislain Mofack[76], Abel Monteagudo-Mendoza[154,155], Arafat Mtui[156], S. Muthuramkumar[157], Ayyappan Narayanan[133], María Guadalupe Nava-Miranda[158,159], David Neill[160], Victor John Neldner[161], Eric Ngansop Tchatchouang[162], Fernandez Ngoula[76], Michael R. Ngugi[161], Moses Nsanyi Sainge[30], Percy Núñez Vargas[155], Edgar Ortiz-Malavassi[163], Nadir Pallqui Camacho[6,155], Vivek Pandi[164], Germaine Alexander Parada Gutierrez[77], Guido Pardo[165], Narayanaswamy Parthasarathy[102], Raphael Pelissier[2], Sebastian Pfautsch[166], Bryan C.

Pijanowski[10], Daniel Piotto[167], John Pipoly[168,169], Nigel Pitman[170], Pierre Ploton[2], John R. Poulsen[171,95,172], B. R. Ramesh[133], Freddy Ramirez Arevalo[173], Hirma Ramírez-Angulo[174], L. Rasingam[175], Maxime Réjou-Méchain[2], Zorayda Restrepo Correa[176], Wilson Roberto Spironello[66], Cintia Rodrigues de Souza[177], Lily O. Rodriguez[178], Samir Rolim[167], Anand Roopsind[179], Francesco Rovero[180,181], Le Bienfaiteur Sagang[76], Purabi Saikia[182], Antonello Salis[13], Benoit Patrick Salla[76], Javier Eduardo Silva Espejo[183,184], Marcos Silveira[185], Murielle Simo-Droissart[2,76], Nelly Sirri[76], Bonaventure Sonké[76], Somaiah Sundarapandian[102], Michael Swaine[186], Ben Swanepoel[187], Hermann Taedoumg[188], Imma Tcheferi[76,189], John Terborgh[190,191], Duncan Thomas[192], Raquel Thomas[179], John Tshibamba Mukendi[193], Peter M. Umunay[194,195], Renato Valencia[115], Luis Valenzuela Gamarra[154], Leandro Valle Ferreira[196], Geertje van der Heijden[197], Rodolfo Vasquez Martinez[154], José Daniel Vega-Nieva[90], Emilio Vilanova[198], Braulio Vilchez Alvarado[163], Barbara Vinceti[199], Jason Vleminckx[200], Klaus von Gadow[201], Vincent Antoine Vos[202], Eric Wasingya[134], James Watson[203], Verginia Wortel[204], Roderick Zagt[205], Donatien Zébazé[76,1], ForestPlots.net, GFBI, Jean-François Bastin[1*]

[*]Corresponding authors: depoort.p@gmail.com, jfbastin@uliege.be

ForestPlots.net and GFBI are author consortia; the full lists of their members and affiliations appear at the end of the paper, after the references.

Figures.

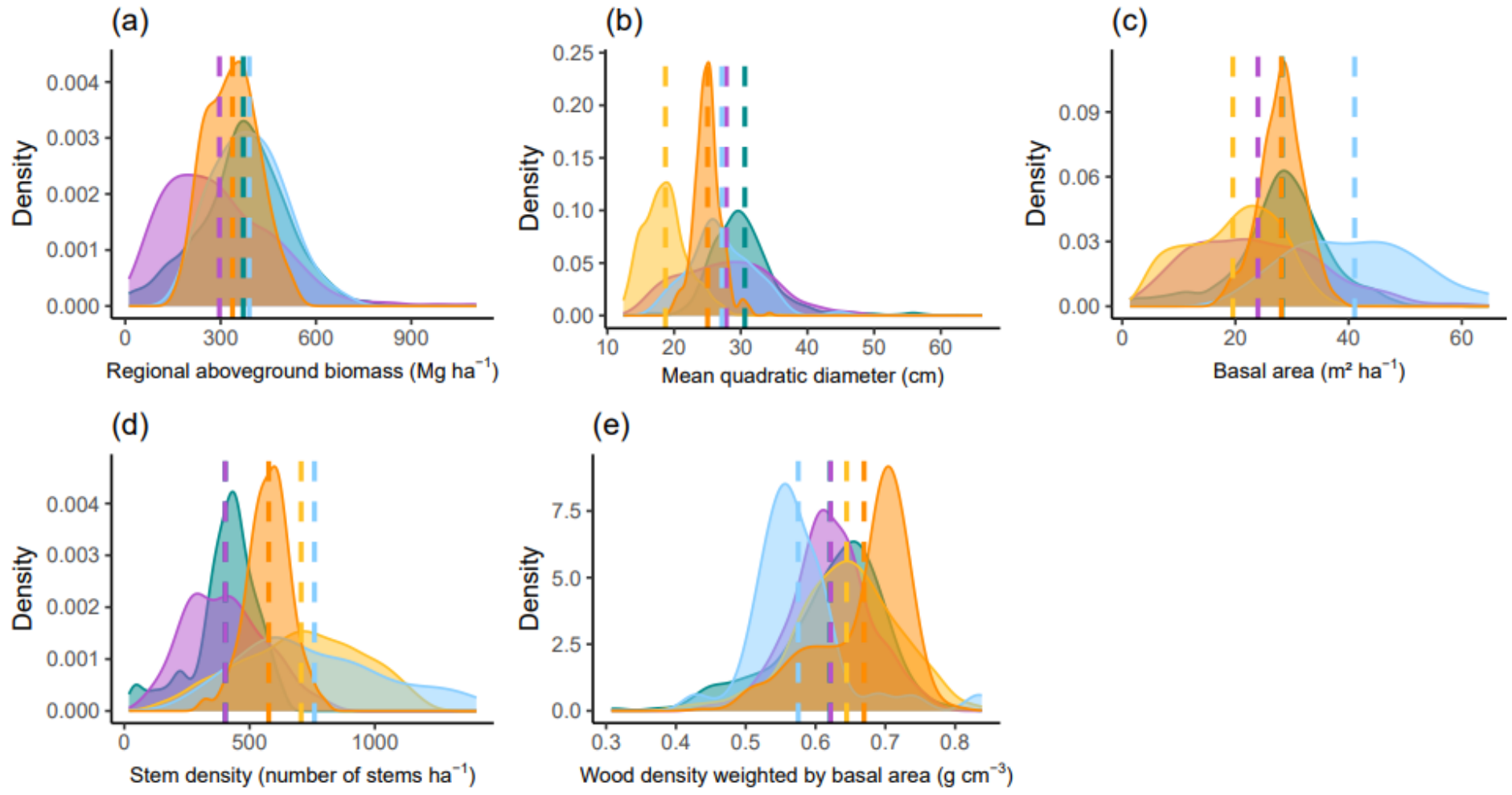

Extended Data Figure 1. Variations in forest structure and aboveground biomass (AGB) across tropical regions, excluding montane forests. AGB and structural attribute distributions by region (in the same colors as shown on the map, Figure 1), shown as probability density functions. Dashed lines indicate the mean values. Results are shown for AGB estimated using tree height derived from the region-specific height-diameter (H-D) model of Feldpausch et al. (see Methods)[43]. Figure 1 shows the distribution of plots including montane forests, and Extended Data Figures 2–3 show the AGB distribution estimated using the pantropical and continent-specific H-D models[43].

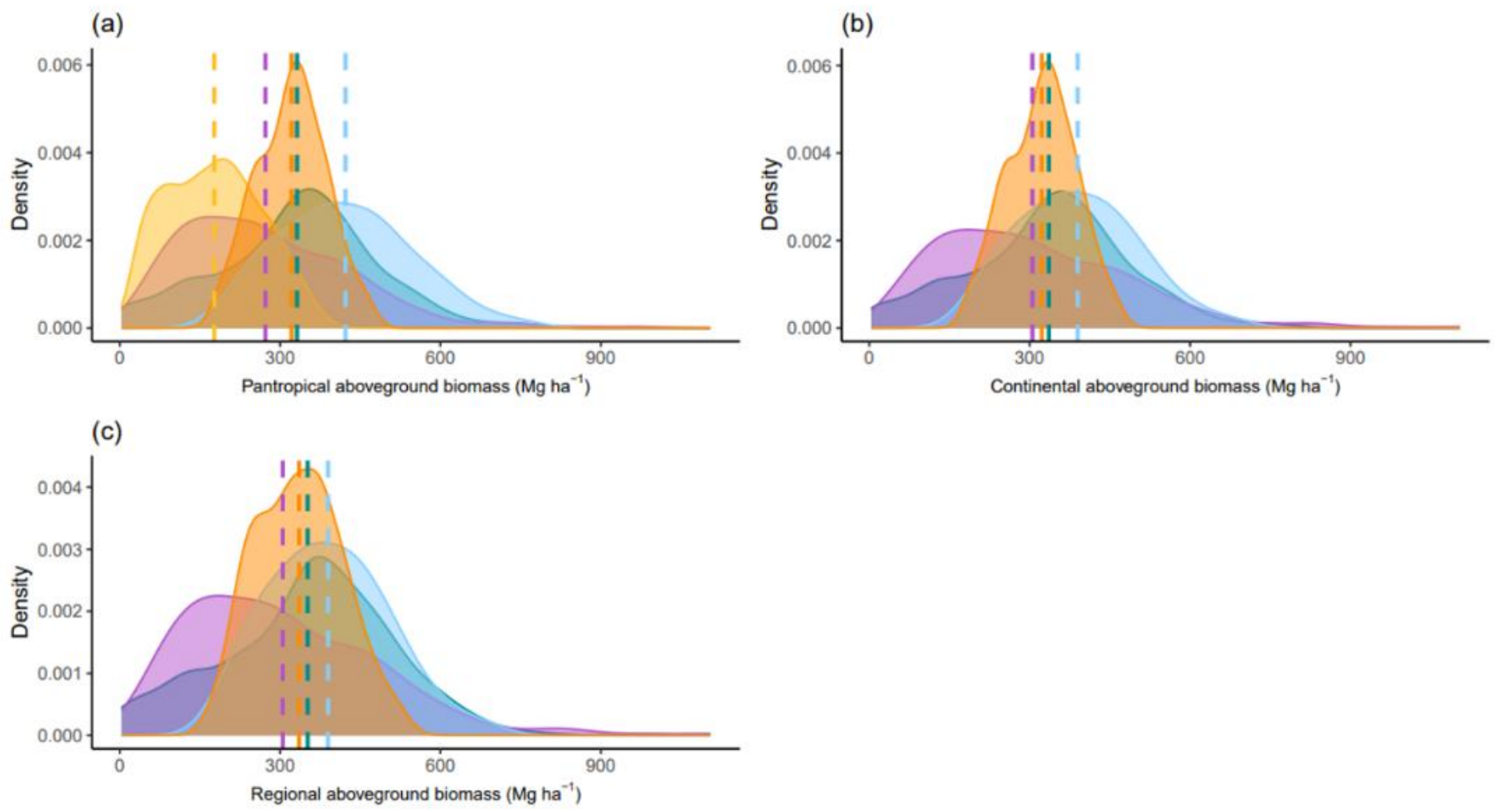

Extended Data Figure 2. Variation in aboveground biomass (AGB) across tropical regions, including montane forests. Distribution of plot-level AGB estimated using the tree-level equation (4) from Chave et al.[49] **(a)** with the pantropical, **(b)** with the continent-specific, and **(c)** with the region-specific H-D models of Feldpausch et al. (see Methods)[43]. AGB distributions are presented as probability density functions for each region (in the same colors as shown on the map, Figure 1). Dashed lines indicate mean AGB per region.

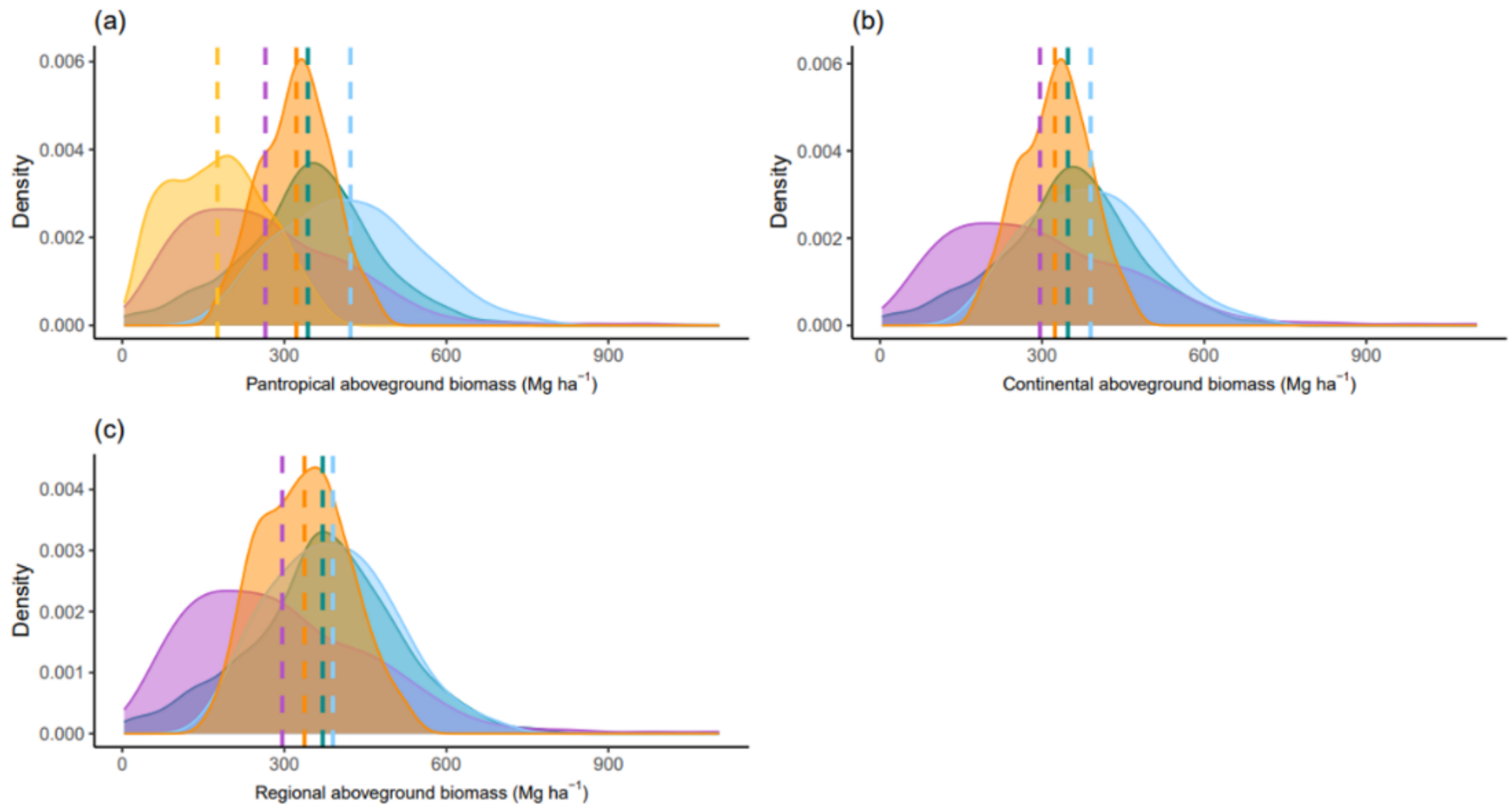

Extended Data Figure 3. Variation in aboveground biomass (AGB) across tropical regions, excluding montane forests. Distribution of plot-level AGB estimated using the tree-level equation (4) from Chave et al.[49] **(a)** with the pantropical, **(b)** with the continent-specific, and **(c)** with the region-specific H-D models of Feldpausch et al. (see Methods)[43]. AGB distributions are presented as probability density functions for each region (in the same colors as shown on the map, Figure 1). Dashed lines indicate mean AGB per region.

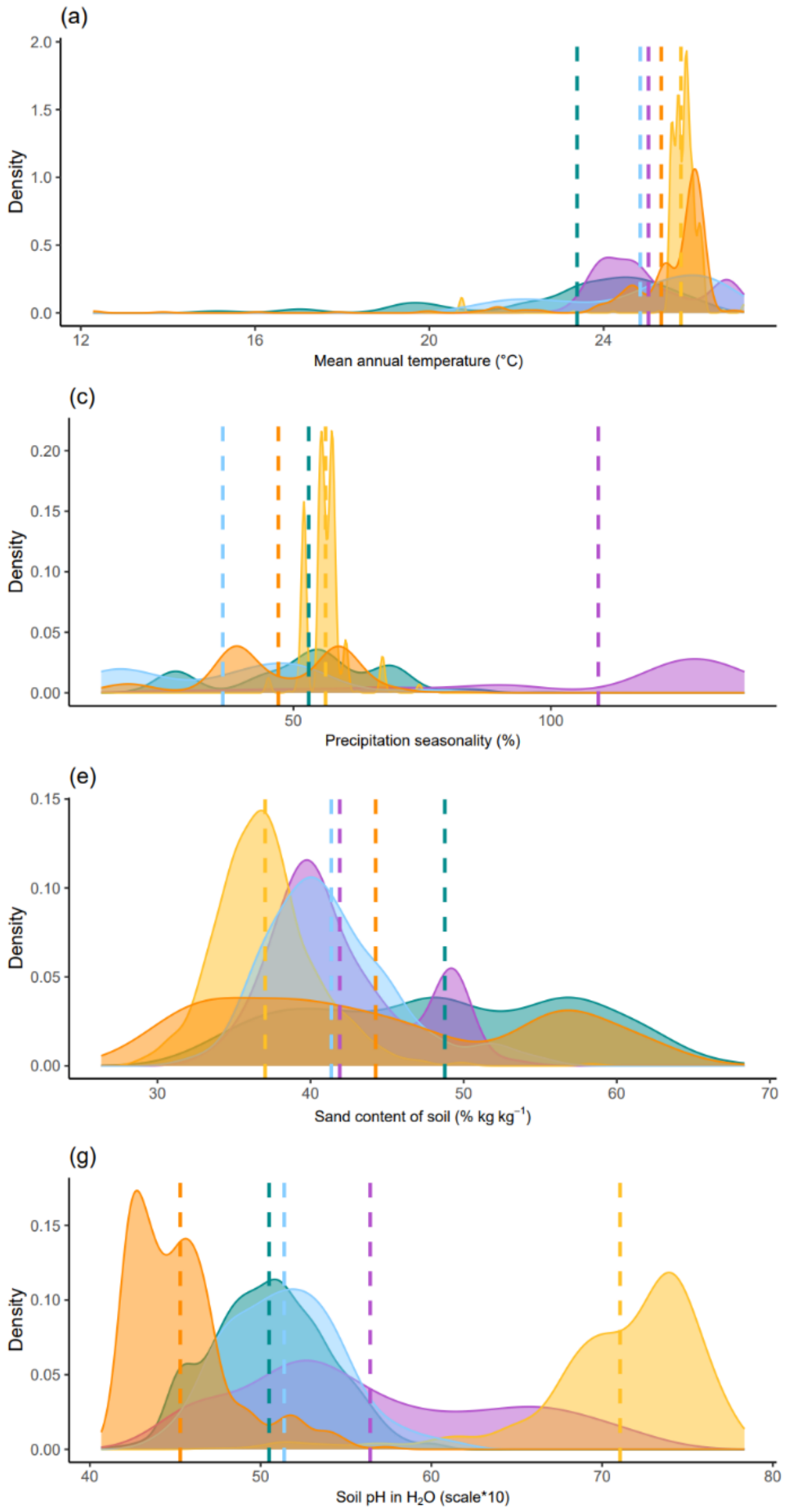

Extended Data Figure 4. Climatic, soil, and topographic variation across tropical regions, including montane forests. Distribution of the seven selected environmental variables (see Methods), shown as probability density functions for each region (in the same colors as shown on the map, Figure 1). Dashed lines indicate the mean values. Climatic variables are derived from the CHELSA datasets (see Methods). See Extended Data Figure 5 for results excluding montane forests (see Methods).

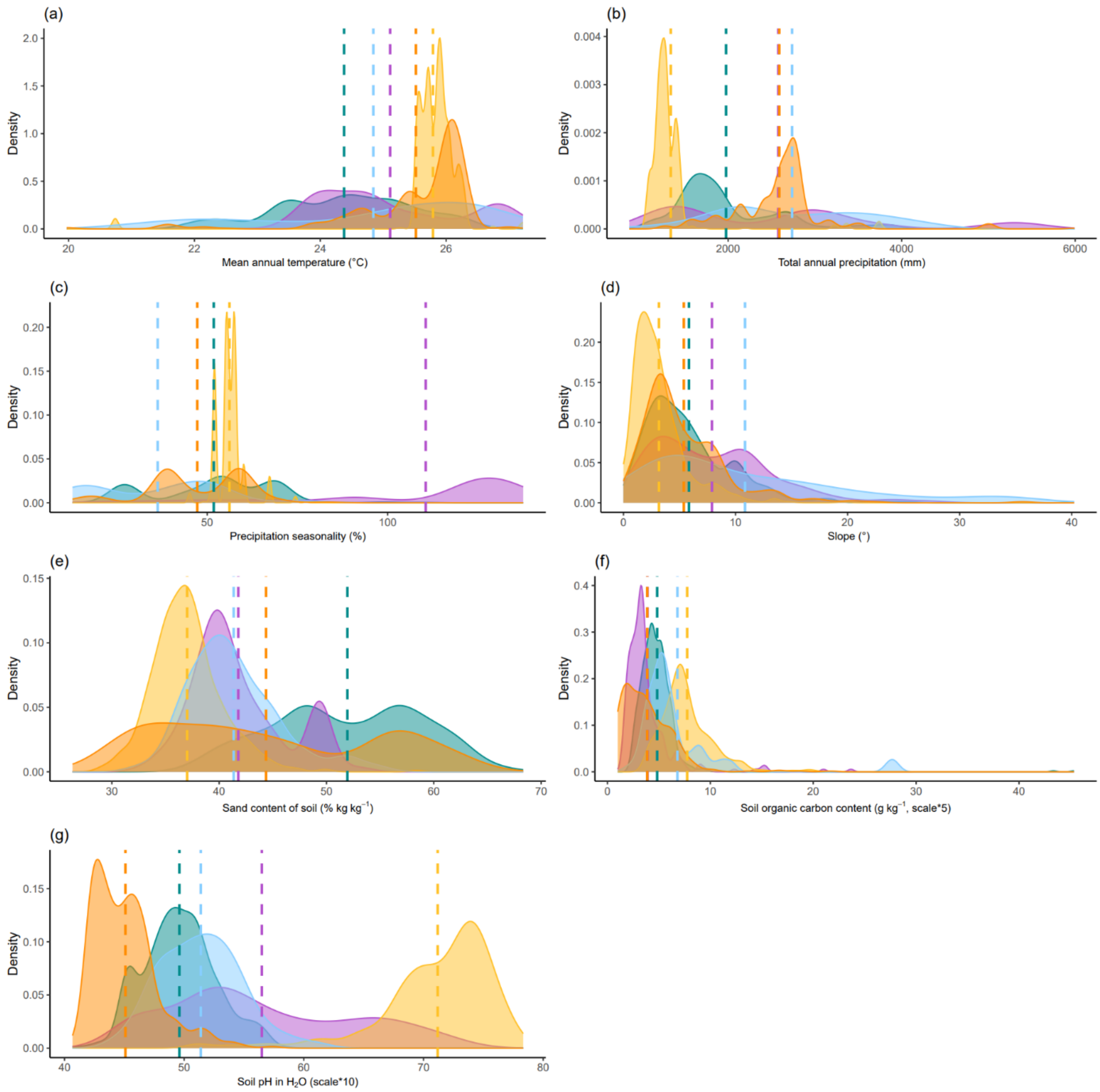

Extended Data Figure 5. Climatic, soil, and topographic variation across tropical regions, excluding montane forests. Distribution of the seven selected environmental variables (see Methods), shown as probability density functions for each region (in the same colors as shown on the map, Figure 1). Dashed lines indicate the mean values. Climatic variables are derived from the CHELSA datasets (see Methods). See Extended Data Figure 4 for results including montane forests (see Methods).

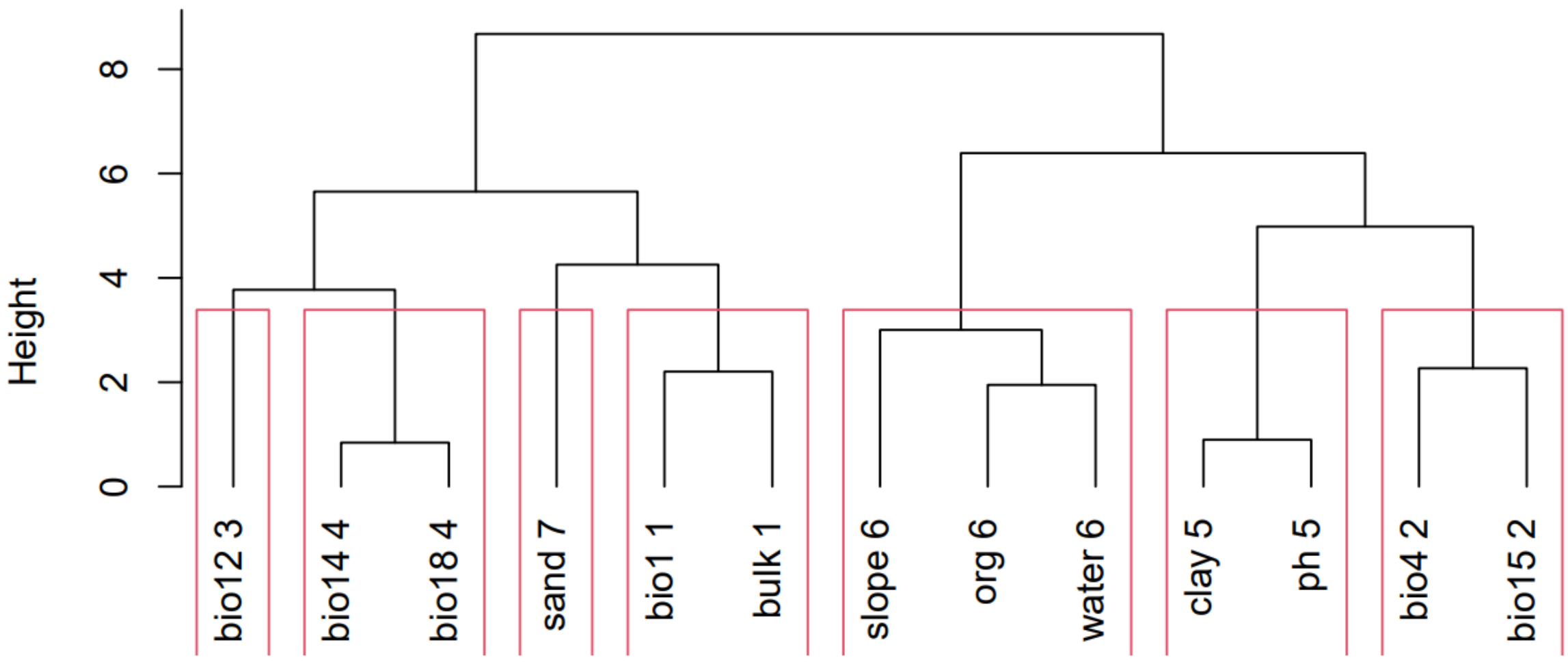

Extended Data Figure 6. Selection of the environmental variables. Dendrogram based on a clustering analysis of environmental variables using the first four axes of a principal component analysis (see Methods). Seven groups are identified, indicated by red squares. Environmental variables include mean annual temperature (bio1), total annual precipitation (bio12), precipitation of driest month (bio14), precipitation seasonality (bio15), precipitation of warmest quarter (bio18), soil sand content (sand), soil bulk density (bulk), soil organic carbon content (org), soil water content (water), soil clay content (clay), soil pH in H_2O (ph), and topographic slope. Climatic variables were obtained from the WorldClim dataset (see Methods).

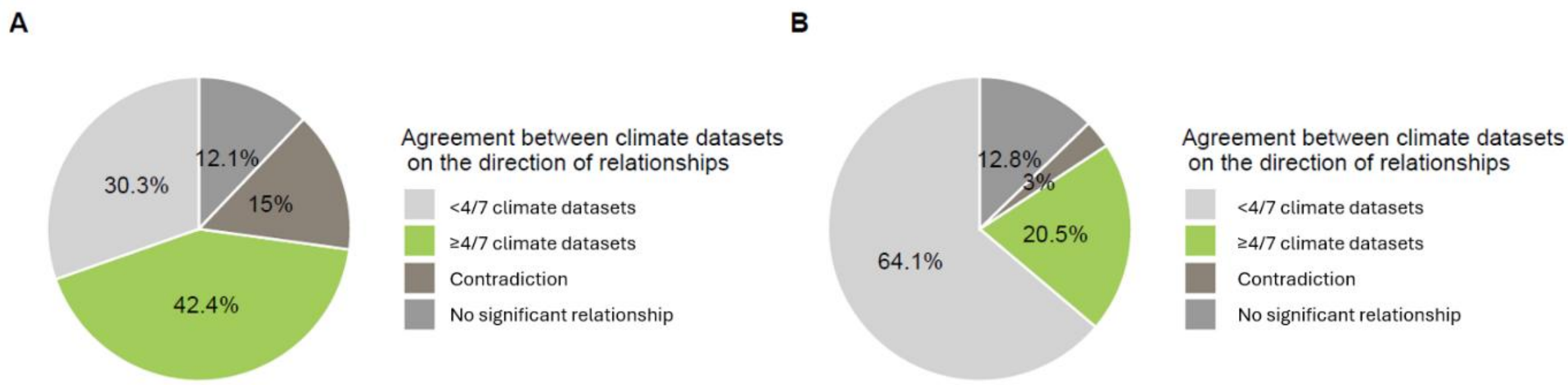

Extended Data Figure 7. Impact of the choice of the climate data source, including montane forests. Percentage of agreement between climate data sources on the direction of the **(A)** influence of climate on structural attributes and **(B)** the total influence of climate on aboveground biomass (AGB), at the regional scale, when changing the climate data source seven times in the models, derived from the structure-explicit pathway modelling framework (see Methods). The level of agreement is classified into four categories: "≥4/7 climate sources" indicates that at least four climate data sources detect an effect in the same direction, while the others detect no significant effect; "<4/7 climate sources" indicates that fewer than four climate data sources detect an effect in the same direction, while the others detect no significant effect; "Contradiction" indicates that both positive and negative effects were detected; and "No significant relationship" refers to cases where no significant effect was detected by any climate data source (p-value ≥ 0.05). The percentage of agreement is calculated using the results of all regions and selected bioclimatic variables together and includes results for AGB estimates based on the three height-diameter (H-D) models of Feldpausch et al.[43]: the pantropical, continent- and region-specific H-D models (see Methods).

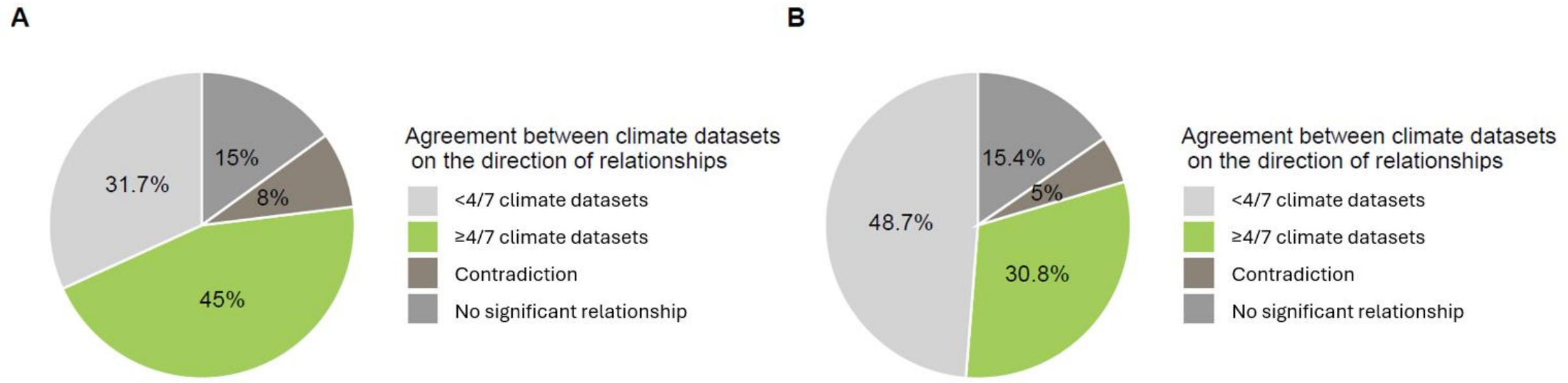

Extended Data Figure 8. Impact of the choice of the climate data source, excluding montane forests. Percentage of agreement between climate data sources on the direction of the **(A)** influence of climate on structural attributes and **(B)** the total influence of climate on aboveground biomass (AGB), at the regional scale, when changing the climate data source seven times in the models, derived from the structure-explicit pathway modelling framework (see Methods). The level of agreement is classified into four categories: "≥4/7 climate sources" indicates that at least four climate data sources detect an effect in the same direction, while the others detect no significant effect; "<4/7 climate sources" indicates that fewer than four climate data sources detect an effect in the same direction, while the others detect no significant effect; "Contradiction" indicates that both positive and negative effects were detected; and "No significant relationship" refers to cases where no significant effect was detected by any climate data source (p-value ≥ 0.05). The percentage of agreement is calculated using the results of all regions and selected bioclimatic variables together and includes results for AGB estimates based on the three height-diameter (H-D) models of Feldpausch et al.[43]**:** the pantropical, continent- and region-specific H-D models (see Methods).

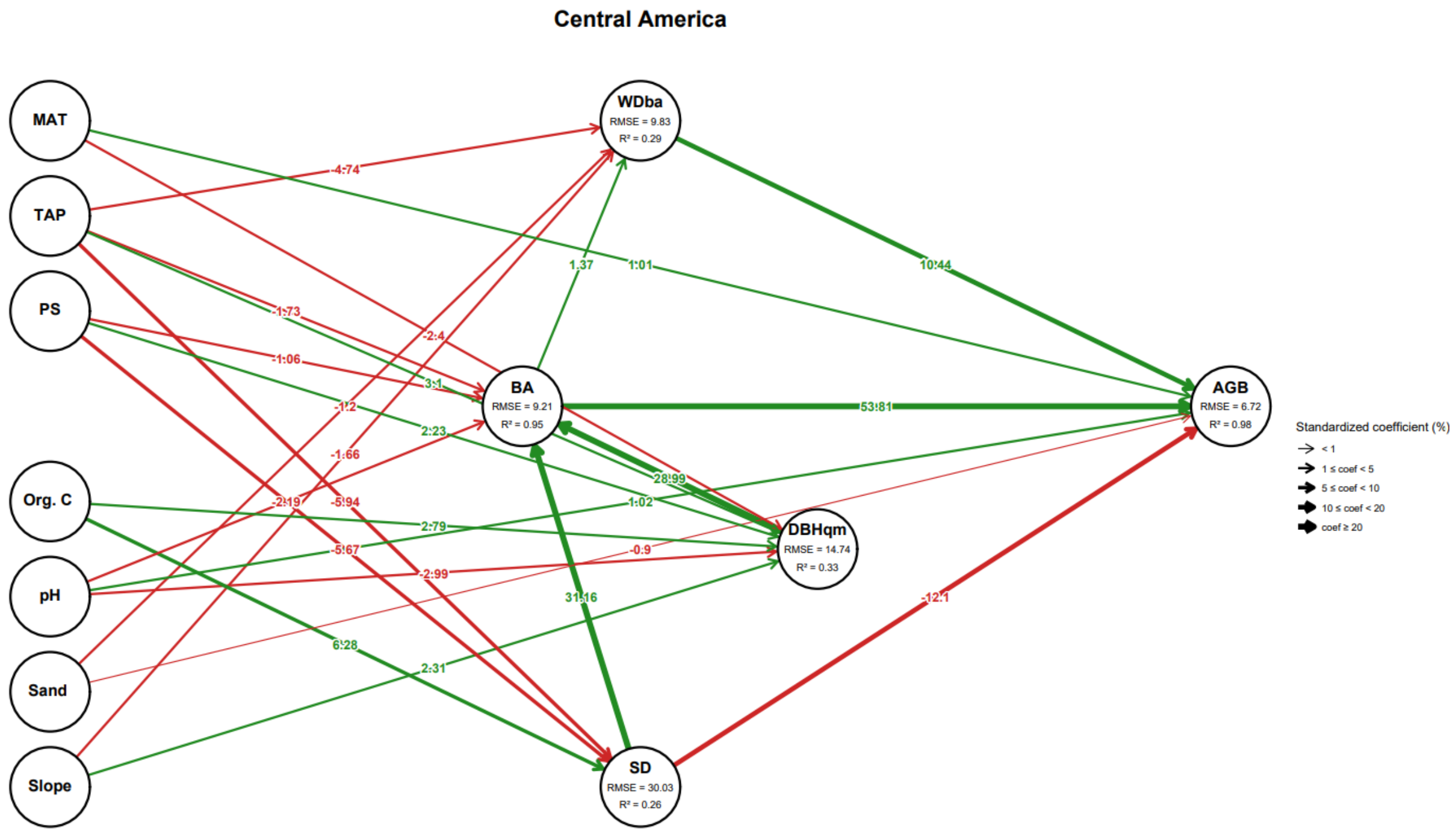

Extended Data Figure 9. Environmental effects on aboveground biomass (AGB) mediated by forest structure in Central America. Indirect and (residual) direct relationships between environmental variables and AGB (estimated using the pantropical height–diameter (H-D) models), derived from the structure-explicit pathway modelling framework (see Methods). Variables are shown as circles: environmental variables on the left, structural attributes in the center, and AGB on the right. Structural attributes include basal area (BA), wood density weighted by basal area (WDba), quadratic mean diameter (DBHqm), and stem density (SD). Environmental variables include mean annual temperature (MAT), total annual precipitation (TAP), precipitation seasonality (PS), soil sand content (Sand), soil organic carbon content (Org. C), soil pH in H_2O (pH), and topographic slope (Slope). Moran's Eigenvector Maps, used to control for spatial autocorrelation, are not included in the figure. Arrows represent significant relationships; the absence of an arrow indicates no significant direct relationship (p-value ≥ 0.05). The values on each arrow correspond to standardized coefficients of the relationships, expressed as percentages relative to the mean of the response variable per standard deviation (%). For each response variable, the figure reports the R^2 value and the root mean square error (RMSE), expressed as a percentage of the mean response variable. Coefficients in raw, standardized, and % units are reported in Extended Data Table 5. For the three climate variables, values represent the average of the coefficients obtained using different climate data sources (see Methods). The same patterns held when using the continent- and region-specific H-D models of Feldpausch et al.[43] to estimate AGB (Extended Data Table 5).

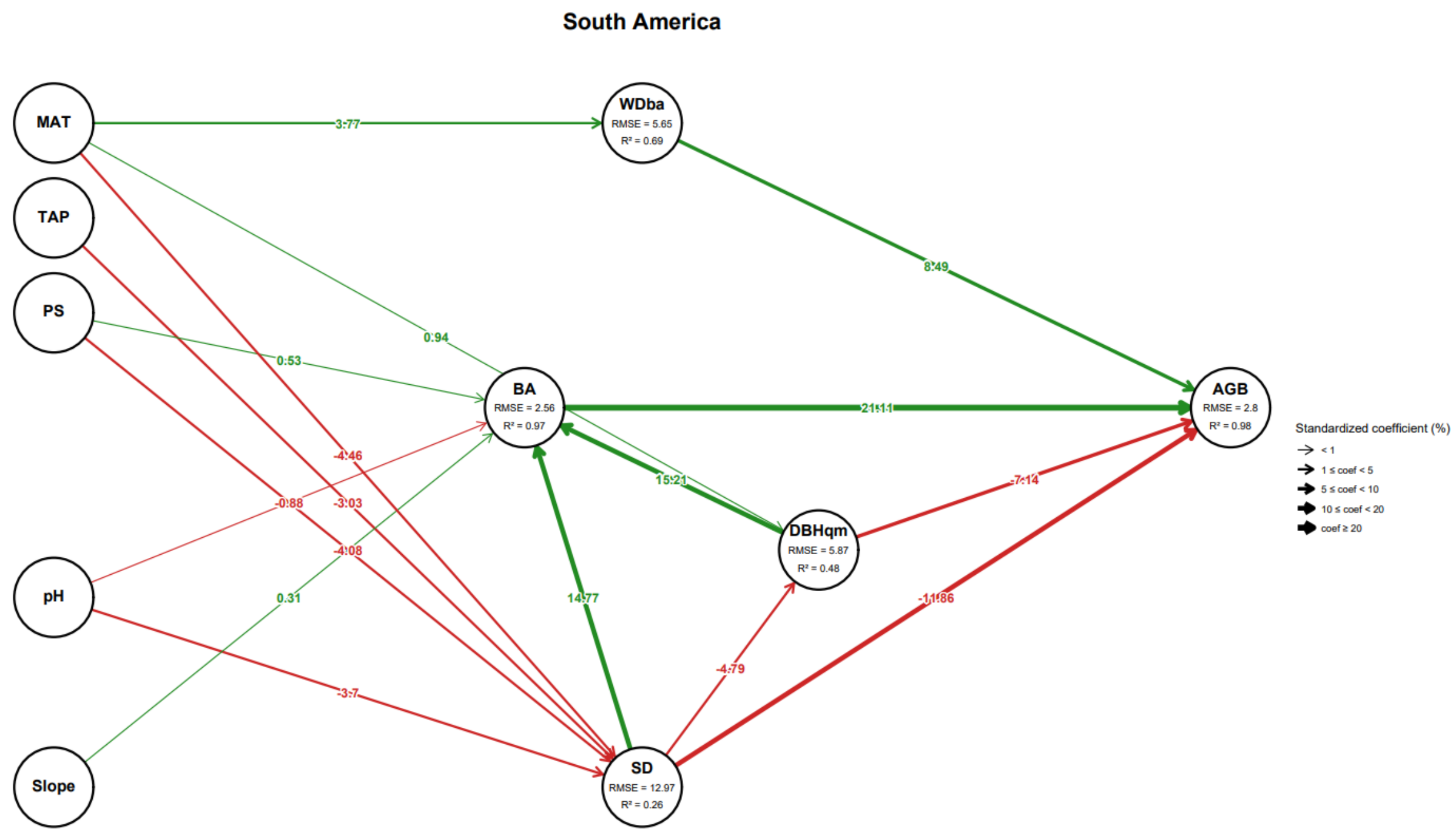

Extended Data Figure 10. Environmental effects on aboveground biomass (AGB) mediated by forest structure in South America. Indirect and (residual) direct relationships between environmental variables and AGB (estimated using the pantropical height–diameter (H-D) models) derived from the structure-explicit pathway modelling framework (see Methods). Variables are shown as circles: environmental variables on the left, structural attributes in the center, and AGB on the right. Structural attributes include basal area (BA), wood density weighted by basal area (WDba), quadratic mean diameter (DBHqm), and stem density (SD). Environmental variables include mean annual temperature (MAT), total annual precipitation (TAP), precipitation seasonality (PS), soil sand content (Sand), soil organic carbon content (Org. C), soil pH in H_2O (pH), and topographic slope (Slope). Moran's Eigenvector Maps, used to control for spatial autocorrelation, are not included in the figure. Arrows represent significant relationships; the absence of an arrow indicates no significant direct relationship (p-value ≥ 0.05). The values on each arrow correspond to standardized coefficients of the relationships, expressed as percentages relative to the mean of the response variable per standard deviation (%). For each response variable, the figure reports the R^2 value and the root mean square error (RMSE), expressed as a percentage of the mean response variable. Coefficients in raw, standardized, and % units are reported in Extended Data Table 5. For the three climate variables, values represent the average of the coefficients obtained using different climate data sources (see Methods). The same patterns held when using the continent- and region-specific H-D models of Feldpausch et al.[43] to estimate AGB (Extended Data Table 5).

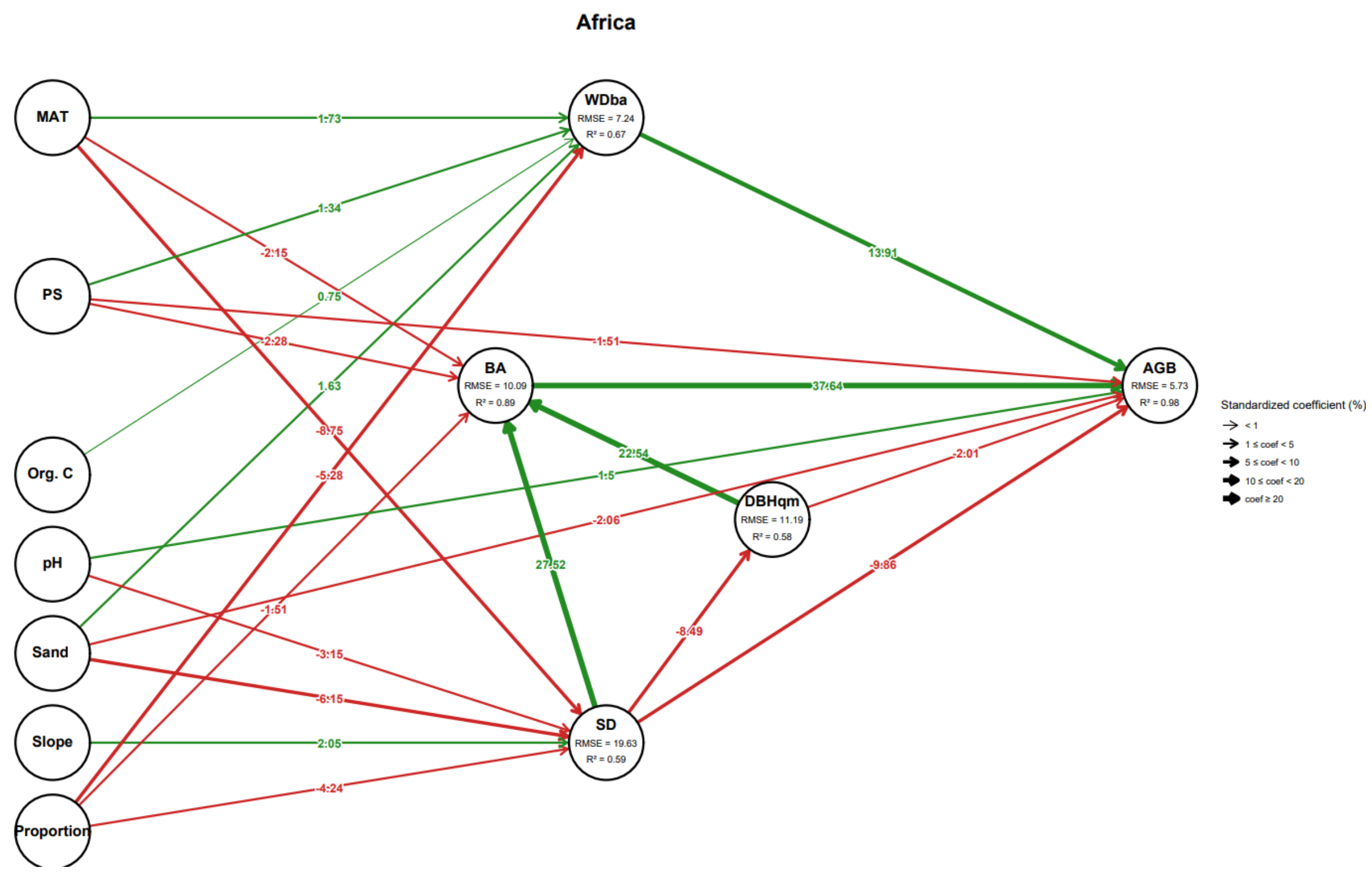

Extended Data Figure 11. Environmental effects on aboveground biomass (AGB) mediated by forest structure in Africa, excluding montane forests and controlling for the proportion of pioneer. Indirect and (residual) direct relationships between environmental variables and AGB (estimated using the pantropical height–diameter (H-D) models) derived from the structure-explicit pathway modelling framework (see Methods). Variables are shown as circles: environmental variables and % of pioneer species ("Proportion") on the left (see Methods), structural attributes in the center, and AGB on the right. Structural attributes include basal area (BA), wood density weighted by basal area (WDba), quadratic mean diameter (DBHqm), and stem density (SD). Environmental variables include mean annual temperature (MAT), total annual precipitation (TAP), precipitation seasonality (PS), soil sand content (Sand), soil organic carbon content (Org. C), soil pH in H_2O (pH), and topographic slope (Slope). Moran's Eigenvector Maps, used to control for spatial autocorrelation, are not included in the figure. Arrows represent significant relationships; the absence of an arrow indicates no significant direct relationship (p-value ≥ 0.05). The values on each arrow correspond to standardized coefficients of the relationships, expressed as percentages relative to the mean of the response variable per standard deviation (%). For each response variable, the figure reports the R^2 value and the root mean square error (RMSE), expressed as a percentage of the mean response variable. Coefficients in raw, standardized, and % units are reported in Extended Data Table 5. For the three climate variables, values represent the average of the coefficients obtained using different climate data sources (see Methods). The same patterns held when using the continent- and region-specific H-D models of Feldpausch et al.[43] to estimate AGB (Extended Data Table 5) and when including montane forests (Extended Data Figure 15 and Extended Data Table 6).

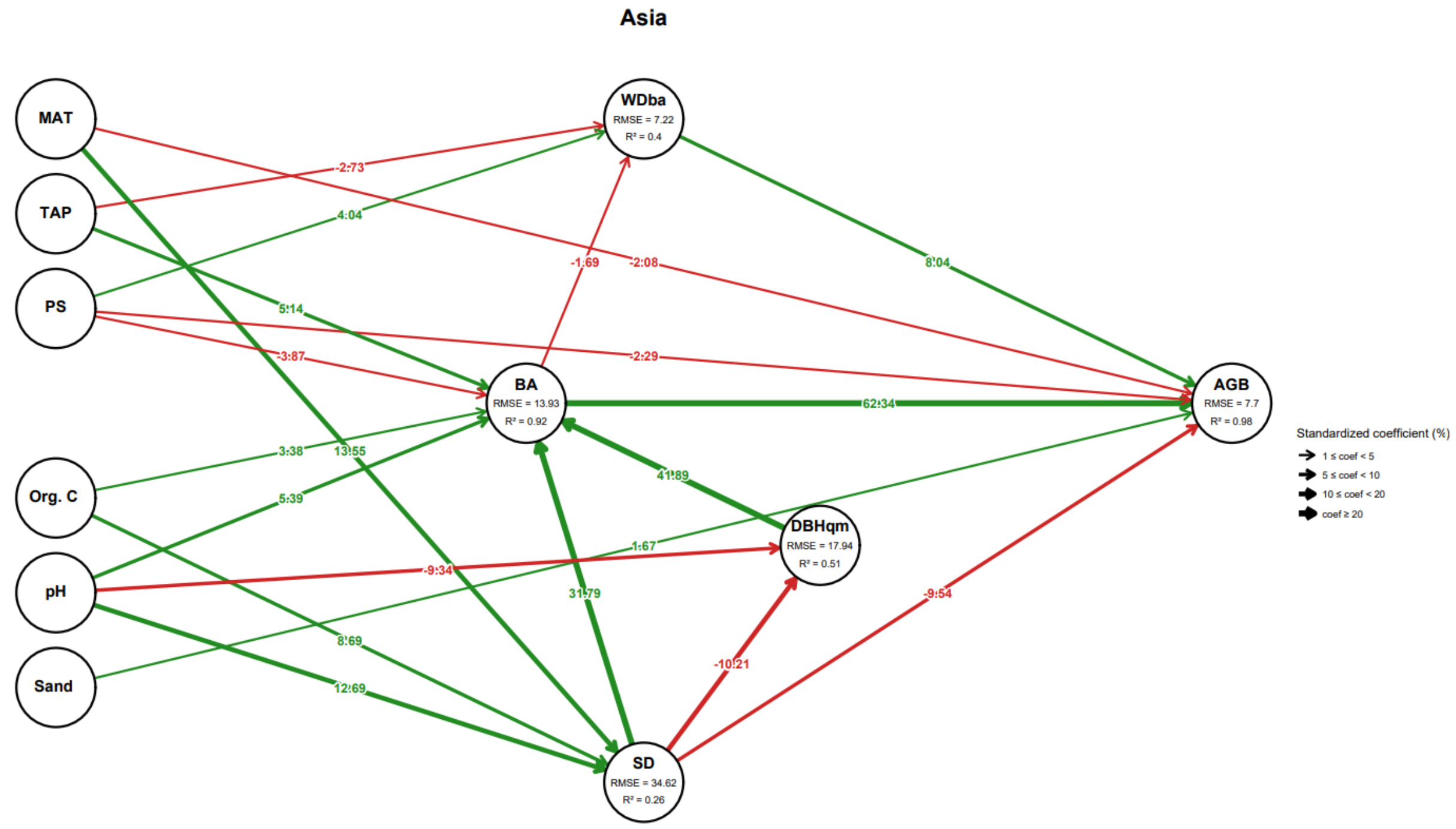

Extended Data Figure 12. Environmental effects on aboveground biomass (AGB) mediated by forest structure in Asia, excluding montane forests. Indirect and (residual) direct relationships between environmental variables and AGB (estimated using the pantropical height–diameter (H-D) models) derived from the structure-explicit pathway modelling framework (see Methods). Variables are shown as circles: environmental variables on the left, structural attributes in the center, and AGB on the right. Structural attributes include basal area (BA), wood density weighted by basal area (WDba), quadratic mean diameter (DBHqm), and stem density (SD). Environmental variables include mean annual temperature (MAT), total annual precipitation (TAP), precipitation seasonality (PS), soil sand content (Sand), soil organic carbon content (Org. C), soil pH in H_2O (pH), and topographic slope (Slope). Moran's Eigenvector Maps, used to control for spatial autocorrelation, are not included in the figure. Arrows represent significant relationships; the absence of an arrow indicates no significant direct relationship (p-value ≥ 0.05). The values on each arrow correspond to standardized coefficients of the relationships, expressed as percentages relative to the mean of the response variable per standard deviation (%). For each response variable, the figure reports the R^2 value and the root mean square error (RMSE), expressed as a percentage of the mean response variable. Coefficients in raw, standardized, and % units are reported in Extended Data Table 5. For the three climate variables, values represent the average of the coefficients obtained using different climate data sources (see Methods). The same patterns held when using the continent- and region-specific H-D models of Feldpausch et al.[43] to estimate AGB (Extended Data Table 5) and when including montane forests (Extended Data Figure 16 and Extended Data Table 6).

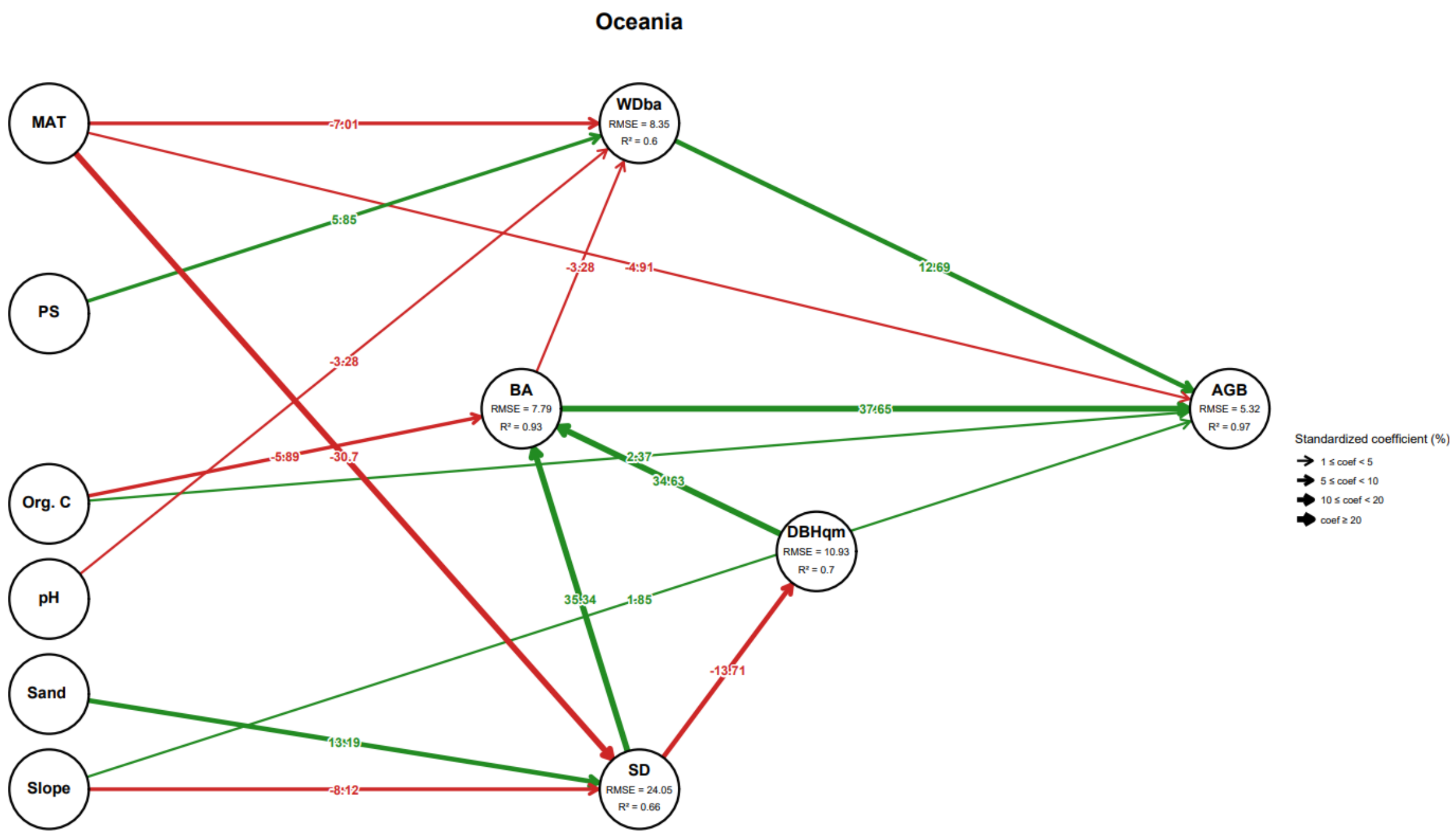

Extended Data Figure 13. Environmental effects on aboveground biomass (AGB) mediated by forest structure in Oceania. Indirect and (residual) direct relationships between environmental variables and AGB (estimated using the pantropical height–diameter (H-D) models) derived from the structure-explicit pathway modelling framework (see Methods). Variables are shown as circles: environmental variables on the left, structural attributes in the center, and AGB on the right. Structural attributes include basal area (BA), wood density weighted by basal area (WDba), quadratic mean diameter (DBHqm), and stem density (SD). Environmental variables include mean annual temperature (MAT), total annual precipitation (TAP), precipitation seasonality (PS), soil sand content (Sand), soil organic carbon content (Org. C), soil pH in H_2O (pH), and topographic slope (Slope). Moran's Eigenvector Maps, used to control for spatial autocorrelation, are not included in the figure. Arrows represent significant relationships; the absence of an arrow indicates no significant direct relationship (p-value ≥ 0.05). The values on each arrow correspond to standardized coefficients of the relationships, expressed as percentages relative to the mean of the response variable per standard deviation (%). For each response variable, the figure reports the R^2 value and the root mean square error (RMSE), expressed as a percentage of the mean response variable. Coefficients in raw, standardized, and % units are reported in Extended Data Table 5. For the three climate variables, values represent the average of the coefficients obtained using different climate data sources (see Methods). The same patterns held when using the continent- and region-specific H-D models of Feldpausch et al.[43] to estimate AGB (Extended Data Table 5).

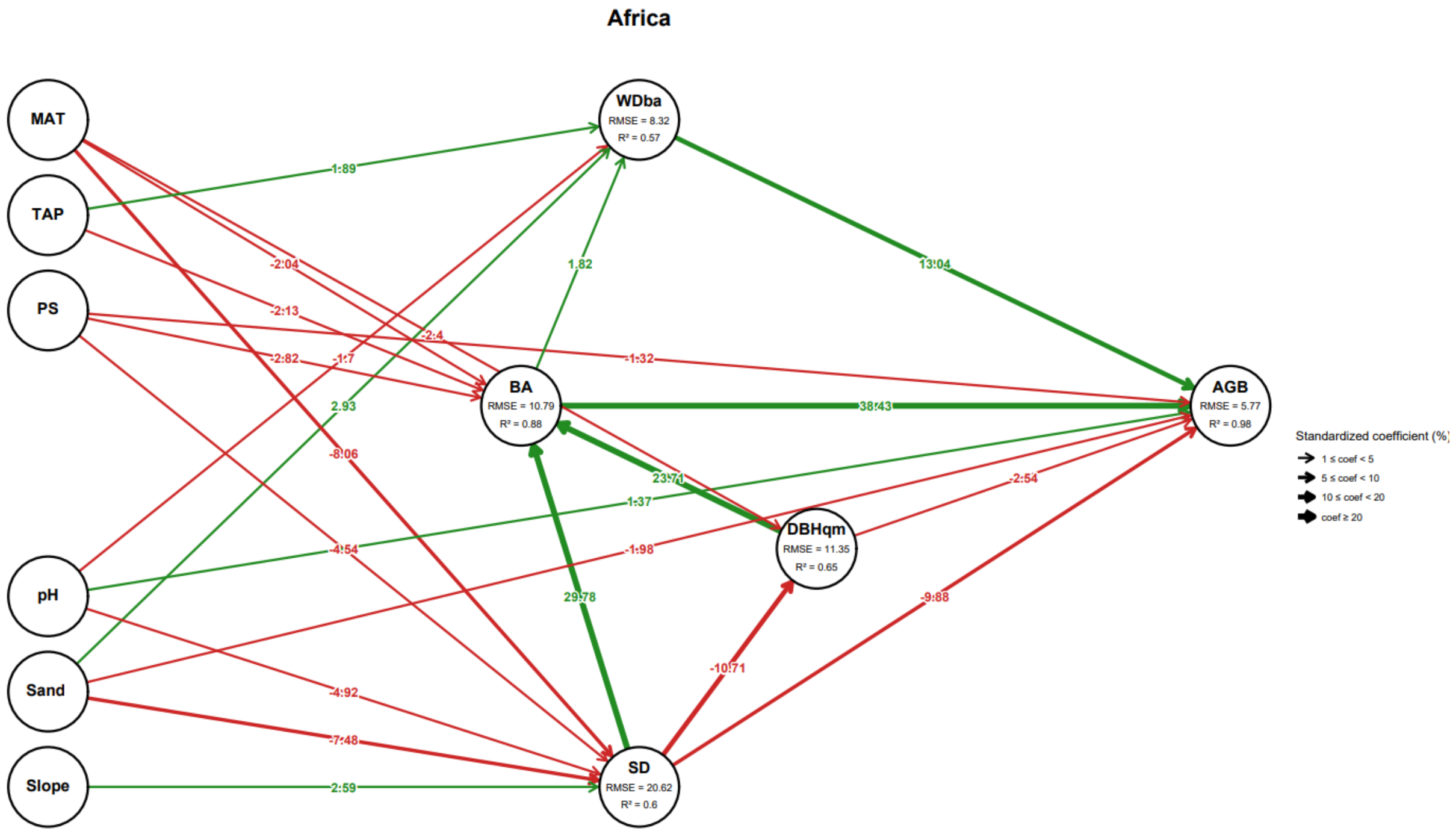

Extended Data Figure 14. Environmental effects on aboveground biomass (AGB) mediated by forest structure in Africa, excluding montane forests. Indirect and (residual) direct relationships between environmental variables and AGB (estimated using the pantropical height–diameter (H-D) models) derived from the structure-explicit pathway modelling framework (see Methods). Variables are shown as circles: environmental variables on the left, structural attributes in the center, and AGB on the right. Structural attributes include basal area (BA), wood density weighted by basal area (WDba), quadratic mean diameter (DBHqm), and stem density (SD). Environmental variables include mean annual temperature (MAT), total annual precipitation (TAP), precipitation seasonality (PS), soil sand content (Sand), soil organic carbon content (Org. C), soil pH in H_2O (pH), and topographic slope (Slope). Moran's Eigenvector Maps, used to control for spatial autocorrelation, are not included in the figure. Arrows represent significant relationships; the absence of an arrow indicates no significant direct relationship (p-value ≥ 0.05). The values on each arrow correspond to standardized coefficients of the relationships, expressed as percentages relative to the mean of the response variable per standard deviation (%). For each response variable, the figure reports the R^2 value and the root mean square error (RMSE), expressed as a percentage of the mean response variable. Coefficients in raw, standardized, and % units are reported in Extended Data Table 5. For the three climate variables, values represent the average of the coefficients obtained using different climate data sources (see Methods). The same patterns held when using the continent- and region-specific H-D models of Feldpausch et al.[43] to estimate AGB (Extended Data Table 5) and when including montane forests (Extended Data Figure 17 and Extended Data Table 6).

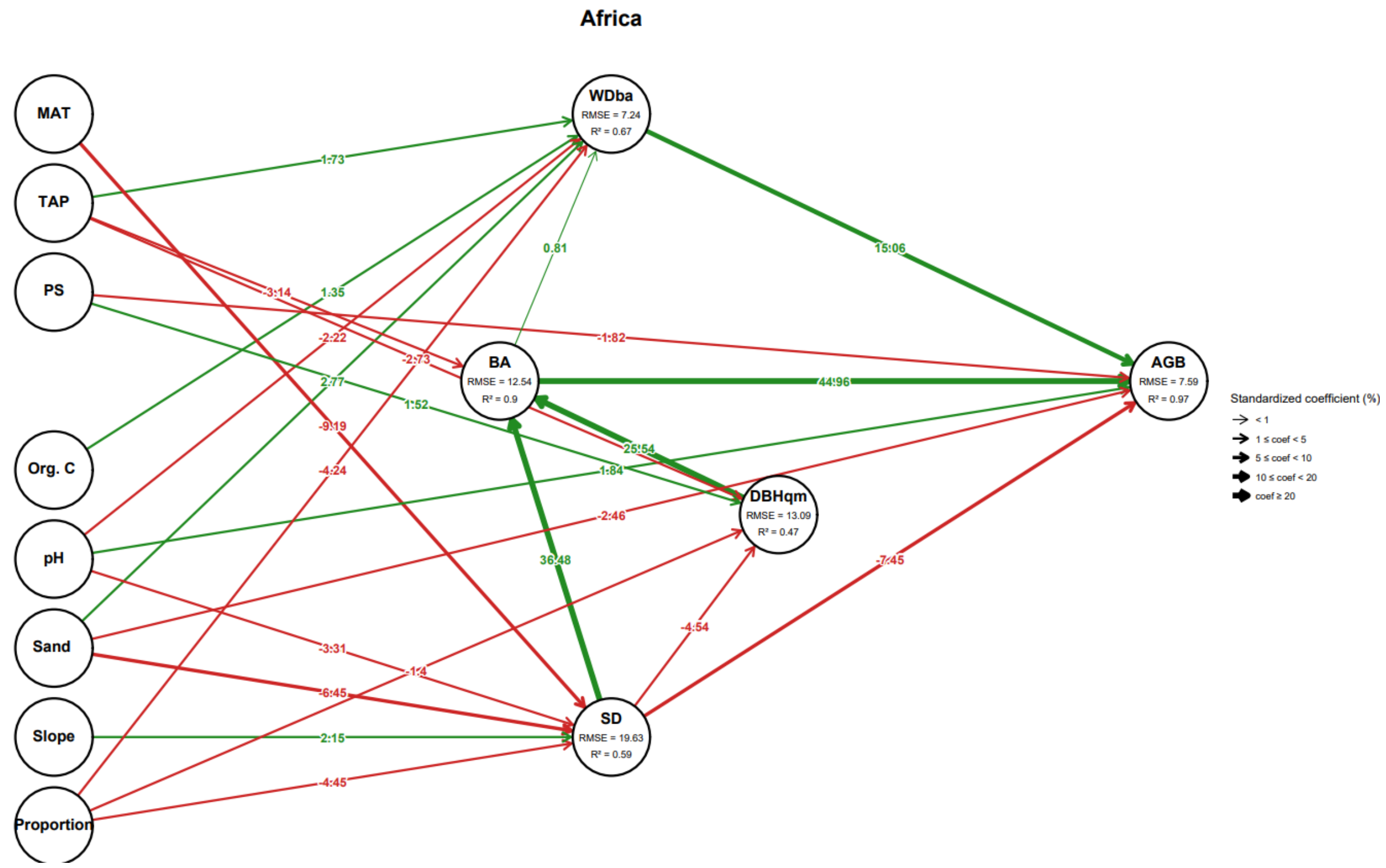

Extended Data Figure 15. Environmental effects on aboveground biomass (AGB) mediated by forest structure in Africa, including montane forests and controlling for the proportion of pioneer. Indirect and (residual) direct relationships between environmental variables and AGB (estimated using the pantropical height–diameter (H-D) models) derived from the structure-explicit pathway modelling framework (see Methods). Variables are shown as circles: environmental variables and % of pioneer species ("Proportion") on the left (see Methods), structural attributes in the center, and AGB on the right. Structural attributes include basal area (BA), wood density weighted by basal area (WDba), quadratic mean diameter (DBHqm), and stem density (SD). Environmental variables include mean annual temperature (MAT), total annual precipitation (TAP), precipitation seasonality (PS), soil sand content (Sand), soil organic carbon content (Org. C), soil pH in H_2O (pH), and topographic slope (Slope). Moran's Eigenvector Maps, used to control for spatial autocorrelation, are not included in the figure. Arrows represent significant relationships; the absence of an arrow indicates no significant direct relationship (p-value ≥ 0.05). The values on each arrow correspond to standardized coefficients of the relationships, expressed as percentages relative to the mean of the response variable per standard deviation (%). For each response variable, the figure reports the R^2 value and the root mean square error (RMSE), expressed as a percentage of the mean response variable. Coefficients in raw, standardized, and % units are reported in Extended Data Table 6. For the three climate variables, values represent the average of the coefficients obtained using different climate data sources (see Methods). The same patterns held when using the continent- and region-specific H-D models of Feldpausch et al.[43] to estimate AGB (Extended Data Table 6).

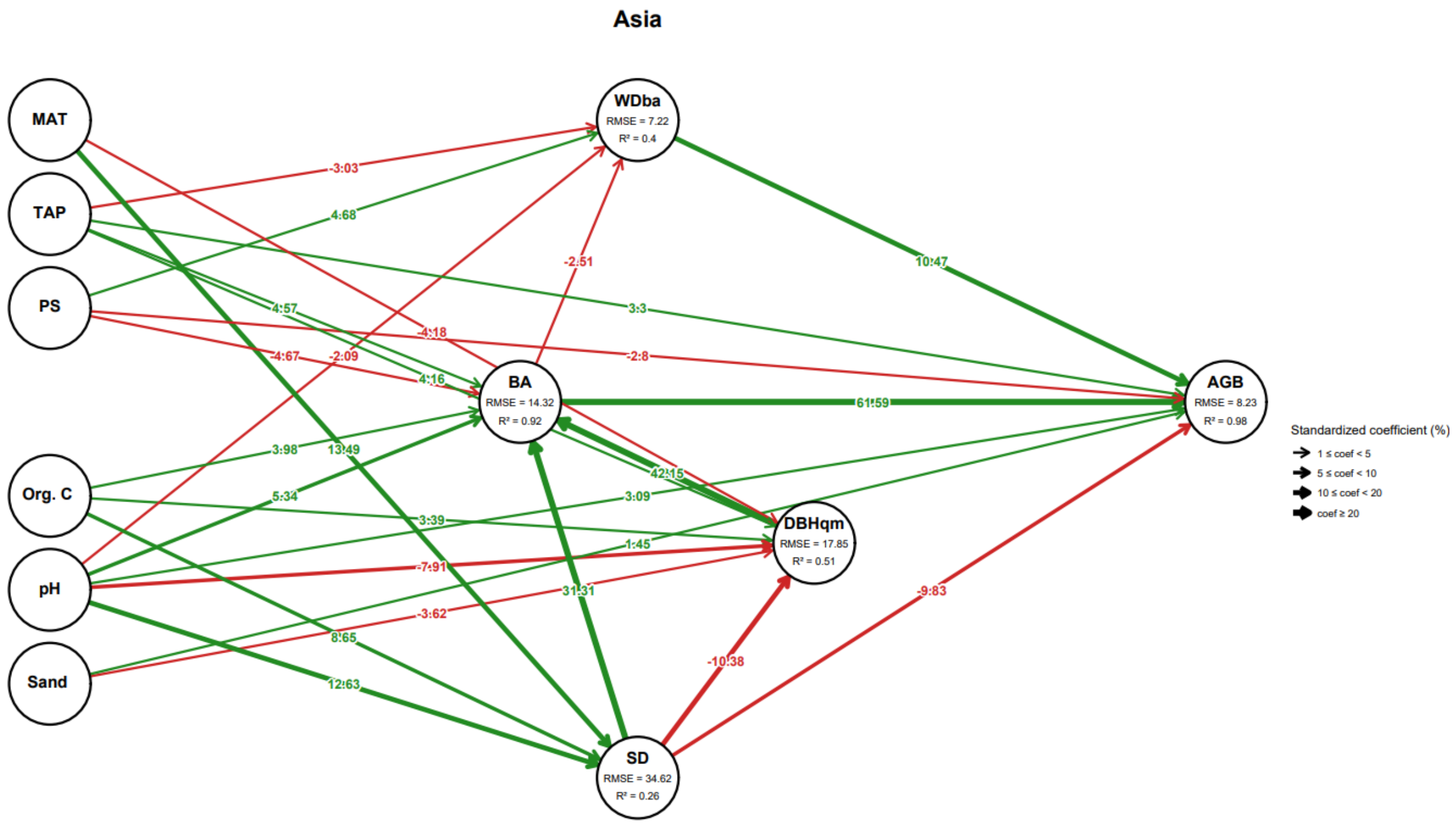

Extended Data Figure 16. Environmental effects on aboveground biomass (AGB) mediated by forest structure in Asia, including montane forests. Indirect and (residual) direct relationships between environmental variables and AGB (estimated using the pantropical height–diameter (H-D) models) derived from the structure-explicit pathway modelling framework (see Methods). Variables are shown as circles: environmental variables on the left, structural attributes in the center, and AGB on the right. Structural attributes include basal area (BA), wood density weighted by basal area (WDba), quadratic mean diameter (DBHqm), and stem density (SD). Environmental variables include mean annual temperature (MAT), total annual precipitation (TAP), precipitation seasonality (PS), soil sand content (Sand), soil organic carbon content (Org. C), soil pH in H_2O (pH), and topographic slope (Slope). Moran's Eigenvector Maps, used to control for spatial autocorrelation, are not included in the figure. Arrows represent significant relationships; the absence of an arrow indicates no significant direct relationship (p-value ≥ 0.05). The values on each arrow correspond to standardized coefficients of the relationships, expressed as percentages relative to the mean of the response variable per standard deviation (%). For each response variable, the figure reports the R^2 value and the root mean square error (RMSE), expressed as a percentage of the mean response variable. Coefficients in raw, standardized, and % units are reported in Extended Data Table 6. For the three climate variables, values represent the average of the coefficients obtained using different climate data sources (see Methods). The same patterns held when using the continent- and region-specific H-D models of Feldpausch et al.[43] to estimate AGB (Extended Data Table 6).

Africa

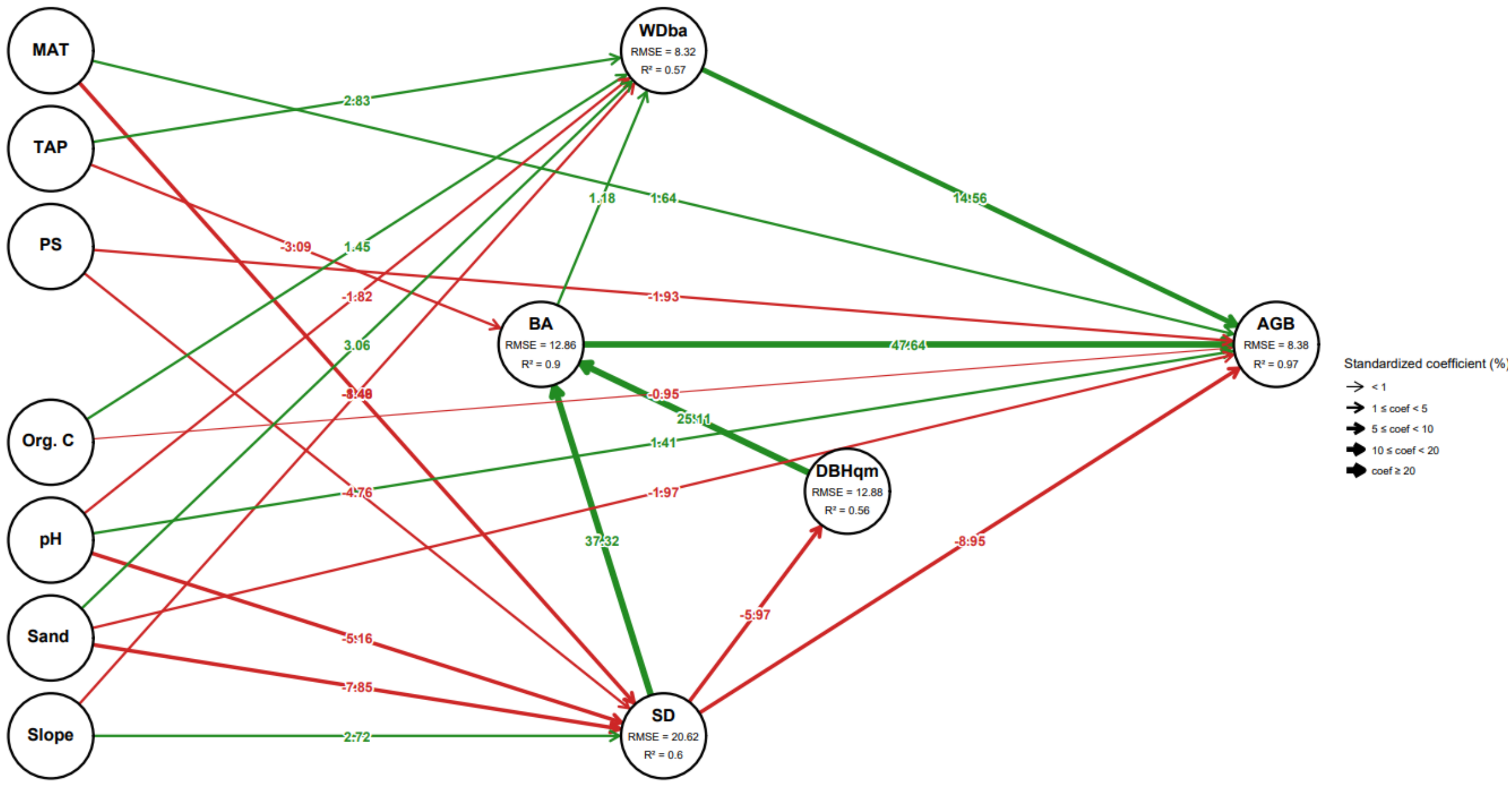

Extended Data Figure 17. Environmental effects on aboveground biomass (AGB) mediated by forest structure in Africa, including montane forests. Indirect and (residual) direct relationships between environmental variables and AGB (estimated using the pantropical height–diameter (H-D) models) derived from the structure-explicit pathway modelling framework (see Methods). Variables are shown as circles: environmental variables on the left, structural attributes in the center, and AGB on the right. Structural attributes include basal area (BA), wood density weighted by basal area (WDba), quadratic mean diameter (DBHqm), and stem density (SD). Environmental variables include mean annual temperature (MAT), total annual precipitation (TAP), precipitation seasonality (PS), soil sand content (Sand), soil organic carbon content (Org. C), soil pH in H_2O (pH), and topographic slope (Slope). Moran's Eigenvector Maps, used to control for spatial autocorrelation, are not included in the figure. Arrows represent significant relationships; the absence of an arrow indicates no significant direct relationship (p-value ≥ 0.05). The values on each arrow correspond to standardized coefficients of the relationships, expressed as percentages relative to the mean of the response variable per standard deviation (%). For each response variable, the figure reports the R^2 value and the root mean square error (RMSE), expressed as a percentage of the mean response variable. Coefficients in raw, standardized, and % units are reported in Extended Data Table 6. For the three climate variables, values represent the average of the coefficients obtained using different climate data sources (see Methods). The same patterns held when using the continent- and region-specific H-D models of Feldpausch et al.[43] to estimate AGB (Extended Data Table 6).

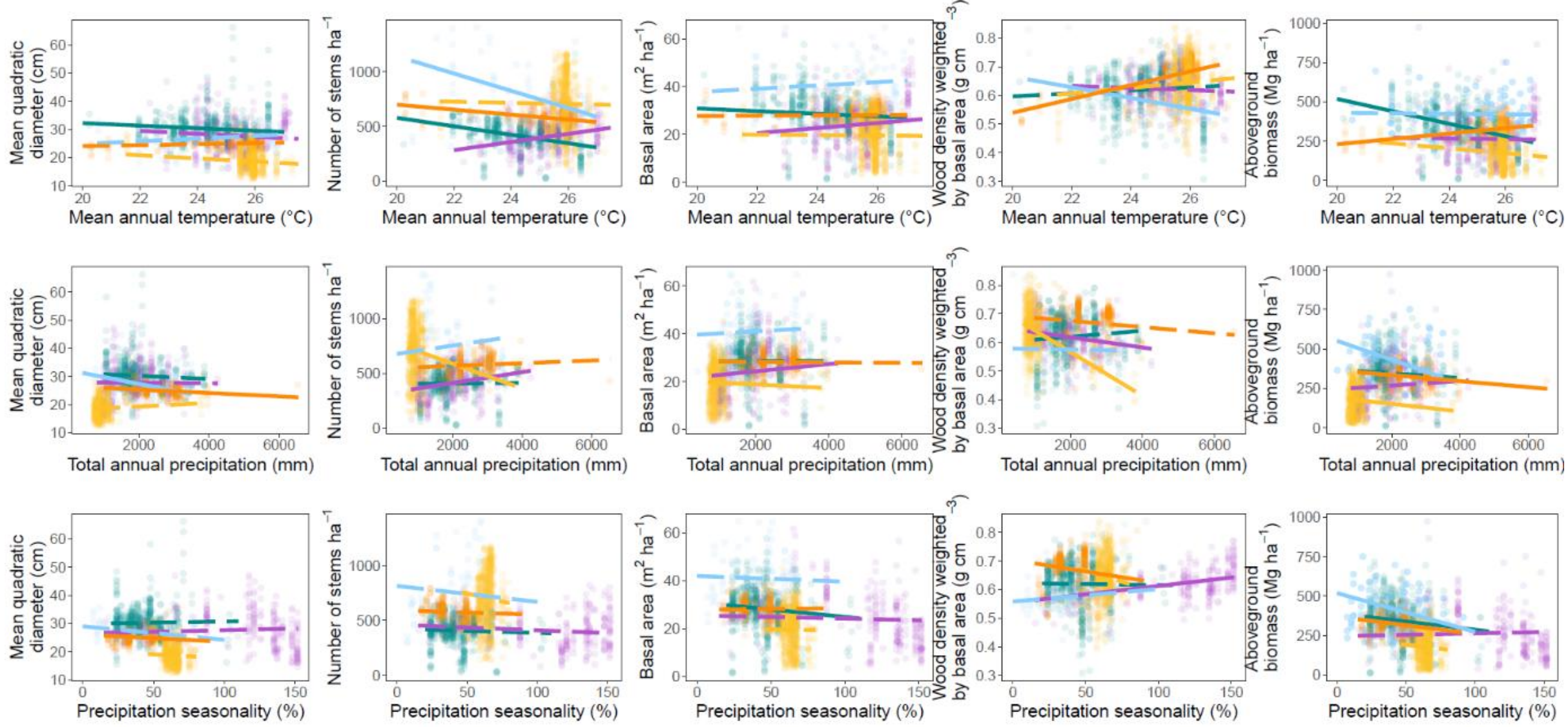

Extended Data Figure 18. Indirect and total relationships between climate variables and aboveground biomass (AGB), excluding montane forests (ERA5-Land dataset). The figure shows the bivariate relationships between selected climatic variables (x-axis) and forest structural attributes (y-axis), and the total bivariate relationships between selected climatic variables (x-axis) and AGB (y-axis), derived from the structure-explicit pathway modelling framework (see Methods). Solid lines indicate statistically significant relationships ($p < 0.05$), while dashed lines indicate non-significant relationships ($p \geq 0.05$). Colors correspond to each region as shown on the map (Figure 1). Results are shown for AGB estimated using tree height derived from the pantropical height-diameter (H-D) model of Feldpausch et al. (see Methods)[43].

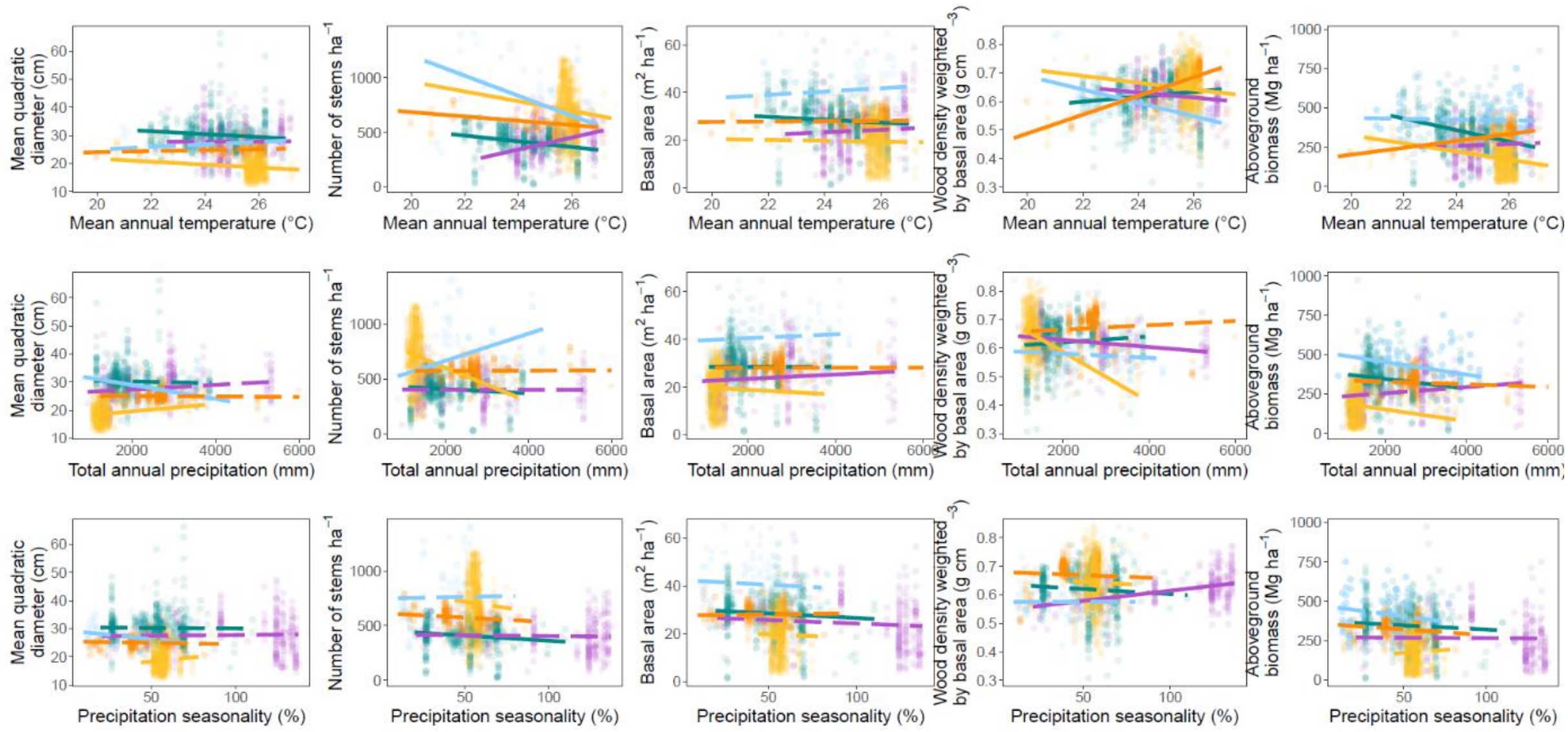

Extended Data Figure 19. Indirect and total relationships between climate variables and aboveground biomass (AGB), excluding montane forests (CHELSA dataset). The figure shows the bivariate relationships between selected climatic variables (x-axis) and forest structural attributes (y-axis), and the total bivariate relationships between selected climatic variables (x-axis) and AGB (y-axis), derived from the structure-explicit pathway modelling framework (see Methods). Solid lines indicate statistically significant relationships ($p < 0.05$), while dashed lines indicate non-significant relationships ($p \geq 0.05$). Colors correspond to each region as shown on the map (Figure 1). Results are shown for AGB estimated using tree height derived from the pantropical height-diameter (H-D) model of Feldpausch et al. (see Methods)[43].

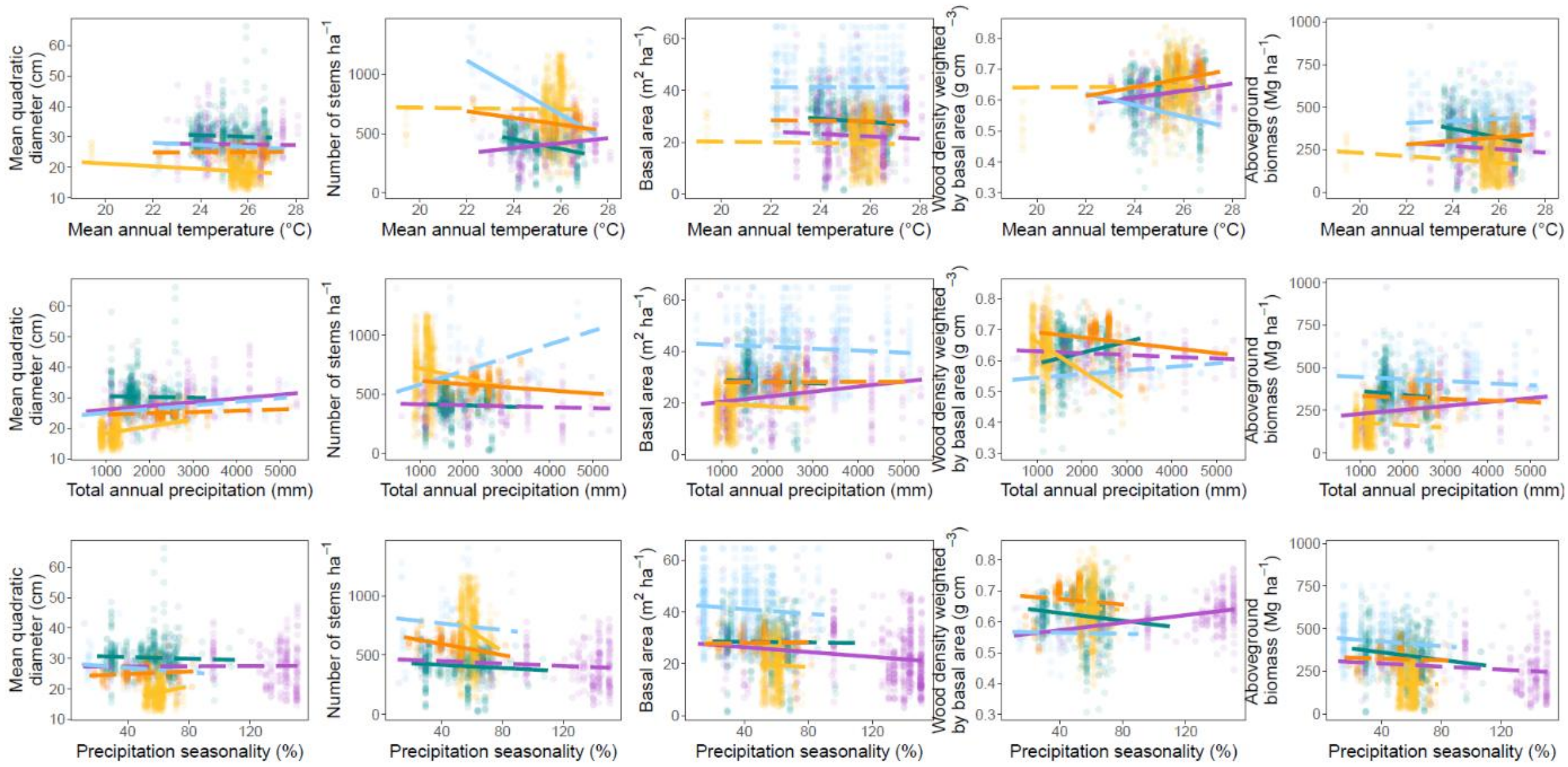

Extended Data Figure 20. Indirect and total relationships between climate variables and aboveground biomass (AGB), excluding montane forests (TerraClimate dataset). The figure shows the bivariate relationships between selected climatic variables (x-axis) and forest structural attributes (y-axis), and the total bivariate relationships between selected climatic variables (x-axis) and AGB (y-axis), derived from the structure-explicit pathway modelling framework (see Methods). Solid lines indicate statistically significant relationships ($p < 0.05$), while dashed lines indicate non-significant relationships ($p \geq 0.05$). Colors correspond to each region as shown on the map (Figure 1). Results are shown for AGB estimated using tree height derived from the pantropical height-diameter (H-D) model of Feldpausch et al. (see Methods)[43].

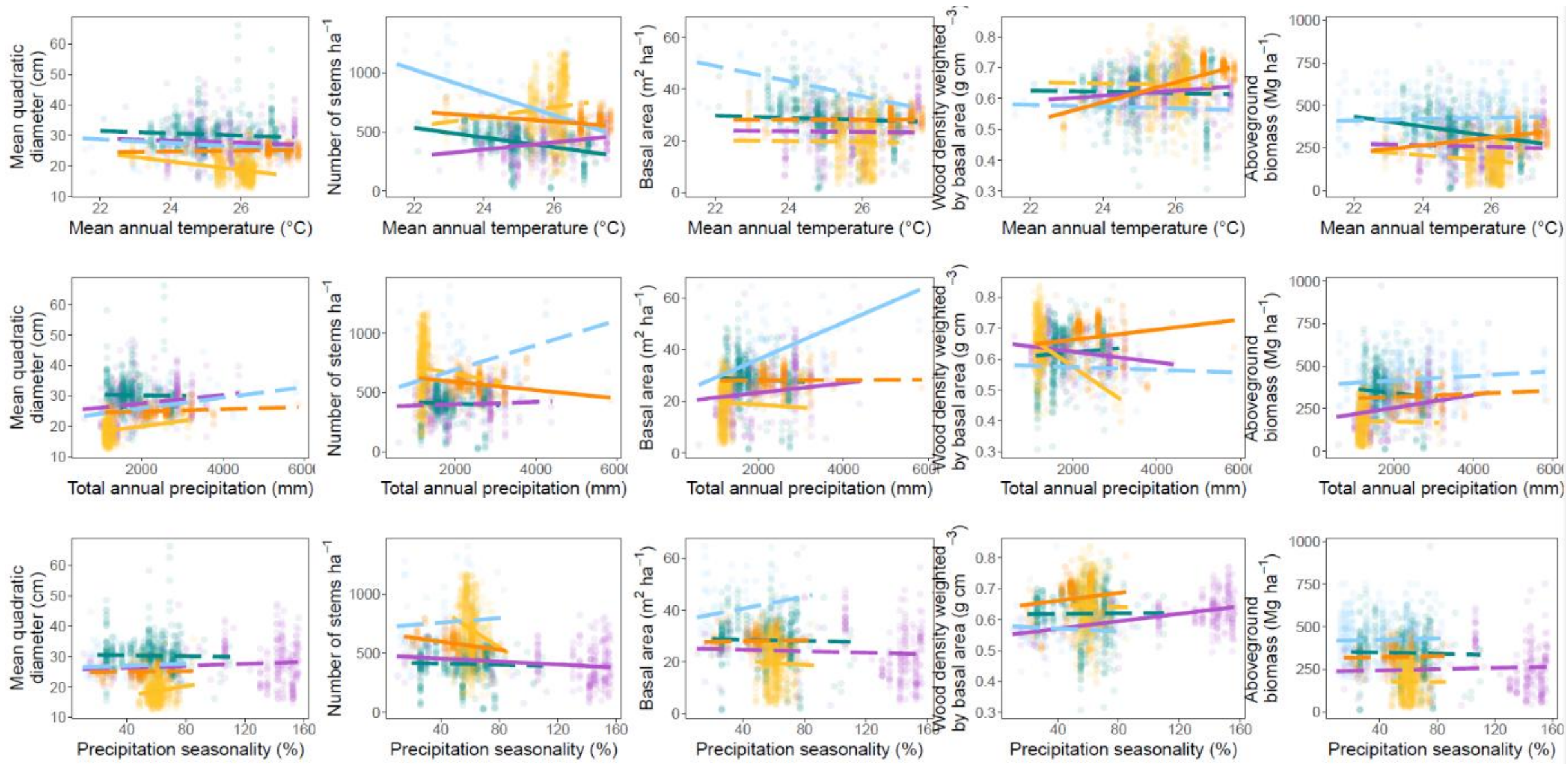

Extended Data Figure 21. Indirect and total relationships between climate variables and aboveground biomass (AGB), excluding montane forests (CRU TS dataset). The figure shows the bivariate relationships between selected climatic variables (x-axis) and forest structural attributes (y-axis), and the total bivariate relationships between selected climatic variables (x-axis) and AGB (y-axis), derived from the structure-explicit pathway modelling framework (see Methods). Solid lines indicate statistically significant relationships ($p < 0.05$), while dashed lines indicate non-significant relationships ($p \geq 0.05$). Colors correspond to each region as shown on the map (Figure 1). Results are shown for AGB estimated using tree height derived from the pantropical height-diameter (H-D) model of Feldpausch et al. (see Methods)[43].

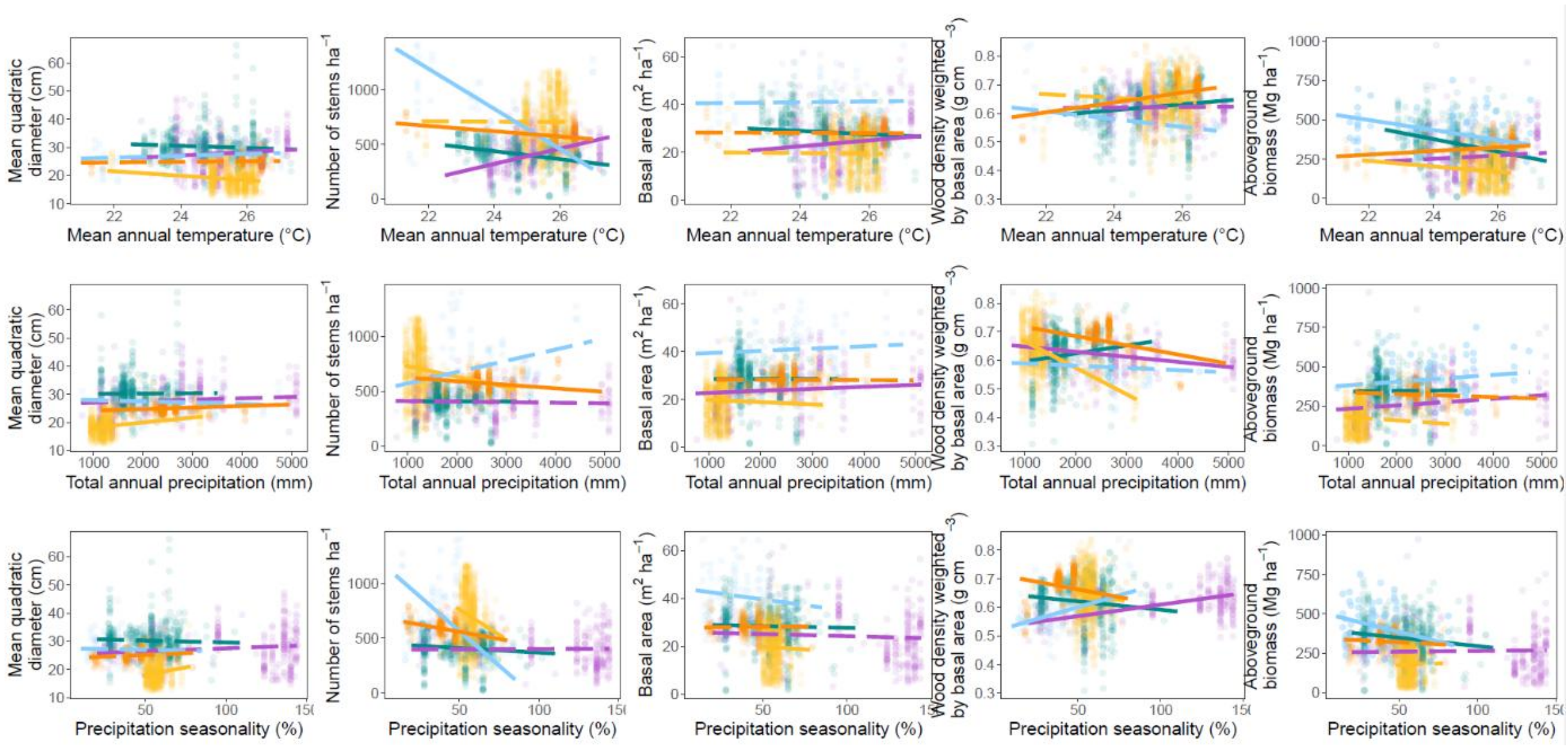

Extended Data Figure 22. Indirect and total relationships between climate variables and aboveground biomass (AGB), excluding montane forests (WorldClim dataset). The figure shows the bivariate relationships between selected climatic variables (x-axis) and forest structural attributes (y-axis), and the total bivariate relationships between selected climatic variables (x-axis) and AGB (y-axis), derived from the structure-explicit pathway modelling framework (see Methods). Solid lines indicate statistically significant relationships ($p < 0.05$), while dashed lines indicate non-significant relationships ($p \geq 0.05$). Colors correspond to each region as shown on the map (Figure 1). Results are shown for AGB estimated using tree height derived from the pantropical height-diameter (H-D) model of Feldpausch et al. (see Methods) [43].

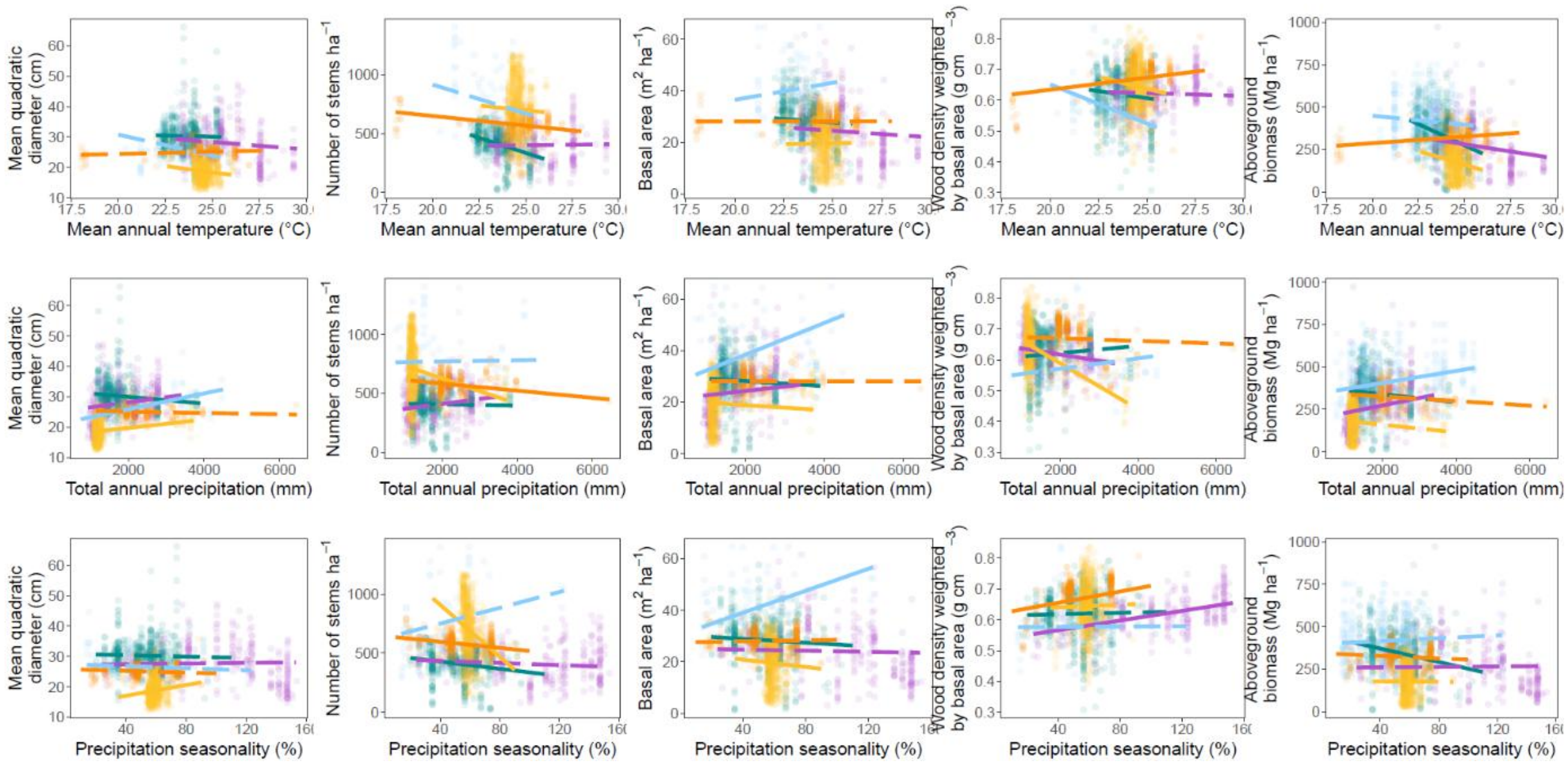

Extended Data Figure 23. Indirect and total relationships between climate variables and aboveground biomass (AGB), excluding montane forests (MODIS+IMERG dataset). The figure shows the bivariate relationships between selected climatic variables (x-axis) and forest structural attributes (y-axis), and the total bivariate relationships between selected climatic variables (x-axis) and AGB (y-axis), derived from the structure-explicit pathway modelling framework (see Methods). Solid lines indicate statistically significant relationships ($p < 0.05$), while dashed lines indicate non-significant relationships ($p \geq 0.05$). Colors correspond to each region as shown on the map (Figure 1). Results are shown for AGB estimated using tree height derived from the pantropical height-diameter (H-D) model of Feldpausch et al. (see Methods) [43].

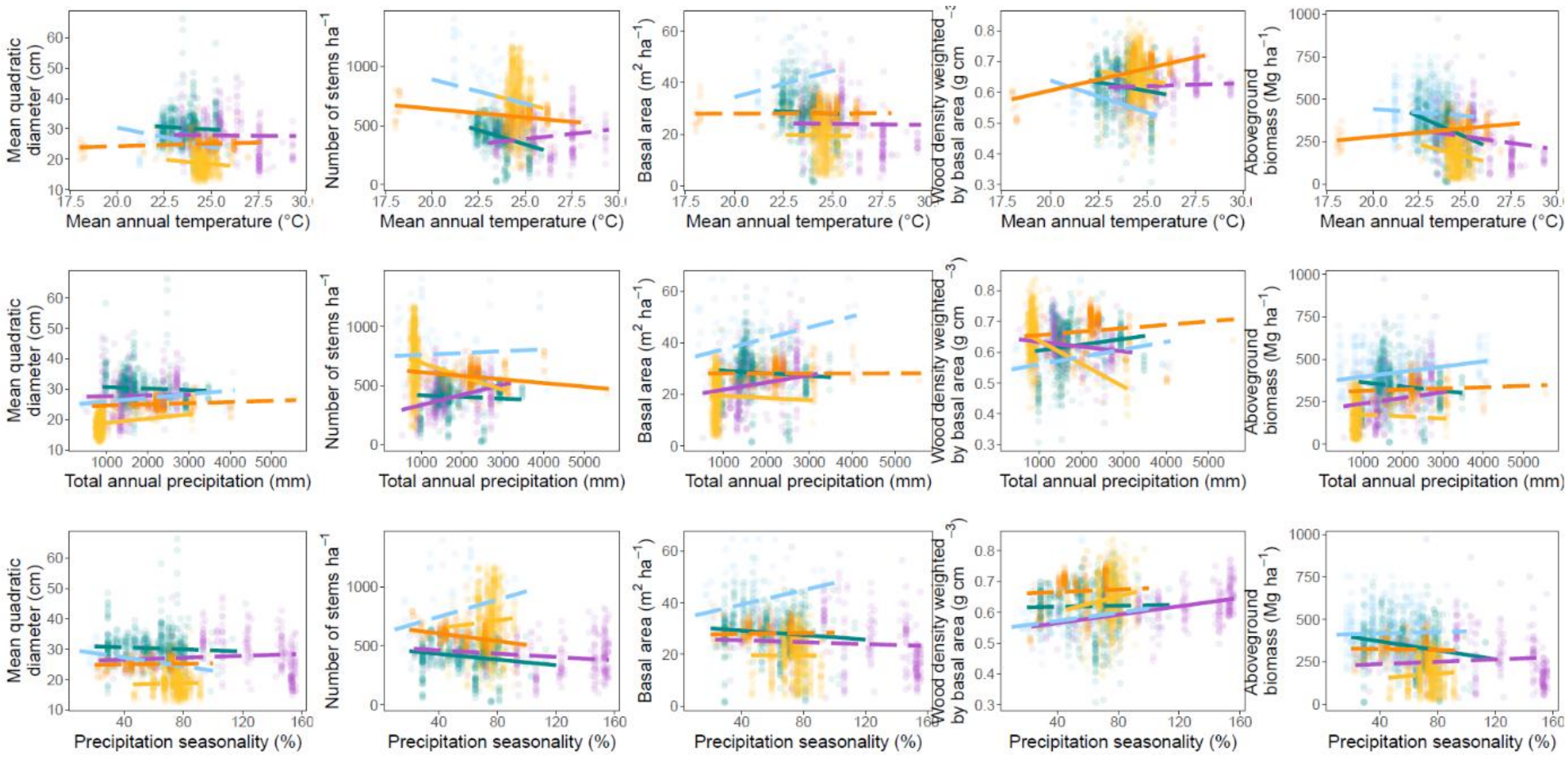

Extended Data Figure 24. Indirect and total relationships between climate variables and aboveground biomass (AGB), excluding montane forests (MODIS+CHIRPS dataset). The figure shows the bivariate relationships between selected climatic variables (x-axis) and forest structural attributes (y-axis), and the total bivariate relationships between selected climatic variables (x-axis) and AGB (y-axis), derived from the structure-explicit pathway modelling framework (see Methods). Solid lines indicate statistically significant relationships ($p < 0.05$), while dashed lines indicate non-significant relationships ($p \geq 0.05$). Colors correspond to each region as shown on the map (Figure 1). Results are shown for AGB estimated using tree height derived from the pantropical height-diameter (H-D) model of Feldpausch et al. (see Methods)[43].

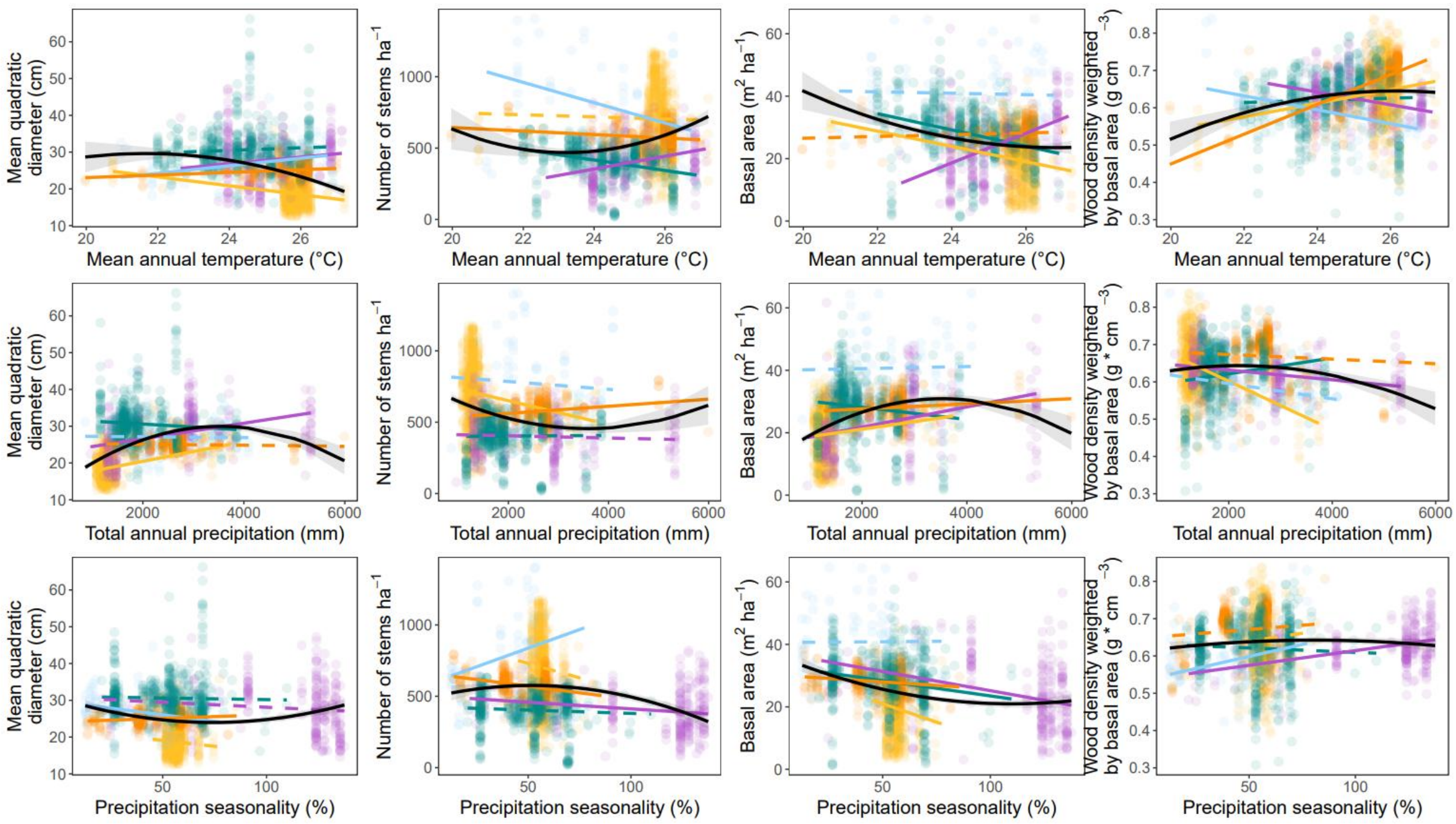

Extended Data Figure 25. Correlations between climate variables and structural attributes, excluding montane forests (CHELSA dataset). The figure shows correlations at the pantropical (black) and regional scales (colors correspond to the regions shown in Figure 1) between climatic variables (x-axis) and structural attributes (y-axis, see Methods). Solid lines indicate significant relationships ($p < 0.05$), whereas dashed lines indicate non-significant relationships ($p \geq 0.05$).

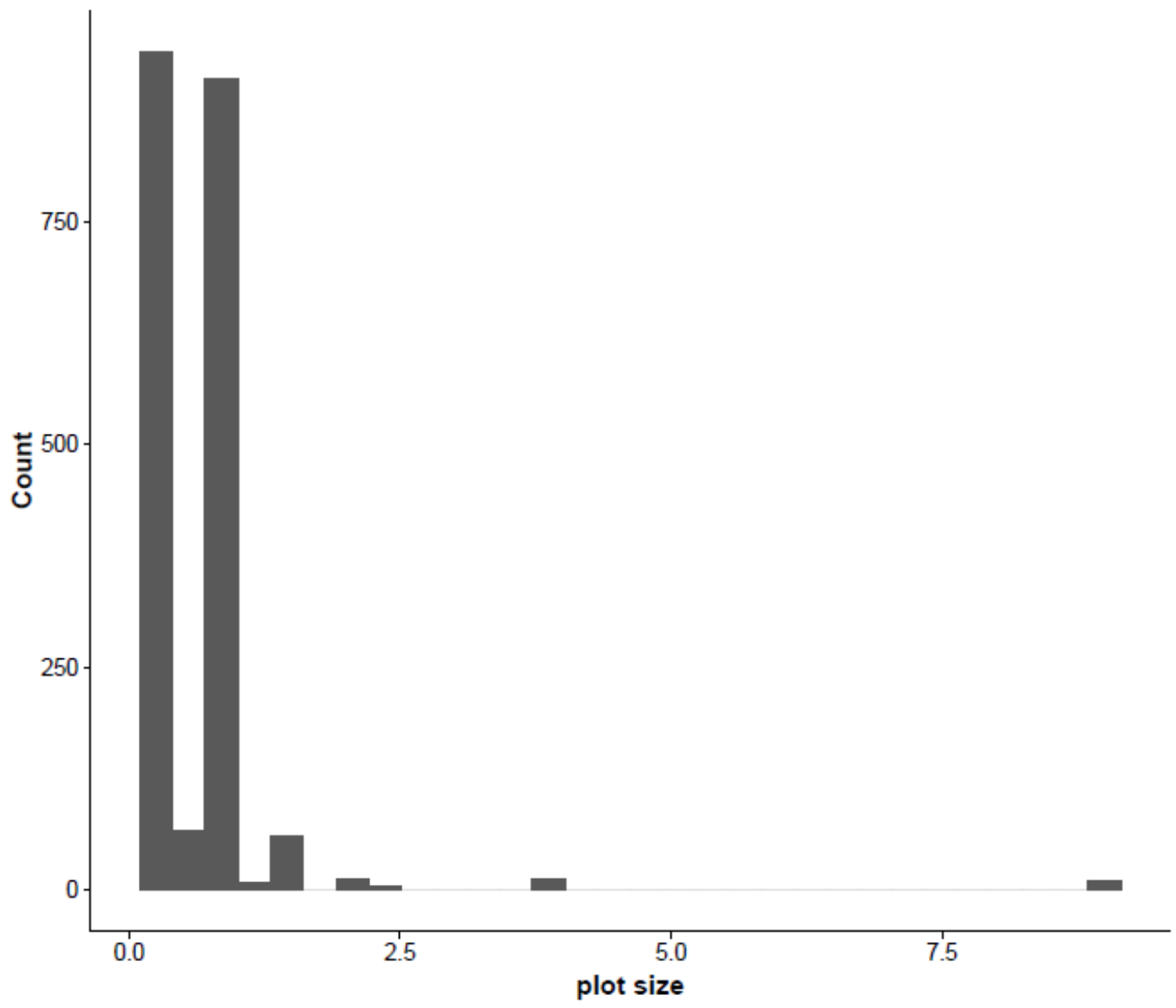

Extended Data Figure 26. Distribution of plot size. Histogram showing the frequency (count) of observations across plot-size classes.

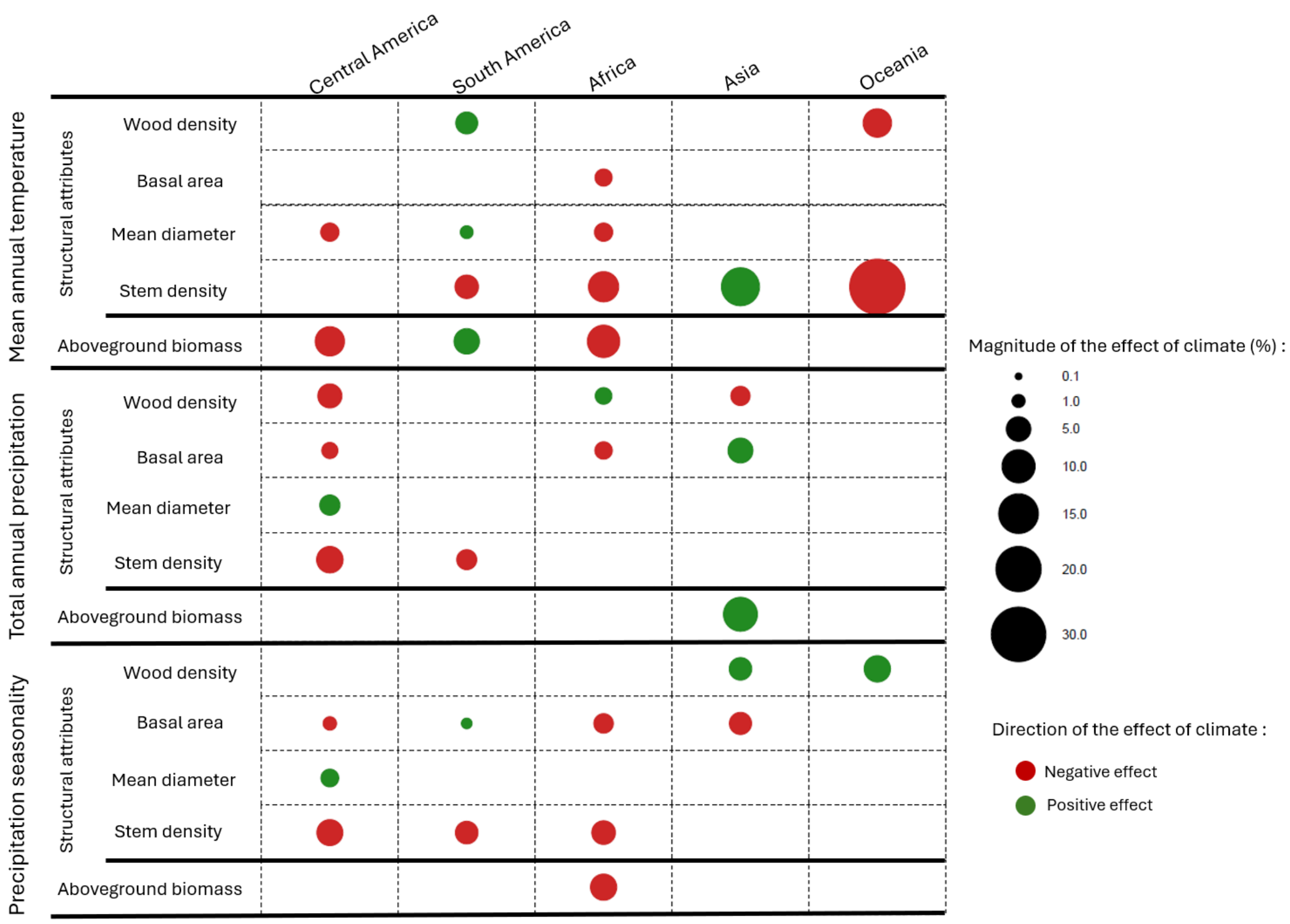

Extended Data Figure 27. Indirect effects and total effects of climate on aboveground biomass (AGB) across tropical regions, excluding montane forests. Standardized coefficients of relationships between climatic variables and structural attributes, and of total relationships between climatic variables and AGB, as identified by the structure-explicit pathway modelling framework (see Methods). Columns correspond to the regions studied. Coefficients are shown as colored circles, expressed as percentages relative to the mean of the response variable per standard deviation (%). Circle size reflects the magnitude of each effect, with values for the three climatic variables representing the average coefficients obtained across different climate data sources (see Methods). Green indicates positive effects, red indicates negative effects. Empty boxes indicate non-significant coefficients ($p \geq 0.05$). Results are shown for AGB estimated using tree height derived from the pantropical height-diameter (H-D) model of Feldpausch et al. (see Methods) [43]. For Africa, results correspond to the model without control for the percentage of pioneer species (see Methods).

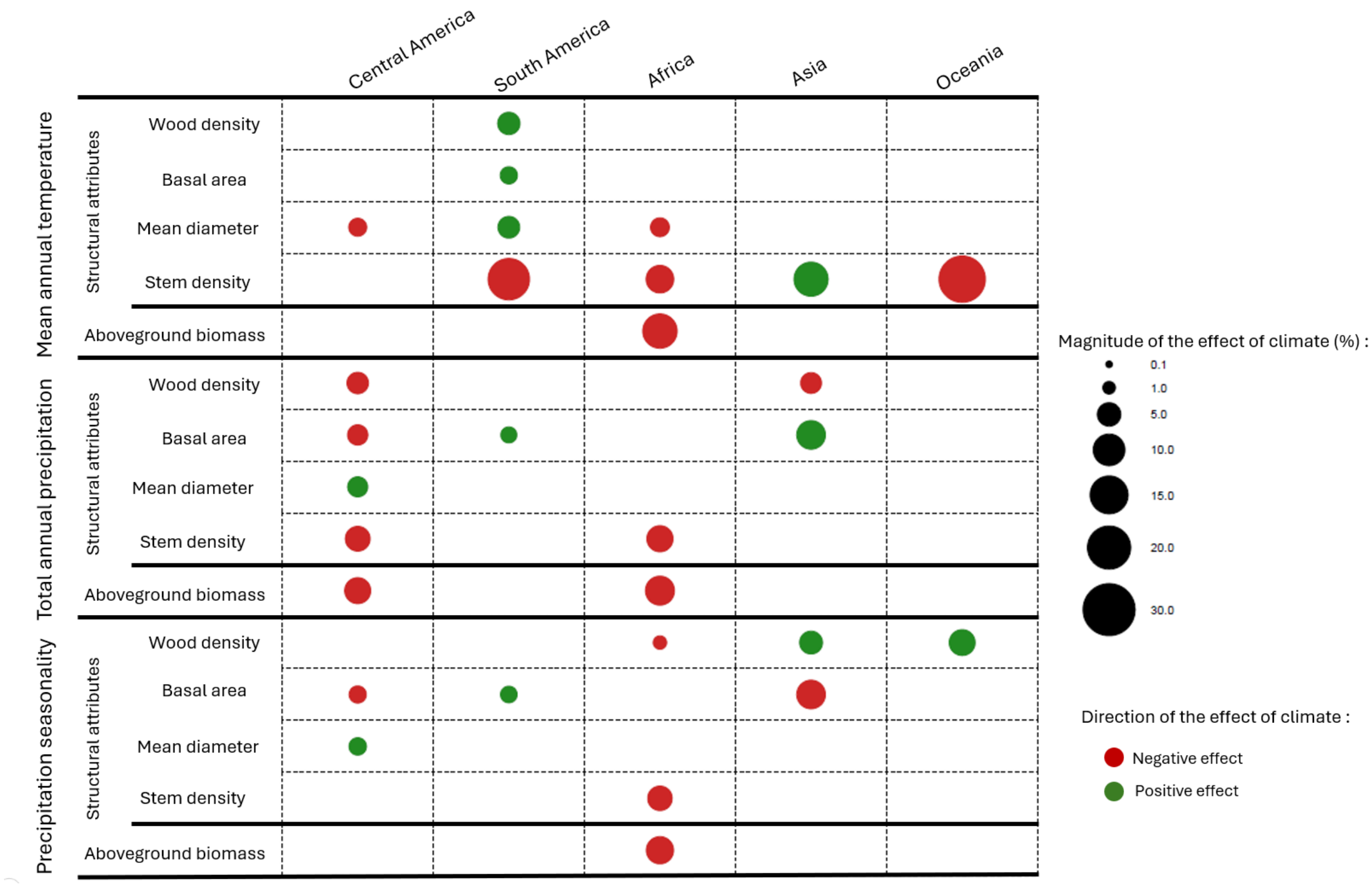

Extended Data Figure 28. Indirect effects and total effects of climate on aboveground biomass (AGB) across tropical regions, excluding montane forests and including all plots without trimming of extreme stem density and AGB values. Standardized coefficients of relationships between climatic variables and structural attributes, and of total relationships between climatic variables and AGB, as identified by the structure-explicit pathway modelling framework (see Methods). Columns correspond to the regions studied. Coefficients are shown as colored circles, expressed as percentages relative to the mean of the response variable per standard deviation (%). Circle size reflects the magnitude of each effect, with values for the three climatic variables representing the average coefficients obtained across different climate data sources (see Methods). Green indicates positive effects, red indicates negative effects. Empty boxes indicate non-significant coefficients ($p \geq 0.05$). Results are shown for AGB estimated using tree height derived from the pantropical height-diameter (H-D) model of Feldpausch et al. (see Methods) [43]. For Africa, results correspond to the model without control for the percentage of pioneer species (see Methods).

Tables.

Extended Data Table 1: Filtering process for field inventories. This table summarizes the data cleaning steps applied to the forest inventory datasets and the corresponding number of plots retained at each step. Field inventories were provided by ForestPlots.net[35-37], the Global Forest Biodiversity Initiative[38] (GFBI, via Science-i web platform, https://www.gfbinitiative.org/) which includes long-term plot data from Panama[39-41], AfriTRON (https://afritron.org/) and RAINFOR (https://rainfor.org/en/) networks, and by direct contributions from principal investigators (PIs) of field plots.

Filtering steps	Number of plots			
	Source			Number of plots retained
	GFBI	ForestPlots.net	Directly from PIs	
Initial dataset compilation	18,404	213	856	19,473
Aggregation of plots divided into four subplots	18,377	213	856	19,446
Removal of plots with multiple registered sizes or missing size information	13,162	213	824	14,199
Removal of trees with dbh < 10 cm	12,370	212	818	13,400
Removal of plots in which ≥5% of trees are missing dbh	11,405	212	816	12,433
Removal of plots smaller than 0.25 hectares	3,379	197	815	4,391
Removal of plots with less than 90% of trees identified at the genus level	3,234	185	744	4,163
Removal of plots with multiple recorded locations	3,214	185	744	4,143
Removal of plots not located in tropical moist broadleaf forests based on the RESOLVE Ecoregions dataset[42]	1,739	178	680	2,597
Detection and resolution of overlapping plots within and between sources	1,515	178	680	2,373
Removal of the lower and upper 3% outliers for stem density	1,375	158	645	2,178
Removal of the lower and upper 2% outliers for AGB	1,309	146	621	2,076
Exclusion of non–old-growth plots based on field inventories	1,309	121	596	2,026
Extraction of environmental data for each plot				**2,023**

Extended Data Table 2: Definitions of each structural attribute and aboveground biomass. This table provides the definition, calculation (for a one-hectare plot), and the unit of each structural attribute and aboveground biomass.

Structural attributes	Definition	Calculation	Unit
**Basal area (BA)**	Sum of all the tree sections present in the plot, divided by the size of the plots.	$\frac{\sum_{i=1}^{n} Section_i}{PS} = \frac{\sum_{i=1}^{n}\left(\frac{\left(\frac{DBH_i}{100}\right)}{2}\right)^2 * \pi}{PS}$<br>Where:<br>i= ith tree<br>n= number of trees in the plot<br>$Section_i$=section of the ith tree [m^2]<br>DBH_i = diameter at breast height of ith tree [cm]<br>PS= plot size [ha]	$m^2\ ha^{-1}$
**Community wood density weighted by the BA (WDba)**	Sum of the wood density (the oven dry weight divided by green volume of wood, [g cm^{-3}]) of all the trees, present in the plot, each of them multiplied by the tree section divided by the BA of the plot.	$\sum_{i=1}^{n} WD_i * \frac{Section_i}{BA}$<br>Where:<br>i= ith tree<br>n= number of trees in the plot<br>WD_i = wood density of ith tree [g cm^{-3}]<br>$Section_i$=section of the ith tree [m^2]<br>BA= basal area of the plot [m^2]	g cm^{-3}
**Stem density (SD)**	Number of stems within the plot, divided by the size of the plot.	$\frac{\sum_{i=1}^{n} Stem_i}{PS}$<br>Where:<br>i= ith tree<br>n= number of trees in the plot<br>$Stem_i$= ith stem<br>PS= plot size [ha]	Number of stems ha^{-1}
**Mean quadratic diameter (DBHqm)**	Quadratic mean of the DBHs (diameter at breast-height) of the trees present in the plot.	$\sqrt{\frac{1}{n} * \sum_{i=1}^{n} DBH_i^2}$<br>Where:<br>i= ith tree<br>n= number of trees in the plot<br>DBH_i = diameter at breast height of ith tree [cm]	cm
**Aboveground biomass (AGB)**	Sum of the AGB of all the trees present in the plot, divided by the size of the plot.	$AGB = \frac{\sum_{i=1}^{n} AGB_i}{PS}$<br>Where:<br>i= ith tree<br>n= number of trees in the plot<br>PS= plot size [ha]<br>AGB_i = AGB of ith tree [Mg]<br><br>where the AGB of the tree is calculated using equation 4 of Chave et al. 2014[49]:<br><br>$AGB_i=0.0673\times(WD\times DBH^2\times H)^{0.976}$<br>Where:<br>WD= wood density of the tree [g cm^{-3}]<br>DBH= diameter at breast height [cm]<br>H = total tree height [m] estimated using the pantropical, continent- and region-specific H-D models by Feldpausch et al.[43]: H=a(1−exp(−bDc))<br>where parameter values (a, b, c)for pantropical, continent-specific, and region-specific models are reported in Table 3 of Feldpausch et al. (2012)[43].	Mg ha^{-1}

Extended Data Table 3. Environmental datasets. This table provides the list of environmental datasets used in the study, including climate, soil, and topographic sources. For each climate dataset, the three bioclimatic variables were calculated following the WorldClim methodology, averaged over the 30-year period 1981–2010, and upscaled to a 0.5° spatial resolution, primarily using Google Earth Engine[68], to match the resolution of the CRU TS dataset (see Methods). The "Download source" column indicates whether the dataset was accessed via Google Earth Engine or directly downloaded from the original source website (link provided).

Category	Dataset	Resolution (°)	Download source
Climate	WorldClim v2.1	Downscaled to 0.5°	https://www.worldclim.org/data/worldclim21.html
Climate	ERA5-Land Monthly Aggregated-ECMWF Climate reanalysis	Downscaled to 0.5°	Google Earth Engine
Climate	TerraClimate	Downscaled to 0.5°	Google Earth Engine
Climate	CHELSA V2.1	Downscaled to 0.5°	https://chelsa-climate.org/downloads/
Climate	CRU TS 4.06	0.5°	https://crudata.uea.ac.uk/cru/data/hrg/
Climate	MODIS MYD11A2 V6.1	Downscaled to 0.5°	Google Earth Engine
Climate	CHIRPS V2.0	Downscaled to 0.5°	Google Earth Engine
Climate	IMERG V6.0	Downscaled to 0.5°	Google Earth Engine
Soil	SoilGrids v2.0 (OpenLandMap/SOL)	250 m	Google Earth Engine
Topography	NASA SRTM Digital Elevation	30 m	Google Earth Engine
